# Supplementary figures and images for: Importance of glutamine in synaptic vesicles revealed by functional studies of SLC6A17 and its mutations pathogenic for intellectual disability
Source: eLife. 2023 Jul 13;12:RP86972. doi: 10.7554/eLife.86972 (PMC10393021; doi:10.7554/eLife.86972)

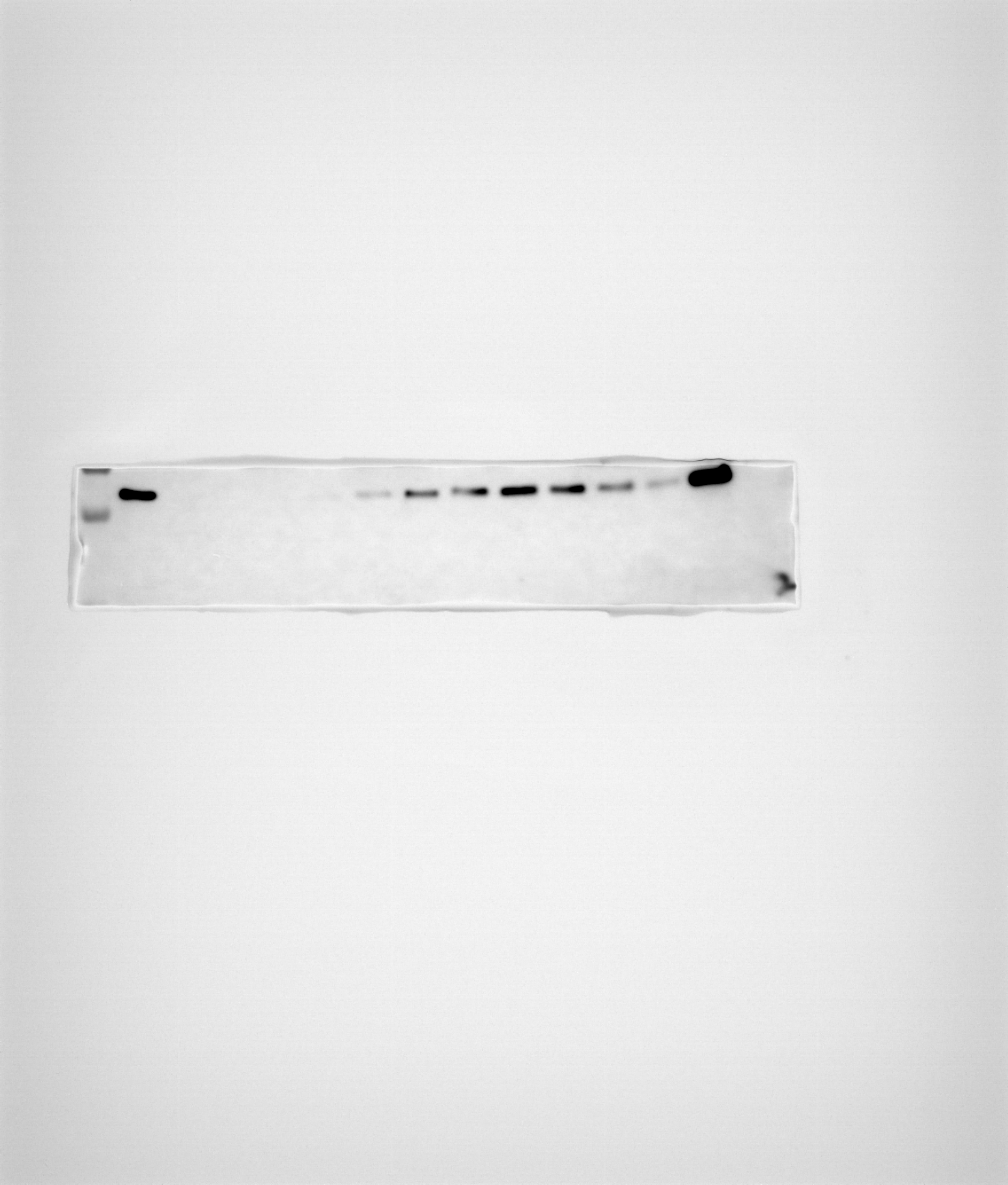

Supplement: Figure 3—source data 1. [file elife-86972-fig3-data1.zip › Figure 3-S1D/CalR.tif]

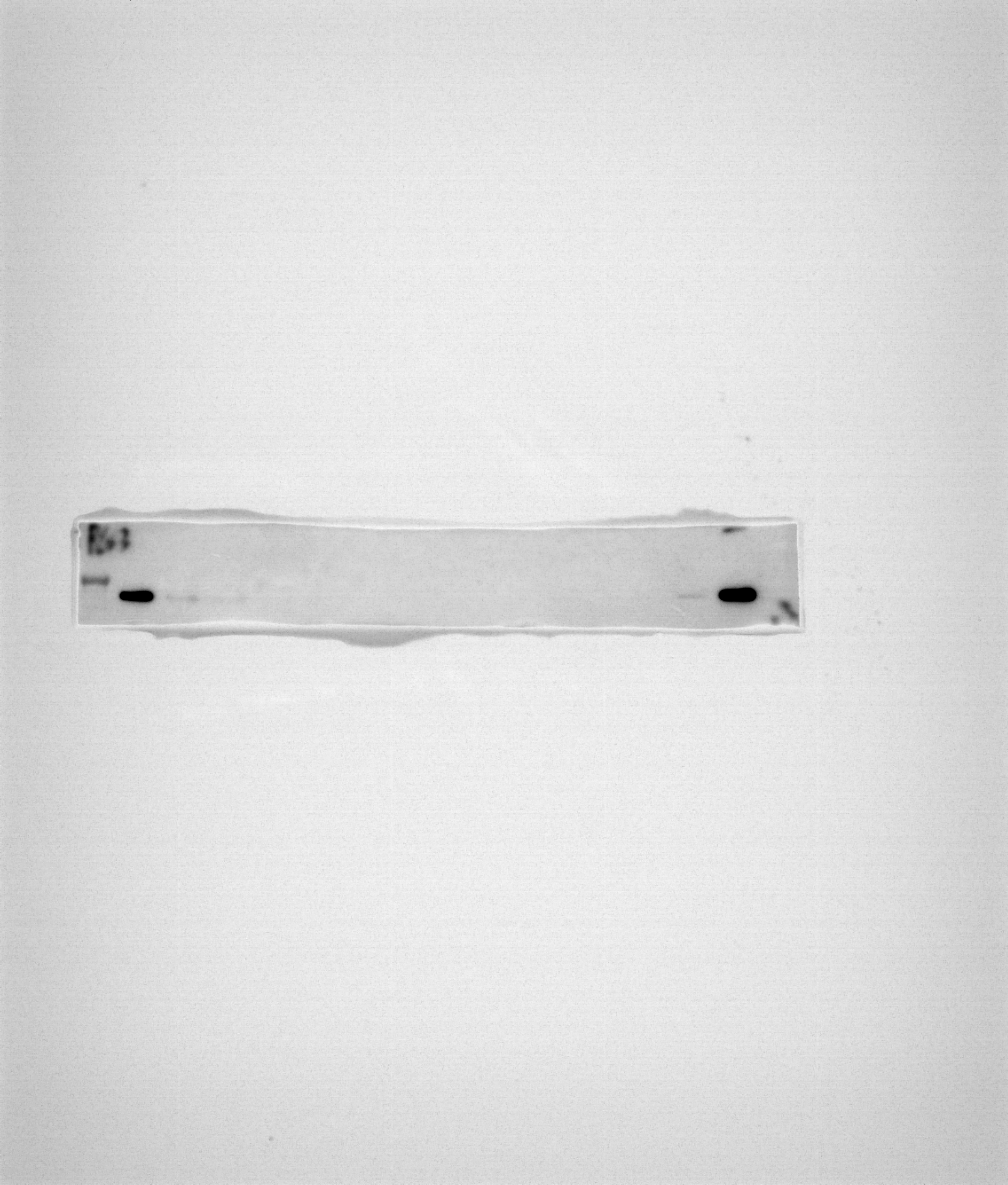

Supplement: Figure 3—source data 1. [file elife-86972-fig3-data1.zip › Figure 3-S1D/EEA1.tif]

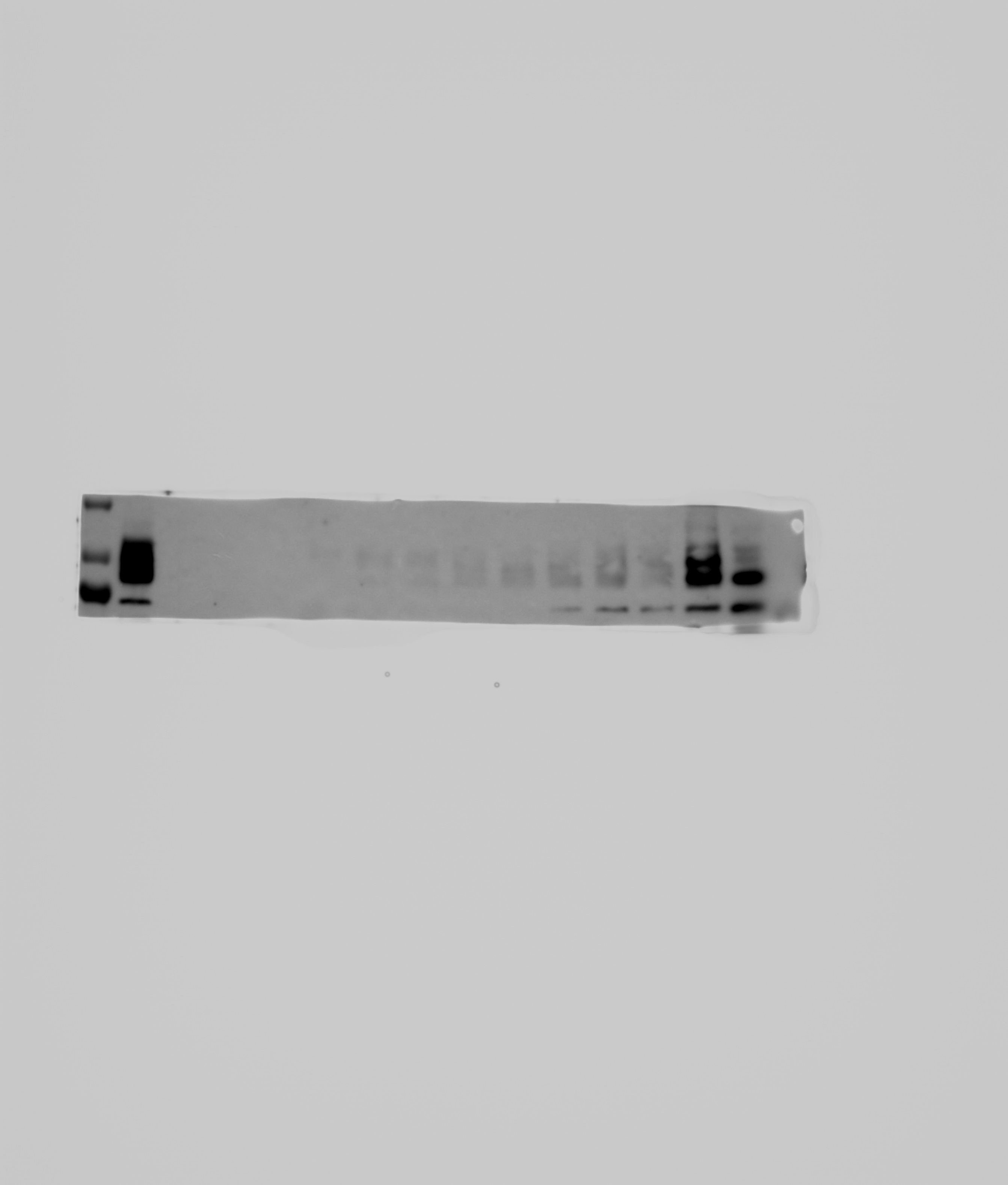

Supplement: Figure 3—source data 1. [file elife-86972-fig3-data1.zip › Figure 3-S1D/FLAG.tif]

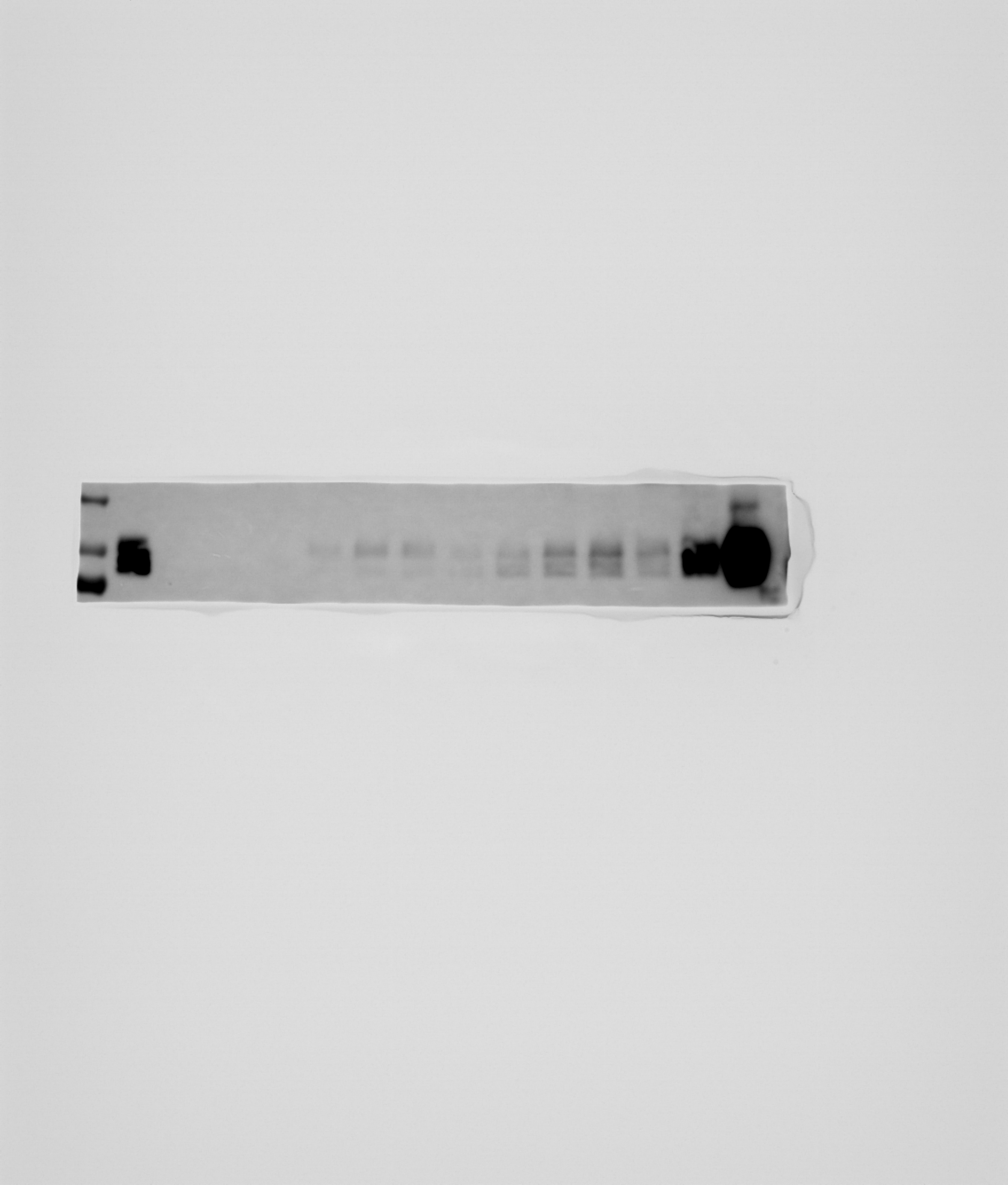

Supplement: Figure 3—source data 1. [file elife-86972-fig3-data1.zip › Figure 3-S1D/HA.tif]

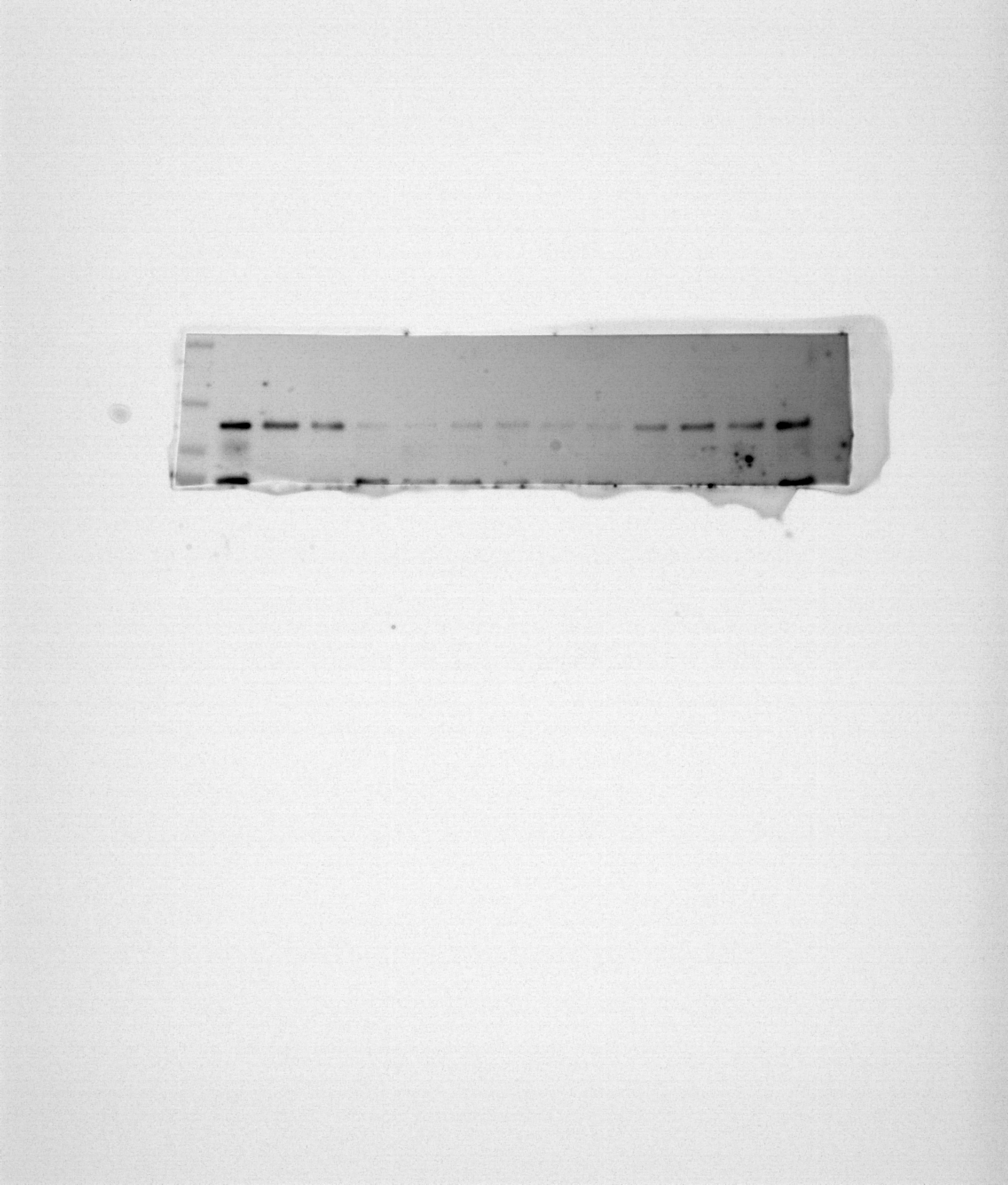

Supplement: Figure 3—source data 1. [file elife-86972-fig3-data1.zip › Figure 3-S1D/LAMP2-s.tif]

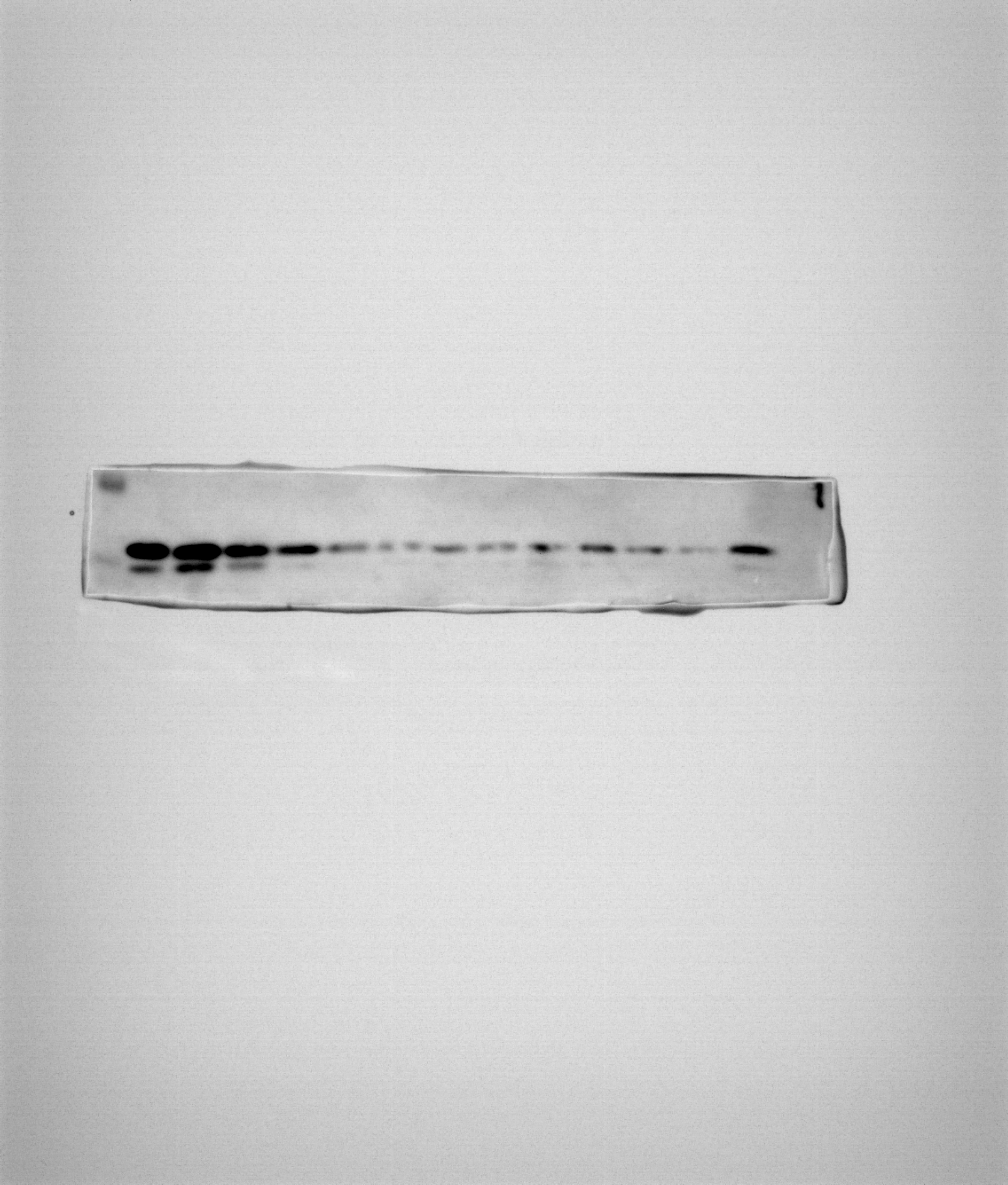

Supplement: Figure 3—source data 1. [file elife-86972-fig3-data1.zip › Figure 3-S1D/LC3B-S.tif]

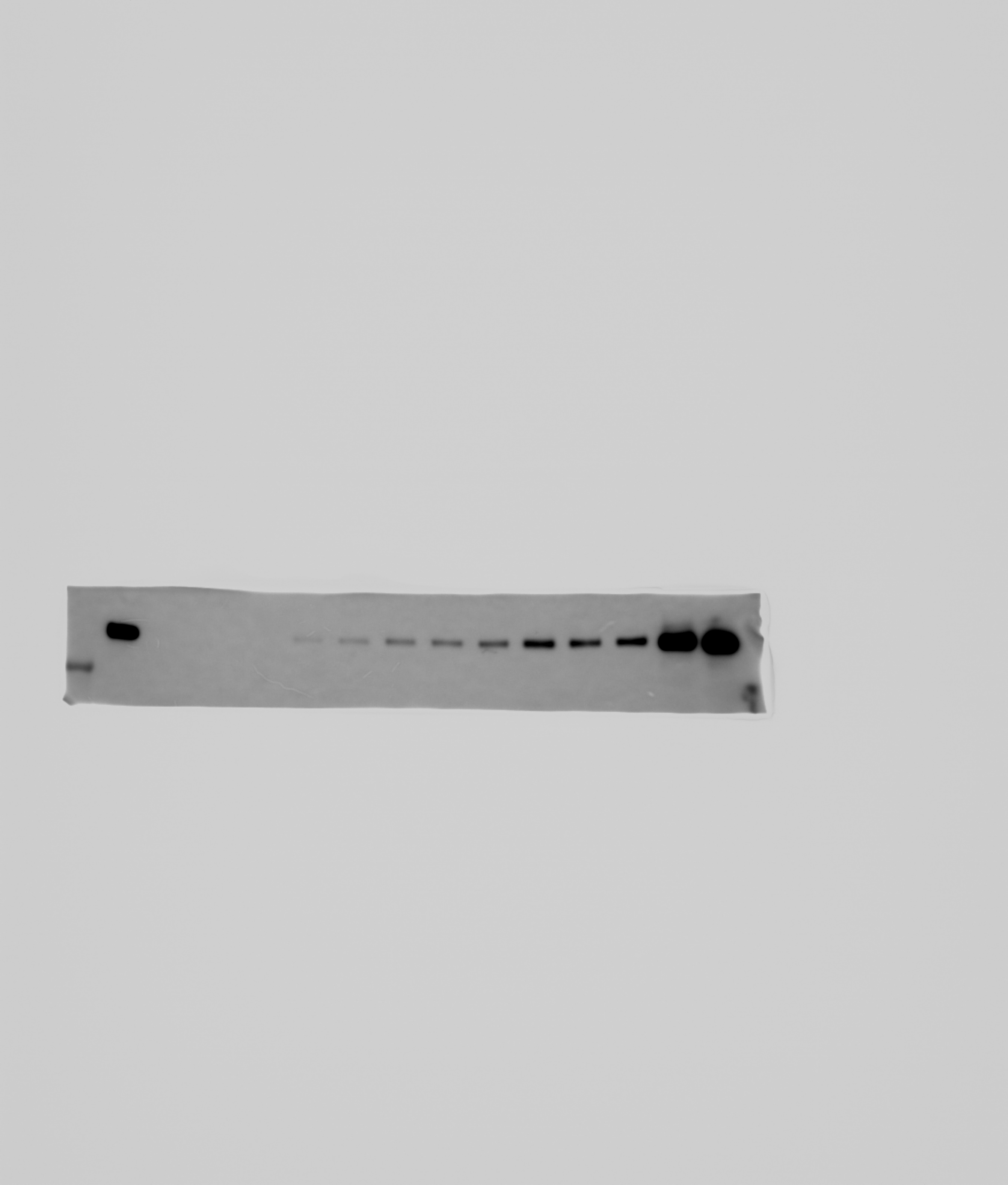

Supplement: Figure 3—source data 1. [file elife-86972-fig3-data1.zip › Figure 3-S1D/M6PR.tif]

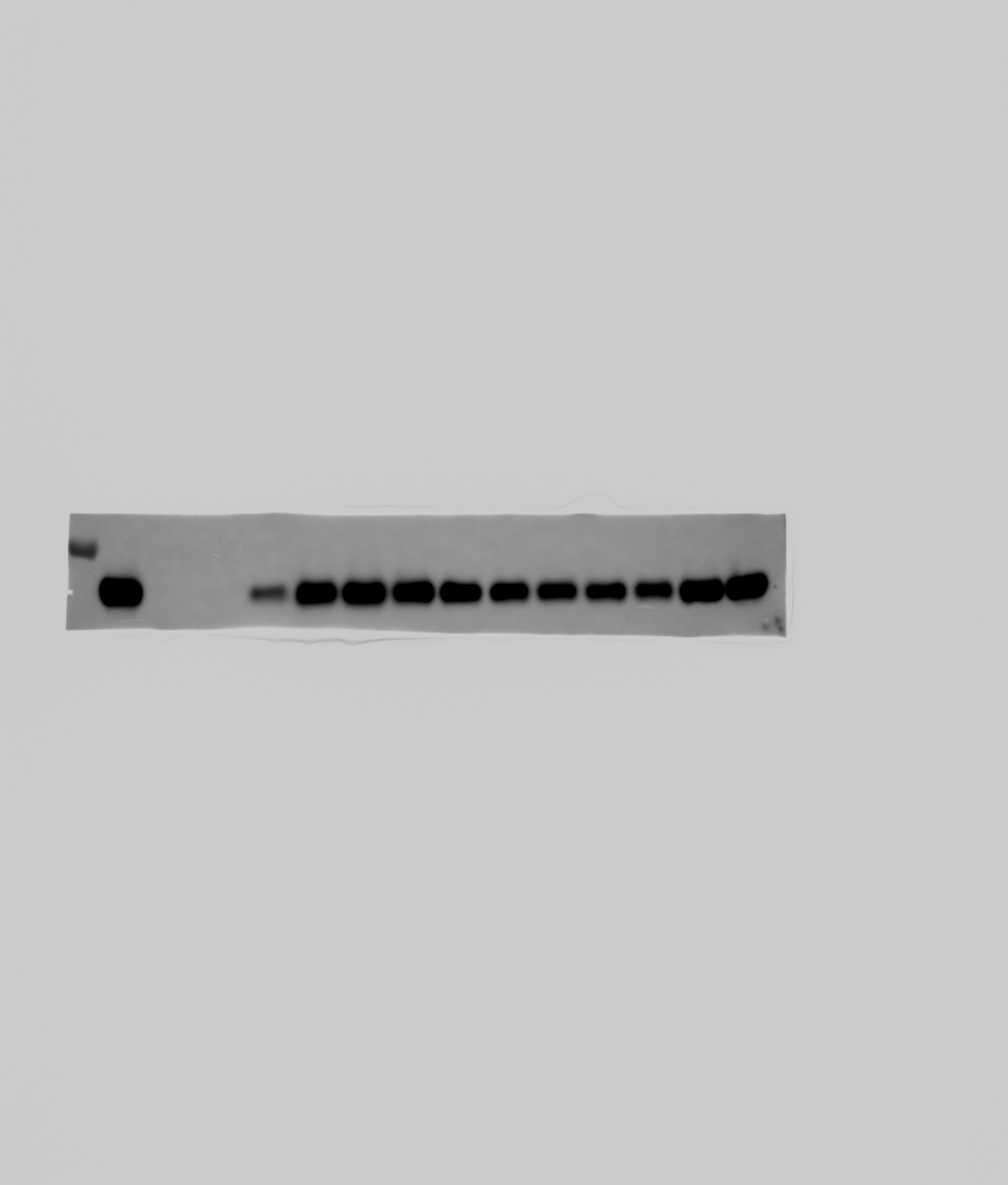

Supplement: Figure 3—source data 1. [file elife-86972-fig3-data1.zip › Figure 3-S1D/Syp.tif]

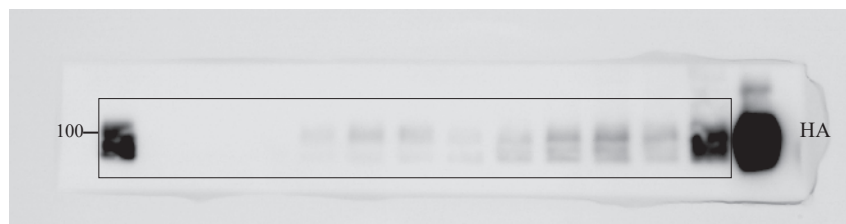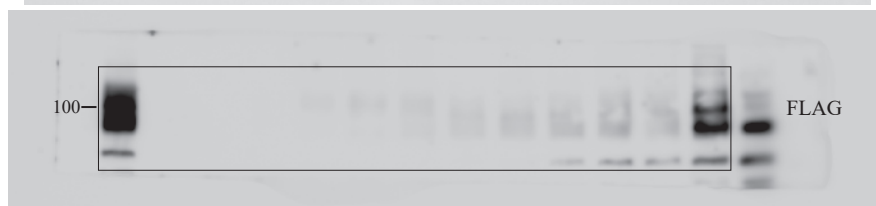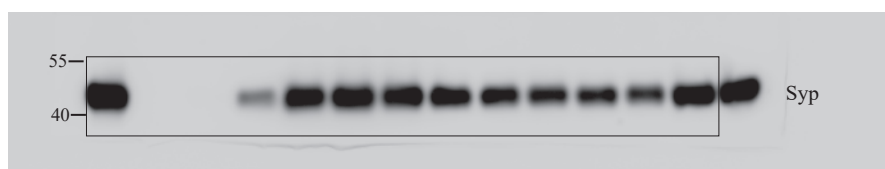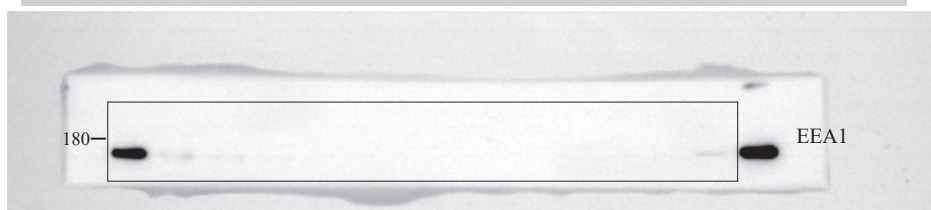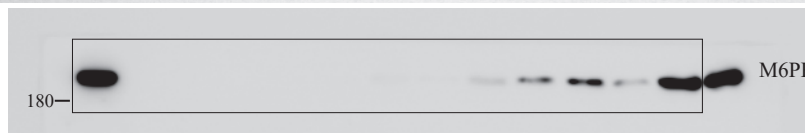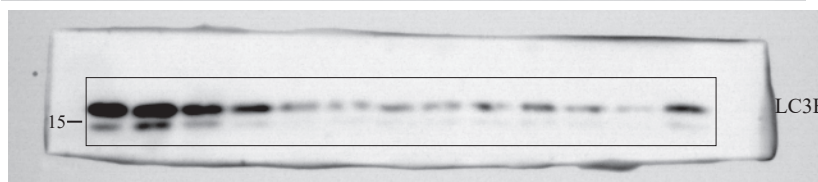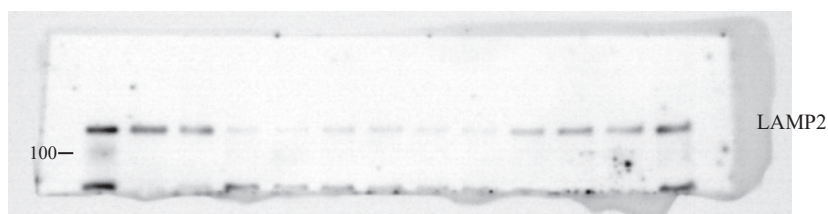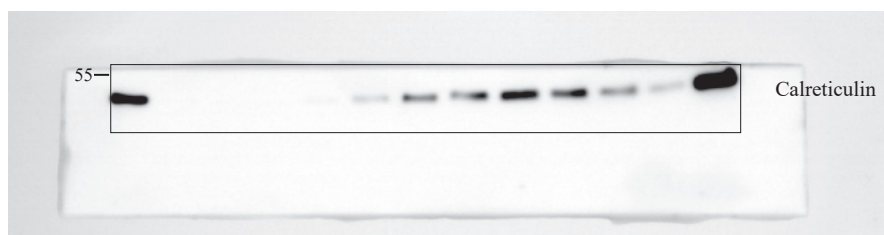

Supplement: Figure 3—figure supplement 1—source data 2. [file elife-86972-fig3-figsupp1-data2.zip › FigureS2D-Source Data-WB.pdf]

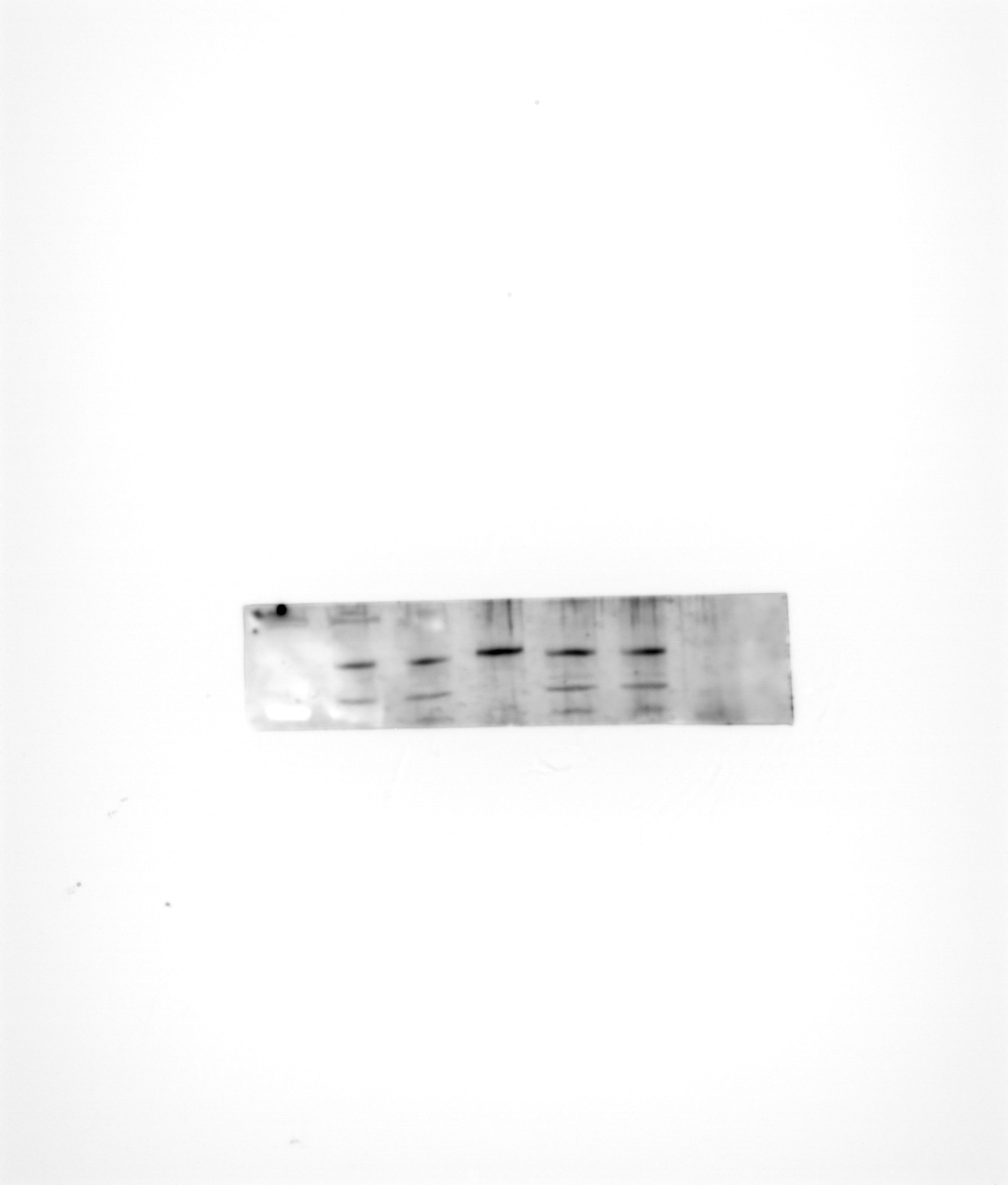

Supplement: Figure 4—source data 1. [file elife-86972-fig4-data1.zip › Figure 4C/ATPase (2).tif]

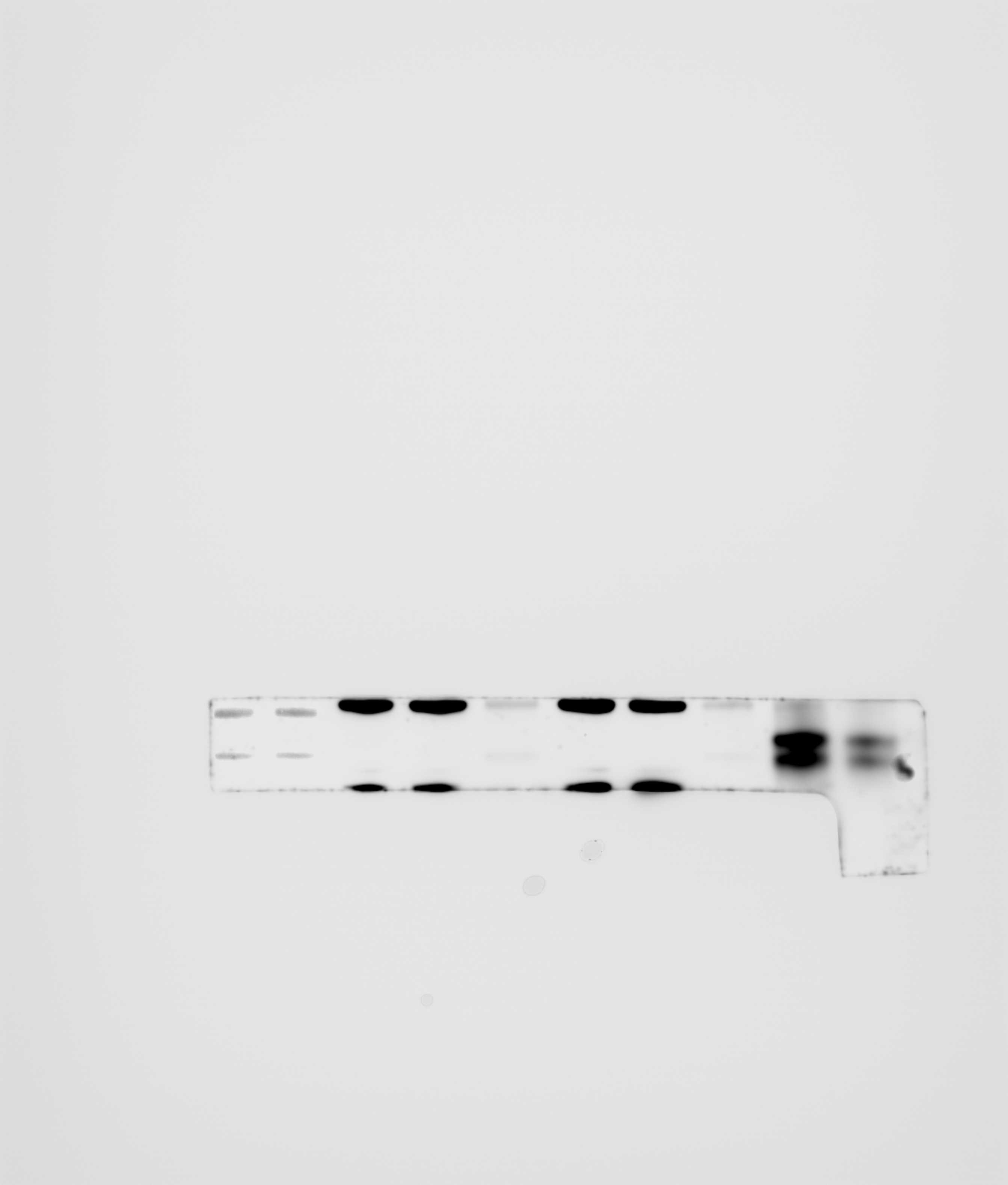

Supplement: Figure 4—source data 1. [file elife-86972-fig4-data1.zip › Figure 4C/GLUT4.tif]

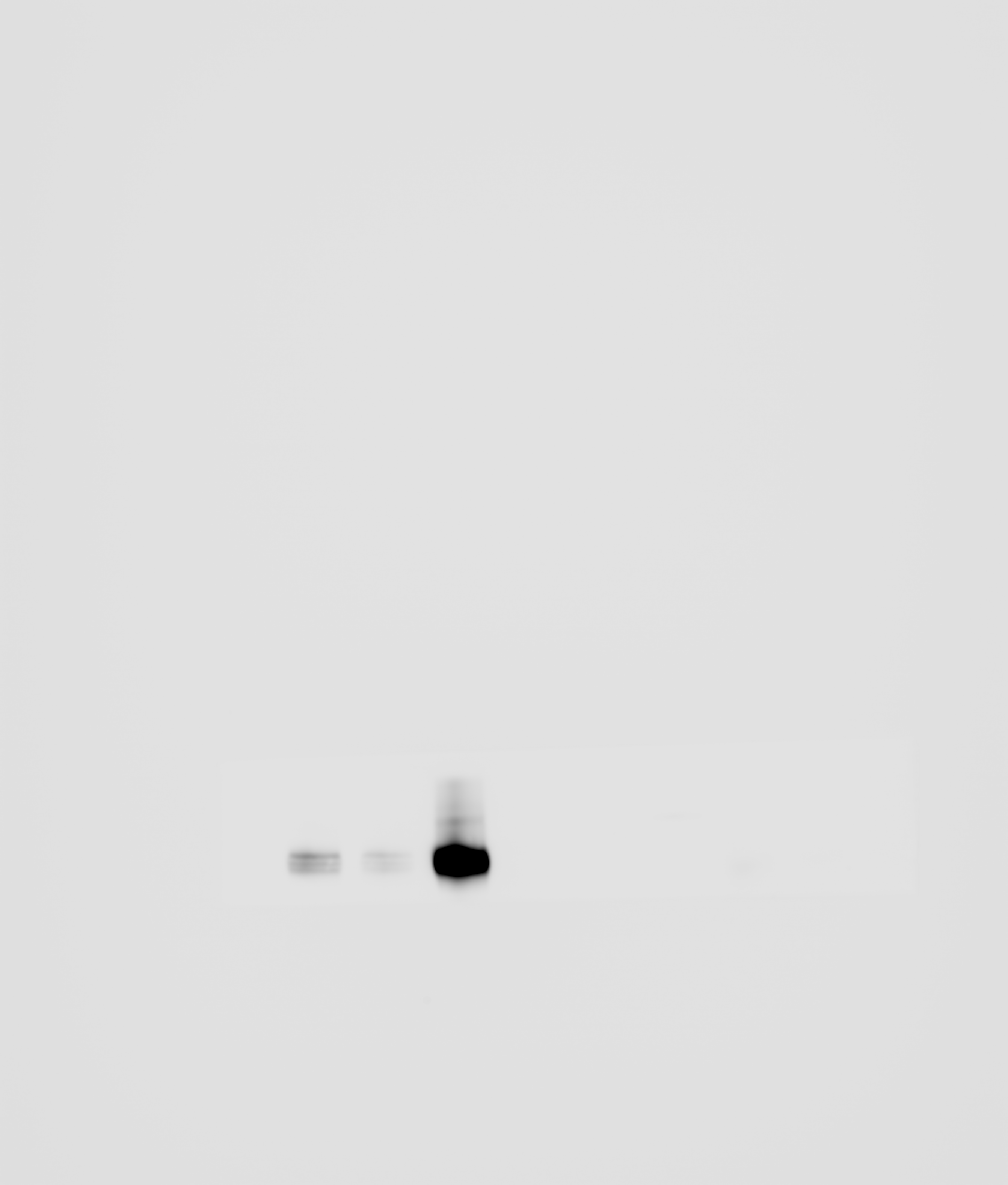

Supplement: Figure 4—source data 1. [file elife-86972-fig4-data1.zip › Figure 4C/HA-2S-sample.tif]

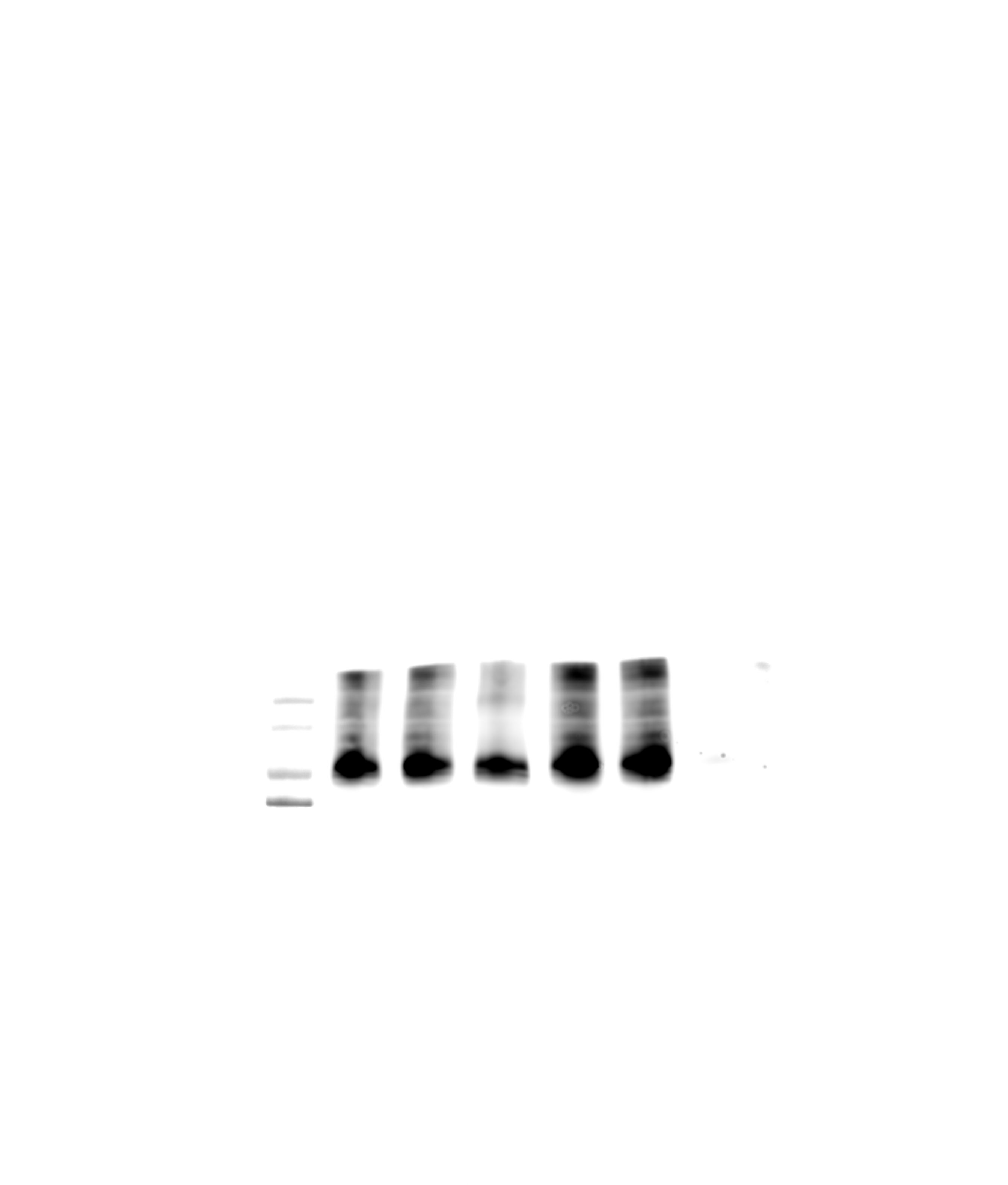

Supplement: Figure 4—source data 1. [file elife-86972-fig4-data1.zip › Figure 4C/SV2A.tif]

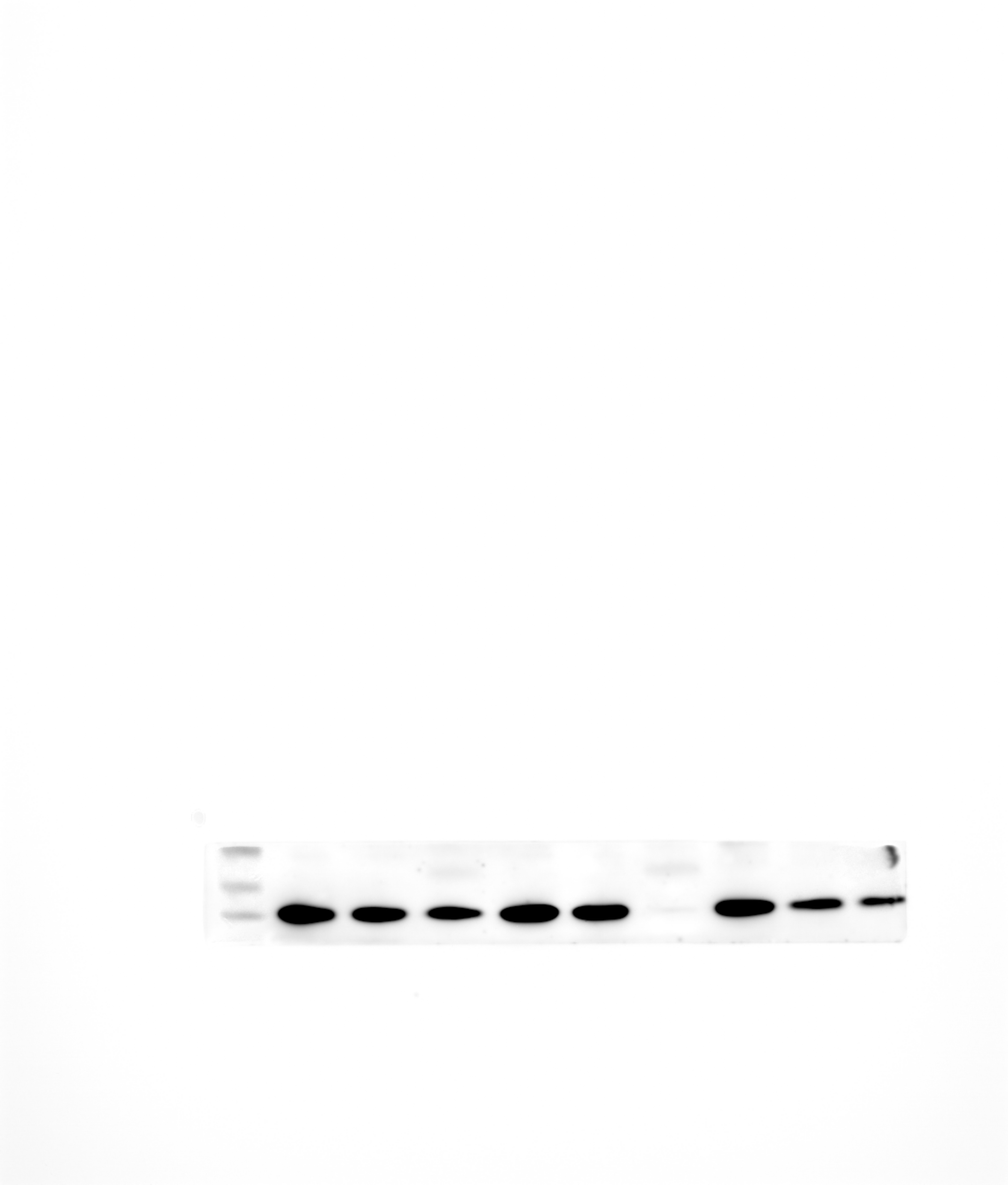

Supplement: Figure 4—source data 1. [file elife-86972-fig4-data1.zip › Figure 4C/Syb2.tif]

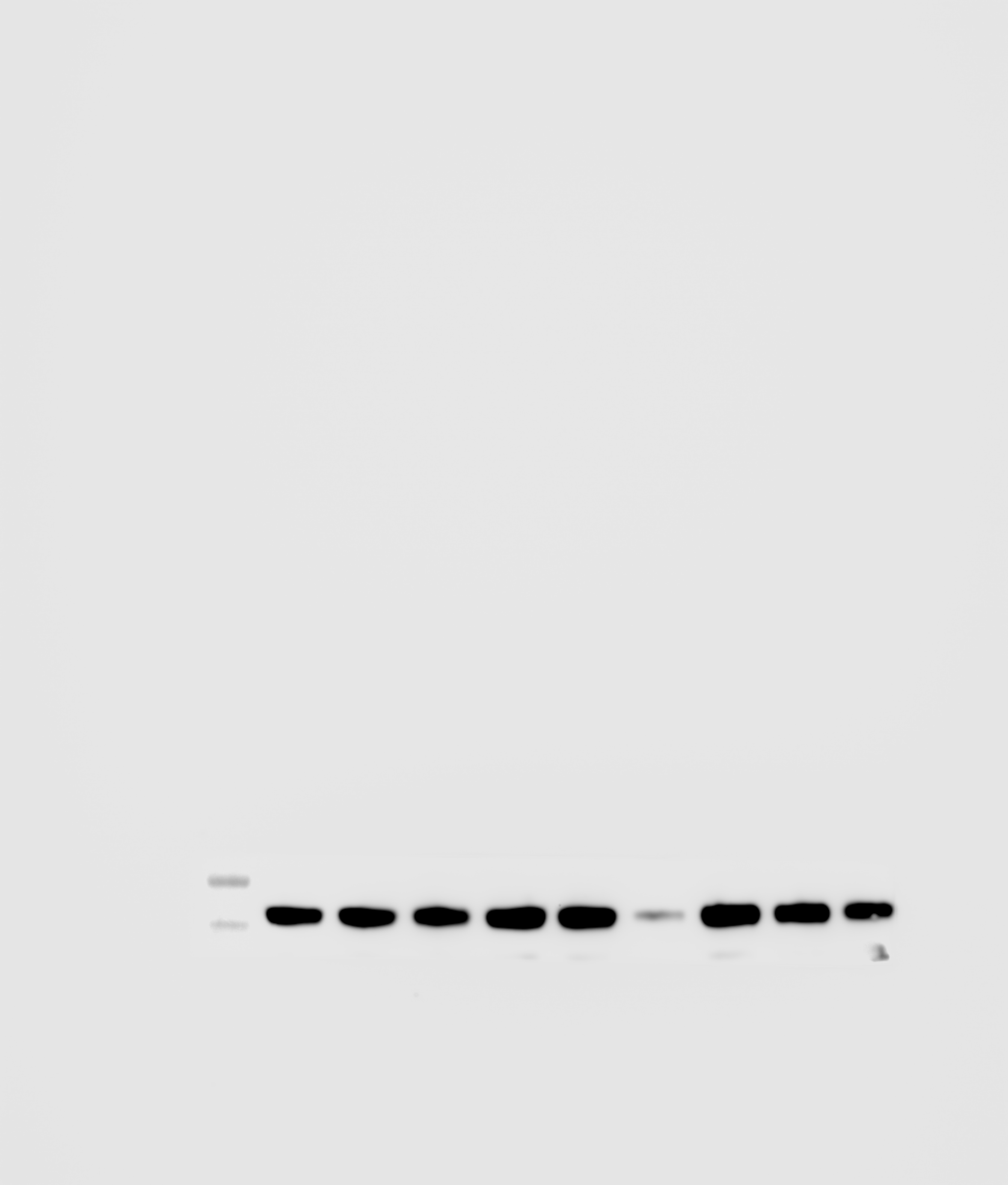

Supplement: Figure 4—source data 1. [file elife-86972-fig4-data1.zip › Figure 4C/Syp.tif]

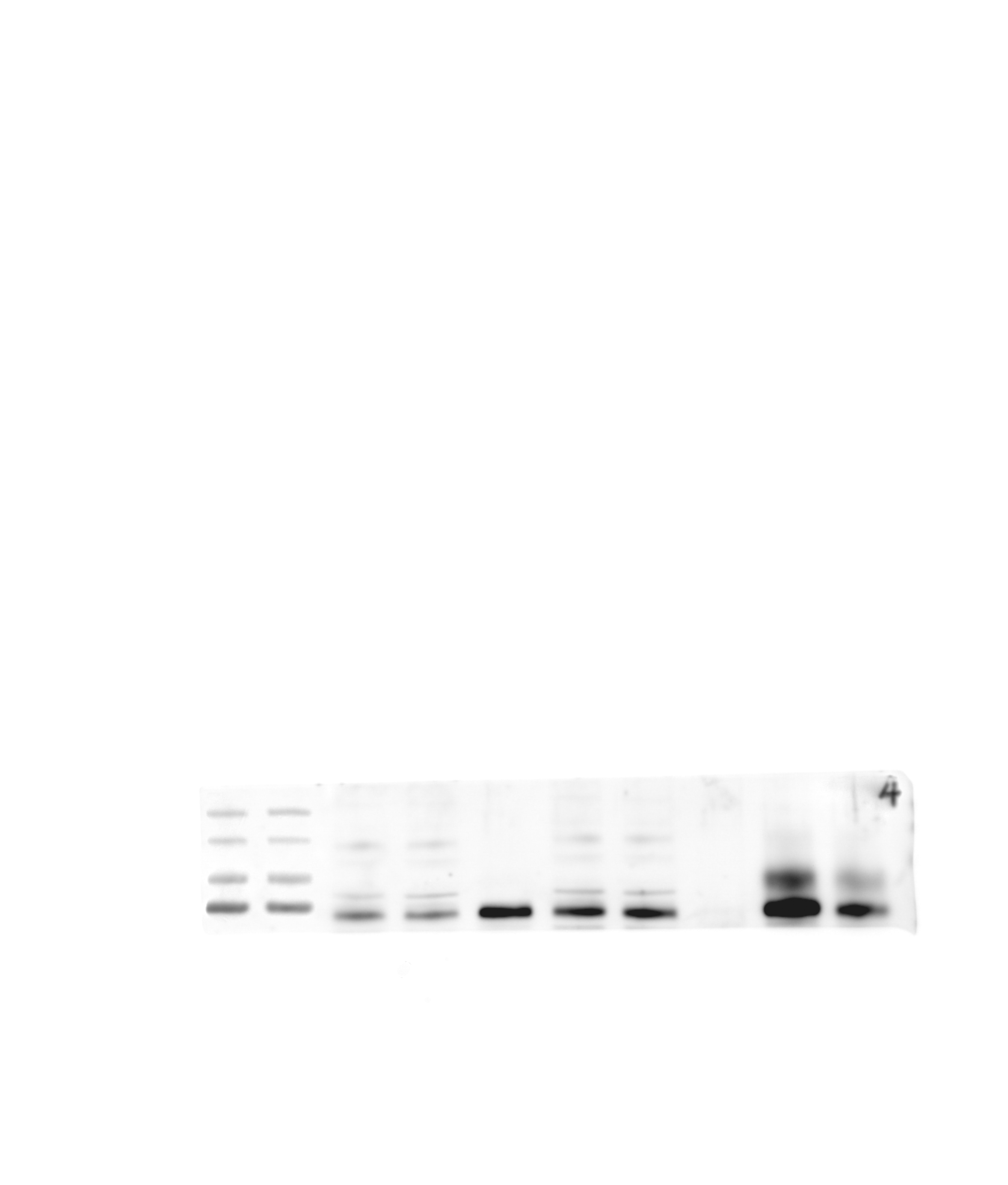

Supplement: Figure 4—source data 1. [file elife-86972-fig4-data1.zip › Figure 4C/Syt1.tif]

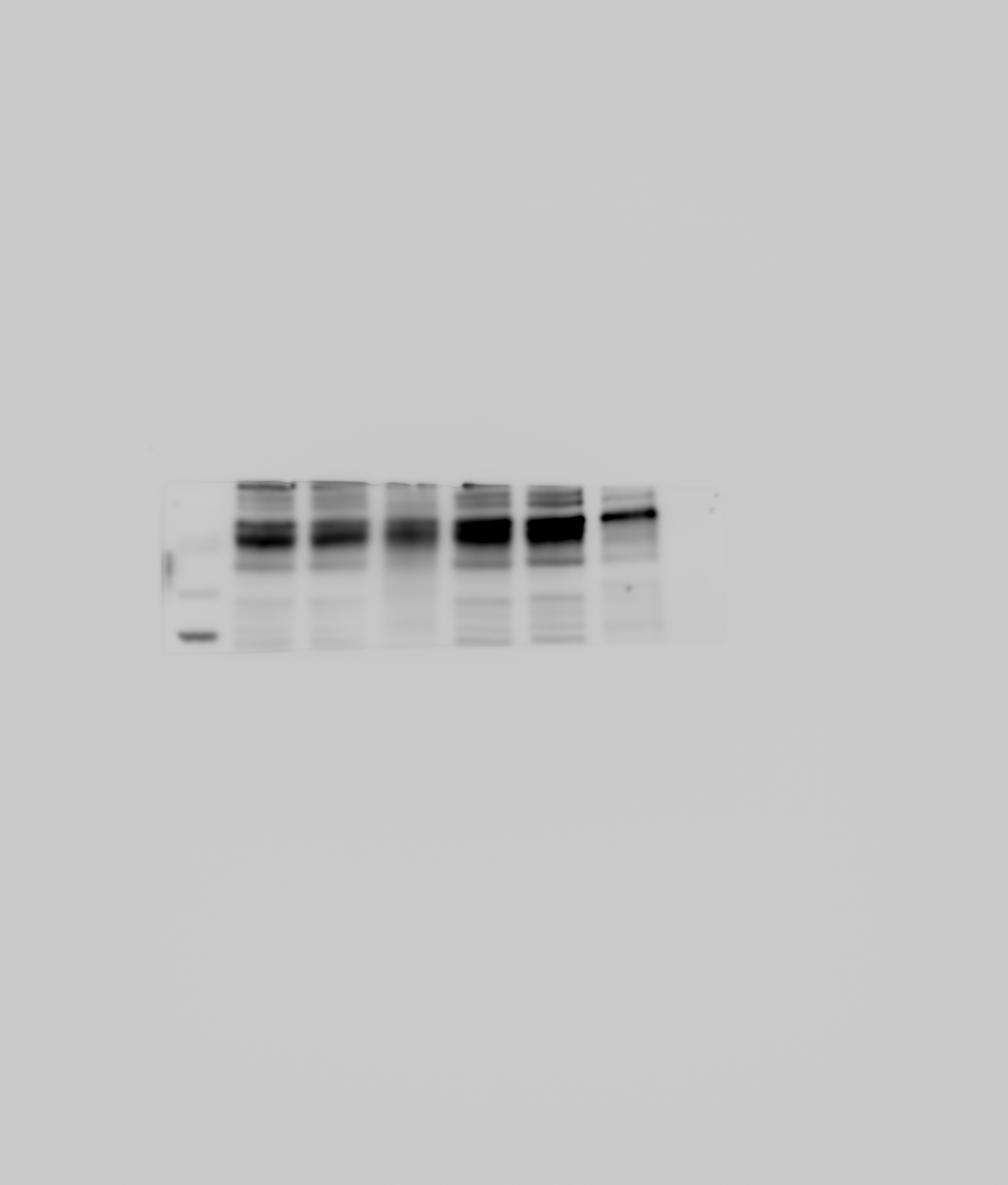

Supplement: Figure 4—source data 1. [file elife-86972-fig4-data1.zip › Figure 4C/VGAT.tif]

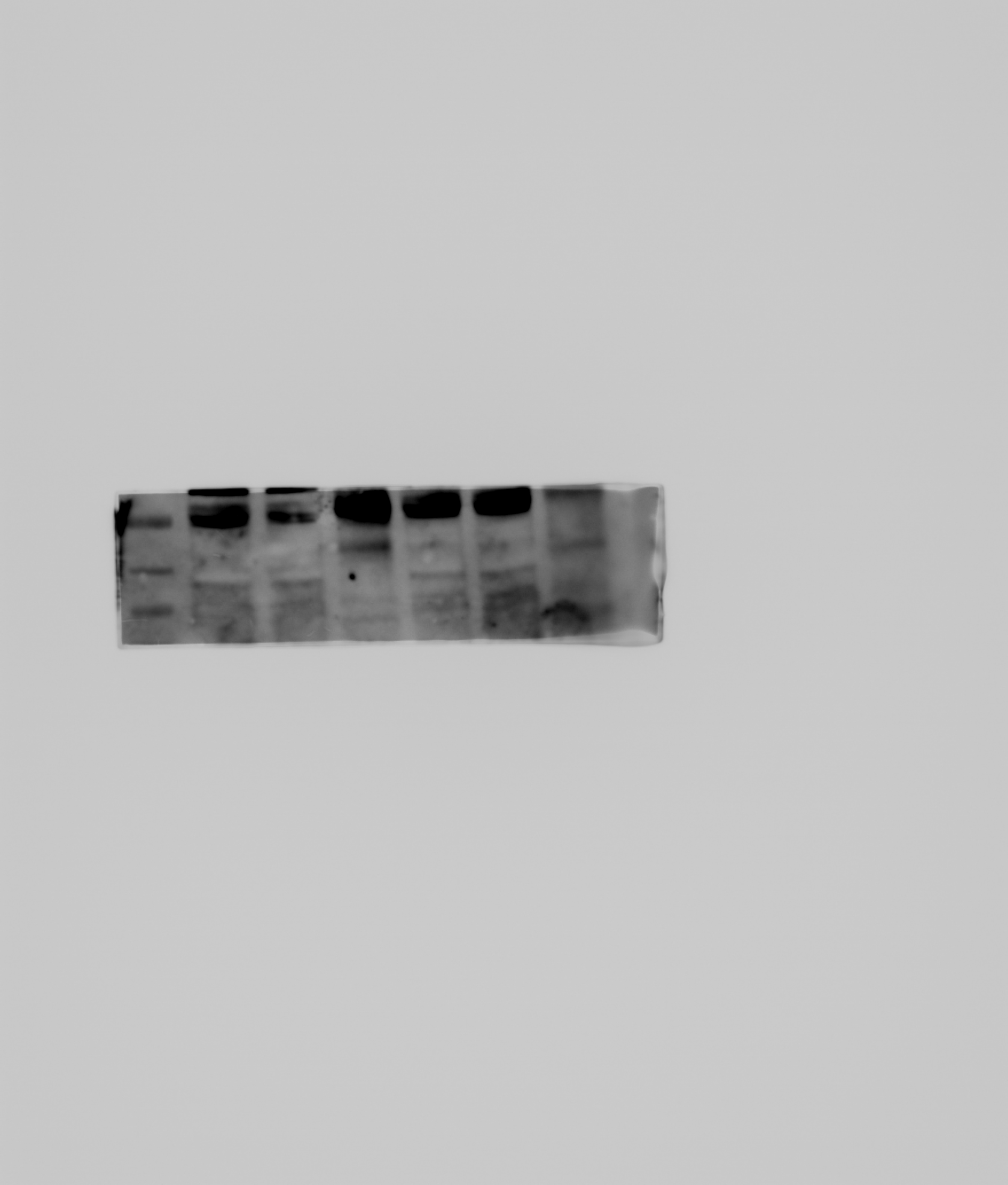

Supplement: Figure 4—source data 1. [file elife-86972-fig4-data1.zip › Figure 4C/VGLUT1-1.tif]

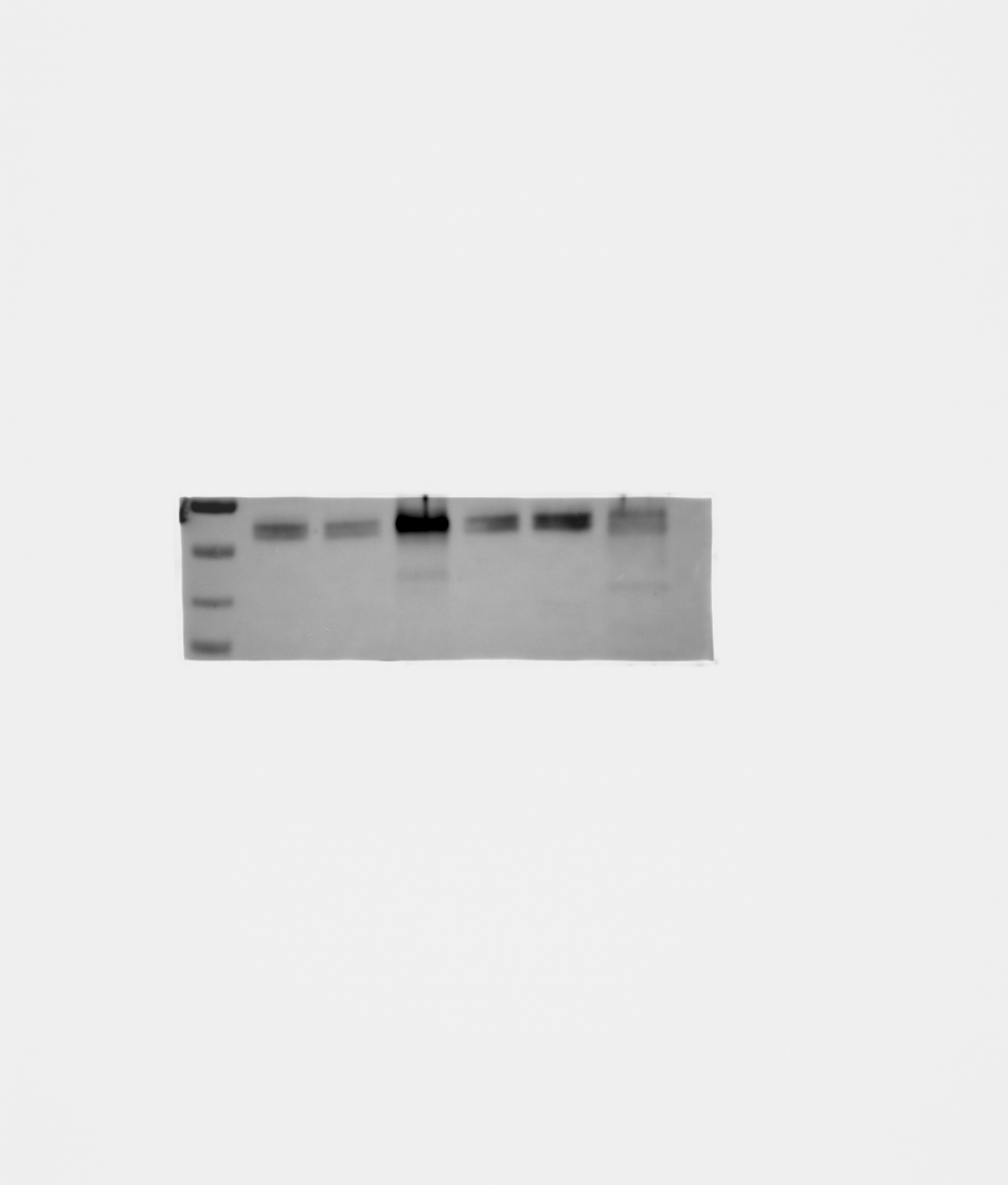

Supplement: Figure 4—source data 1. [file elife-86972-fig4-data1.zip › Figure 4C/VGLUT2-2.tif]

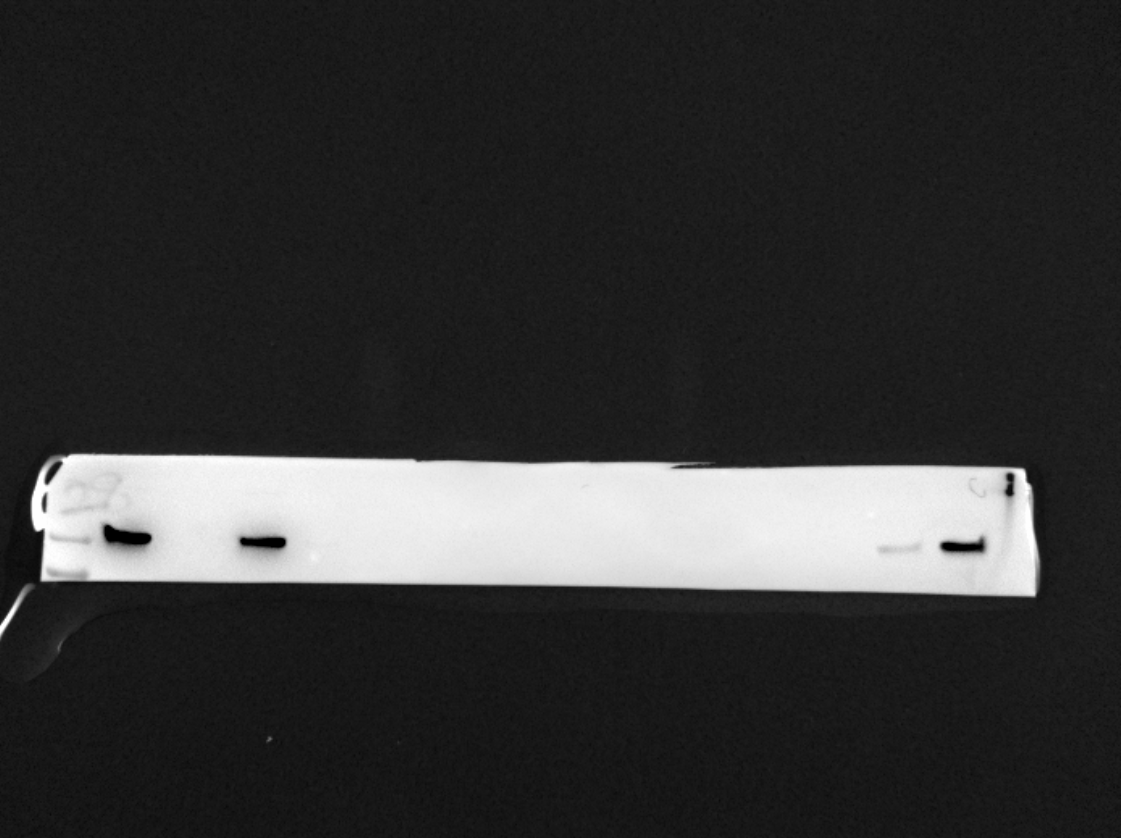

Supplement: Figure 4—source data 1. [file elife-86972-fig4-data1.zip › Figure 4D/SG-EEA1.tif]

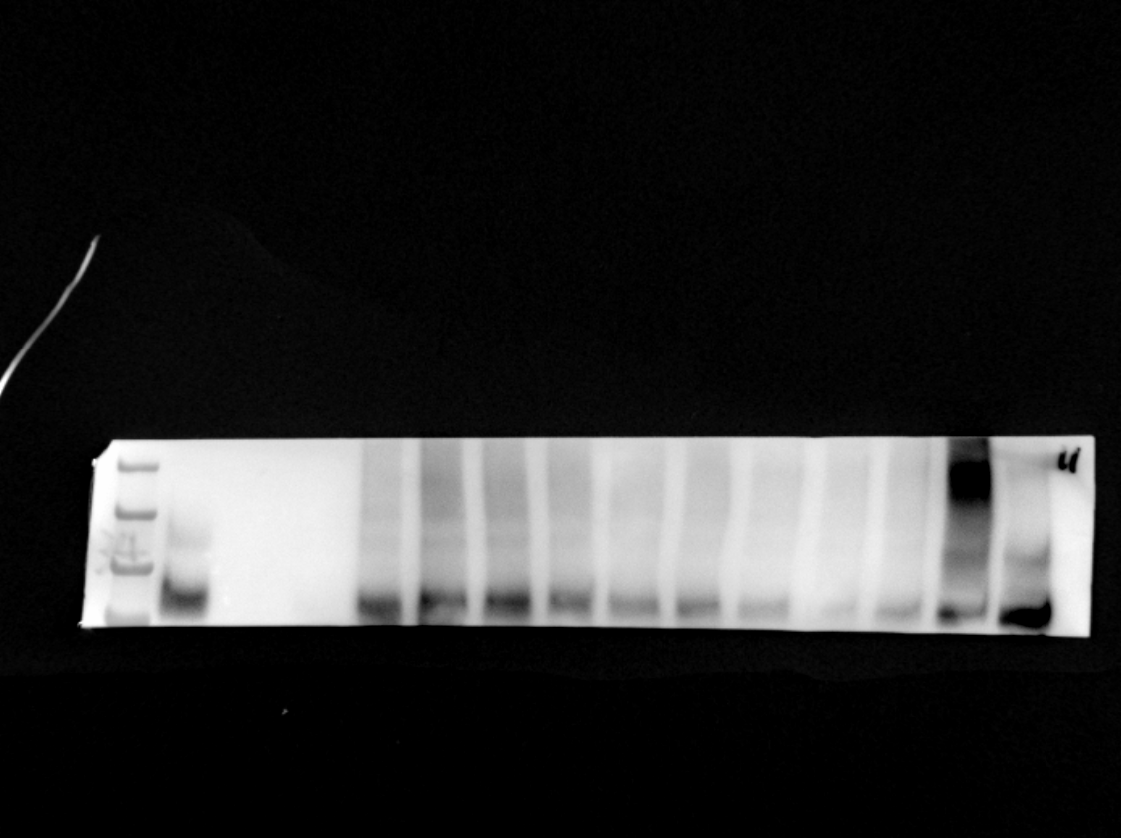

Supplement: Figure 4—source data 1. [file elife-86972-fig4-data1.zip › Figure 4D/SG-HA-2.tif]

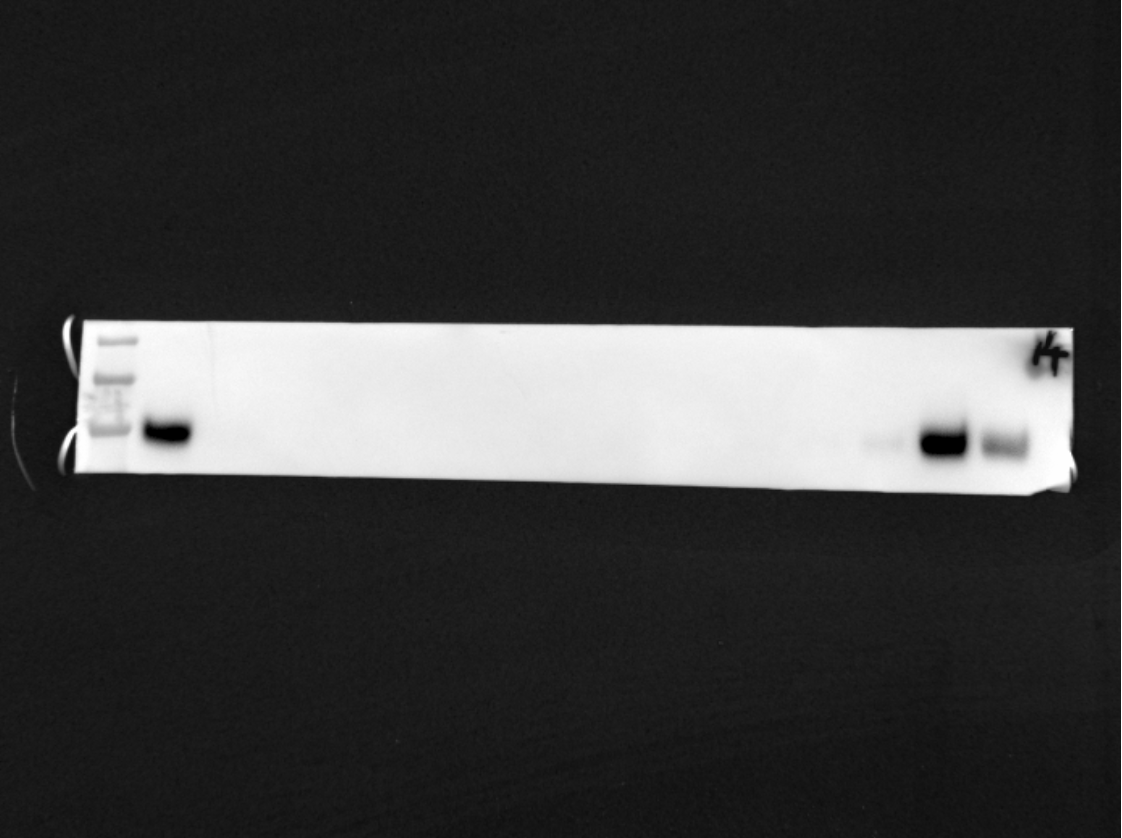

Supplement: Figure 4—source data 1. [file elife-86972-fig4-data1.zip › Figure 4D/SG-PSD95.tif]

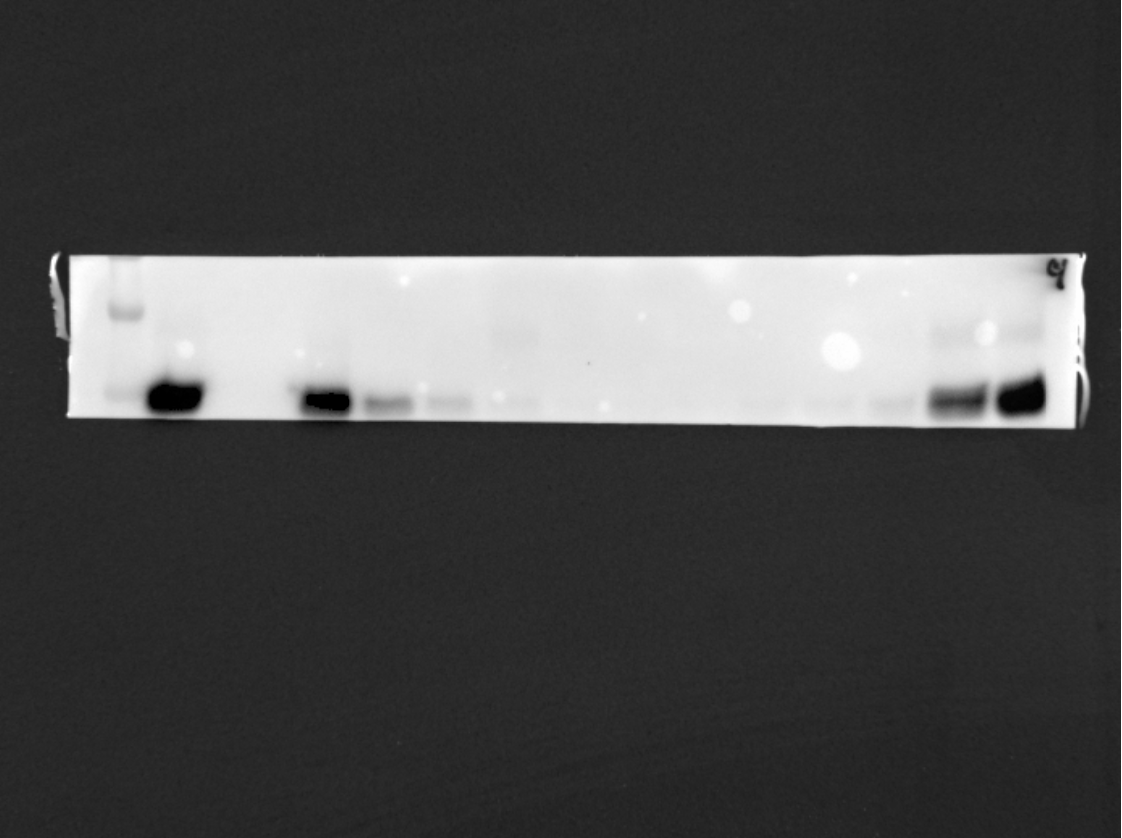

Supplement: Figure 4—source data 1. [file elife-86972-fig4-data1.zip › Figure 4D/SG-PSMC6.tif]

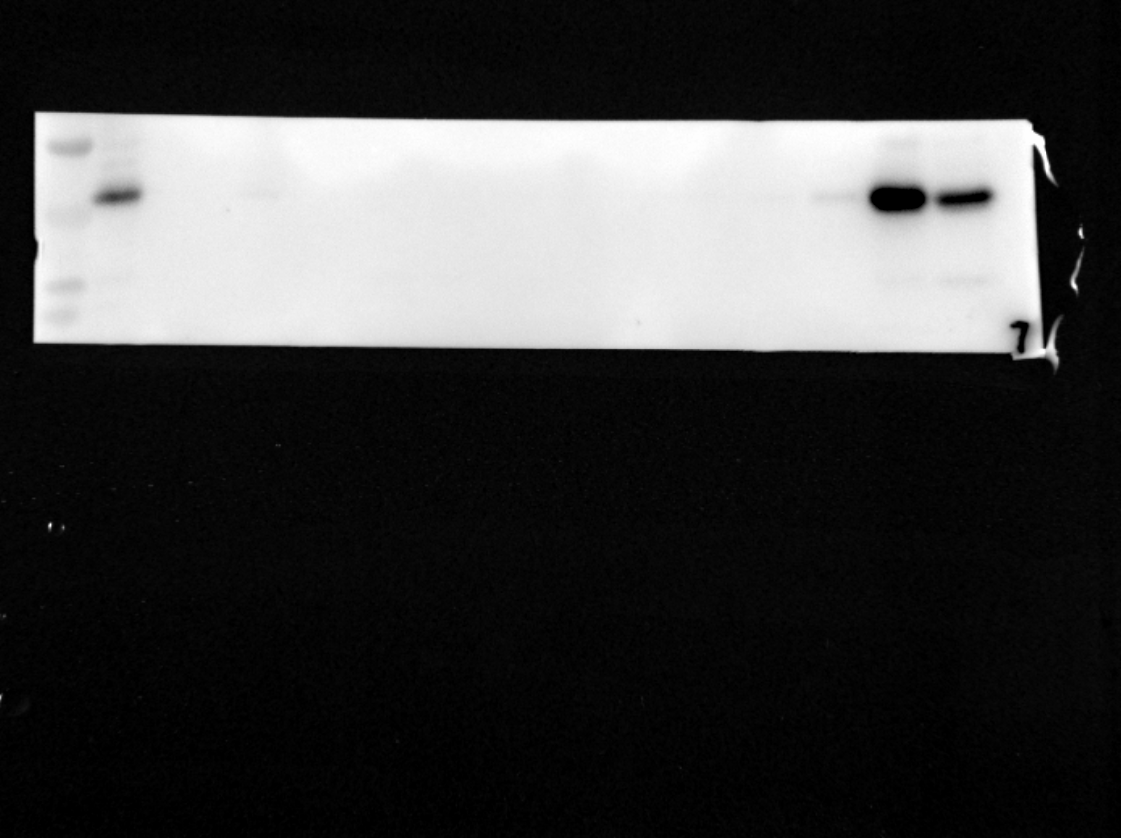

Supplement: Figure 4—source data 1. [file elife-86972-fig4-data1.zip › Figure 4D/SG-SNAP23.tif]

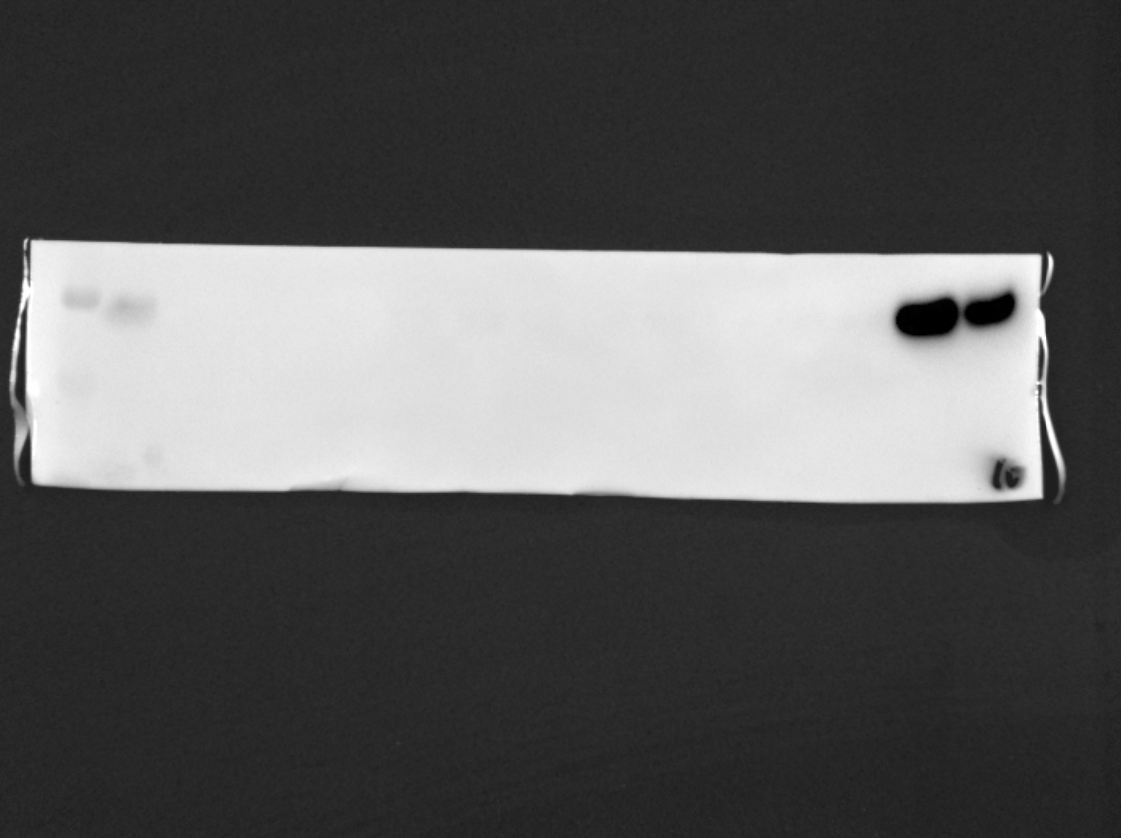

Supplement: Figure 4—source data 1. [file elife-86972-fig4-data1.zip › Figure 4D/SG-Synataxin6.tif]

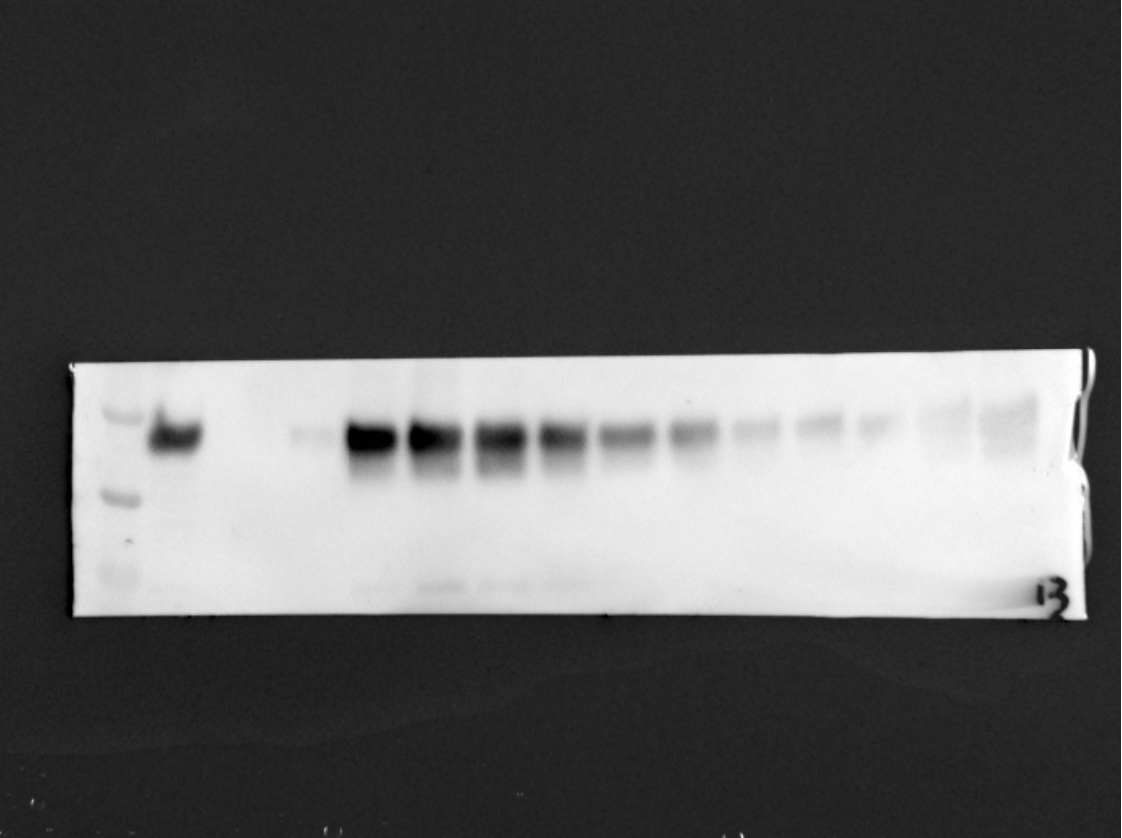

Supplement: Figure 4—source data 1. [file elife-86972-fig4-data1.zip › Figure 4D/SG-Syp-2.tif]

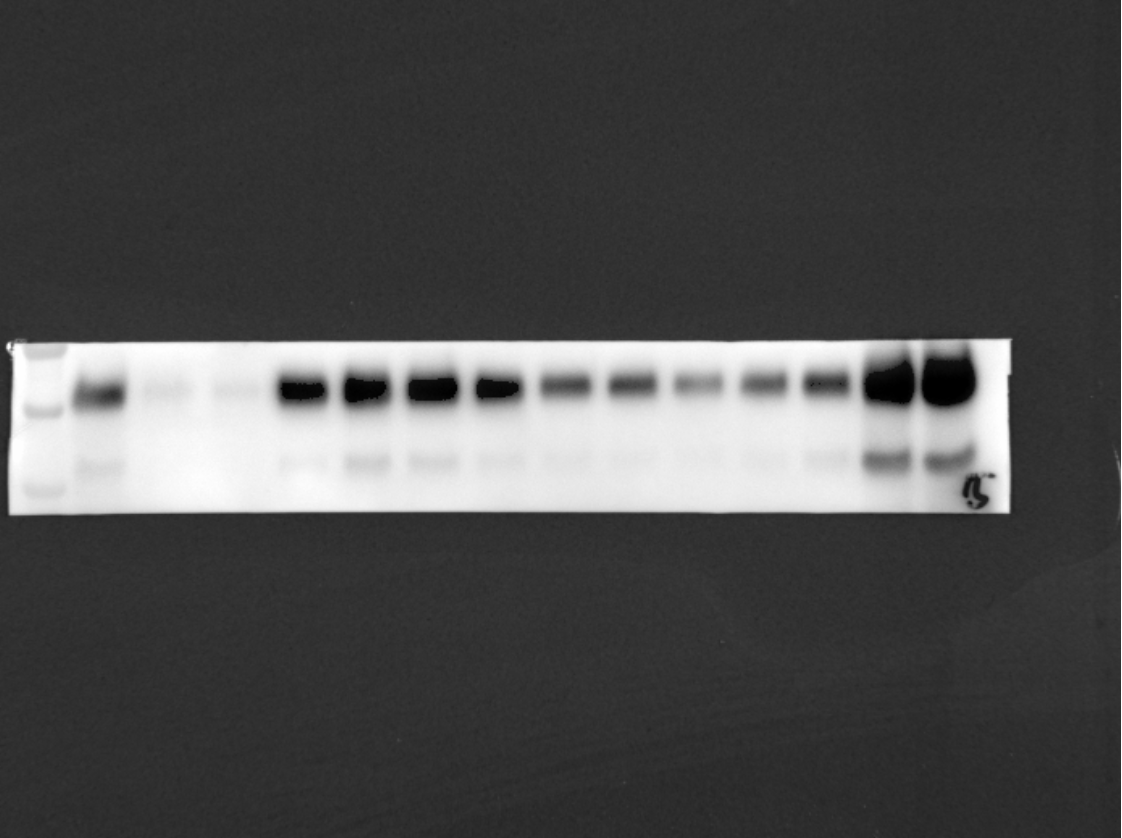

Supplement: Figure 4—source data 1. [file elife-86972-fig4-data1.zip › Figure 4D/SG-Syt1-2.tif]

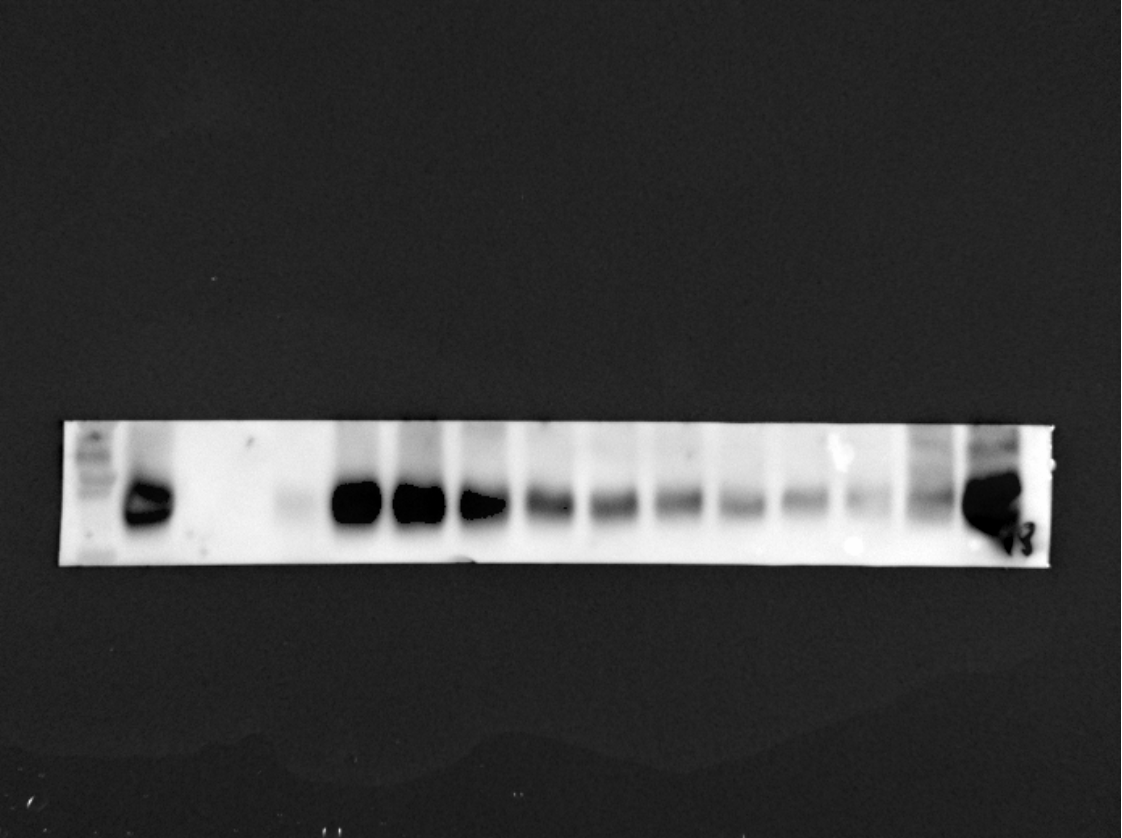

Supplement: Figure 4—source data 1. [file elife-86972-fig4-data1.zip › Figure 4D/SG-VGLUT1-2.tif]

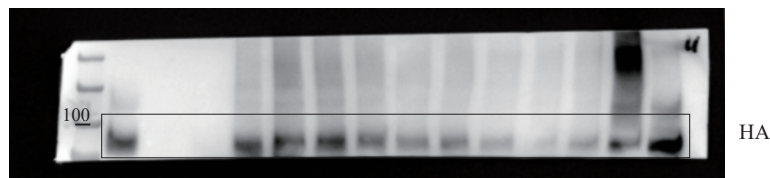

HA

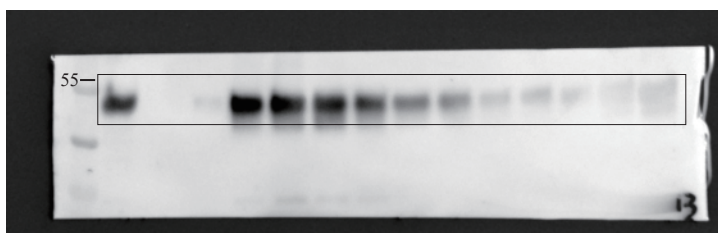

Syp

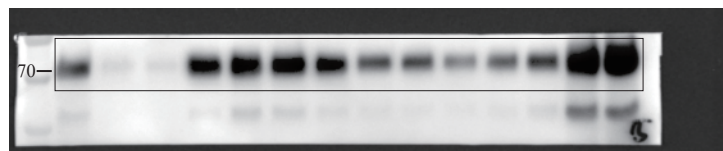

Syt1

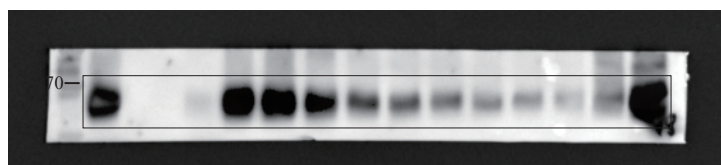

VGlut1

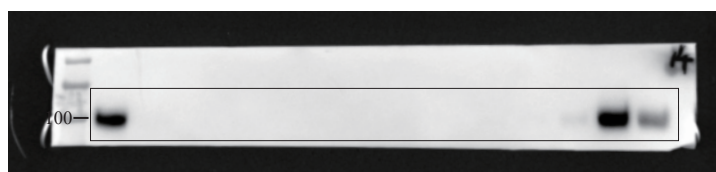

PSD95

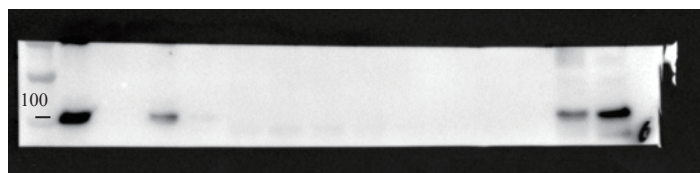

ERp72

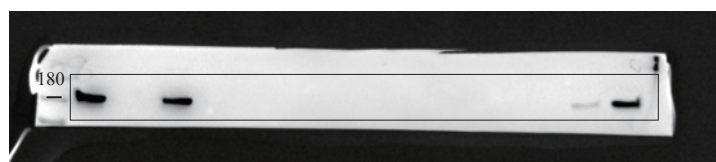

EEA1

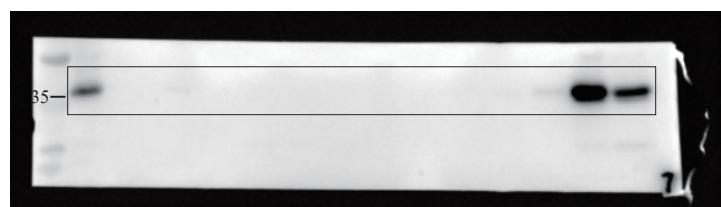

SNAP23

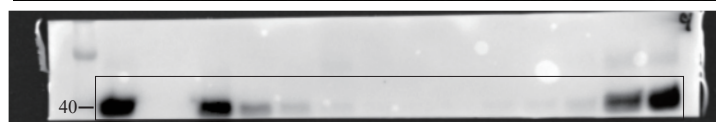

PSMC6

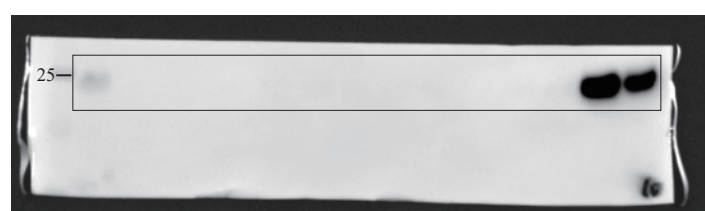

Syntaxin6

Supplement: Figure 4—source data 2. [file elife-86972-fig4-data2.zip › Figure4D-Source Data-WB.pdf]

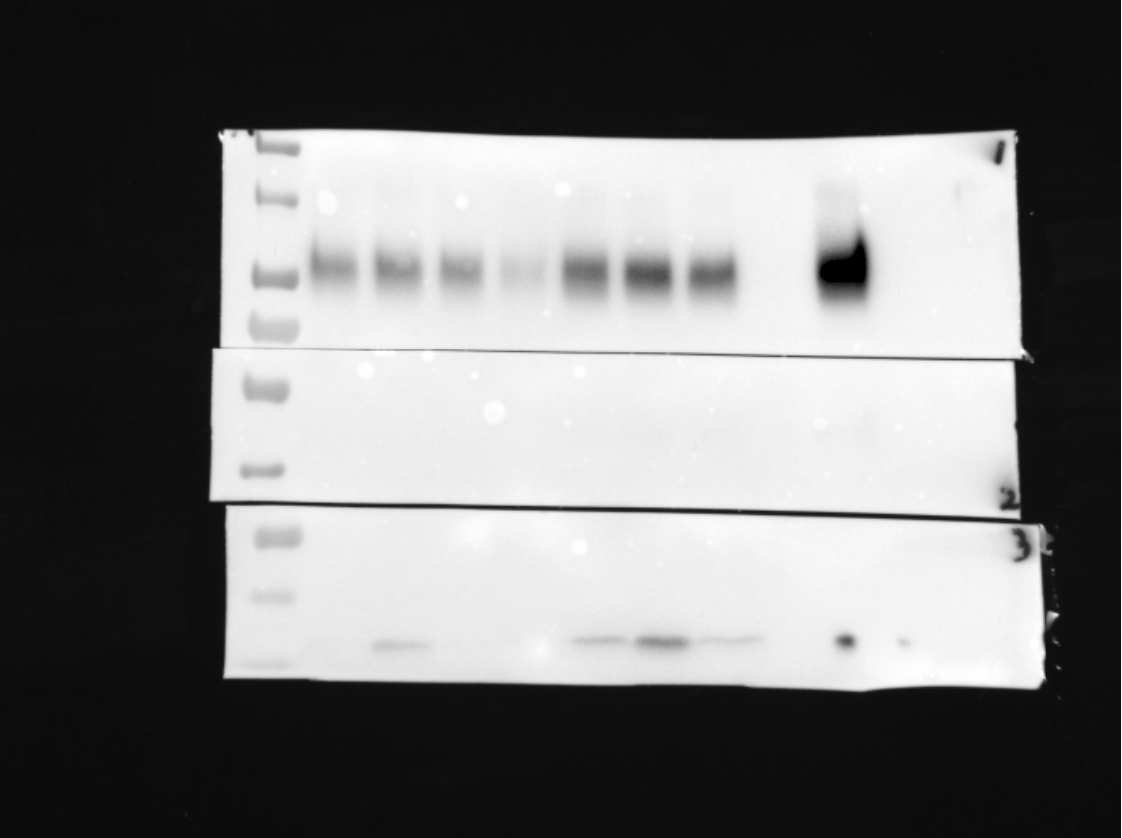

Supplement: Figure 4—figure supplement 1—source data 1. [file elife-86972-fig4-figsupp1-data1.zip › Figure 4-S1C/HA.tif]

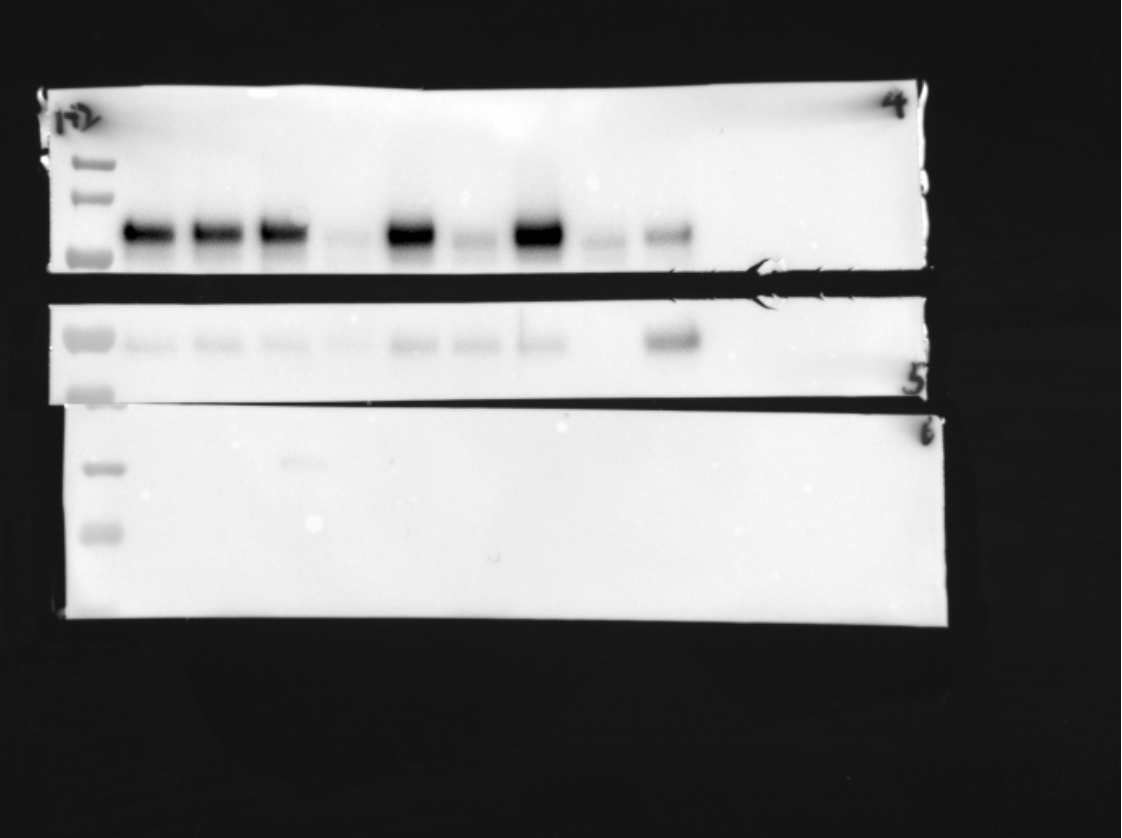

Supplement: Figure 4—figure supplement 1—source data 1. [file elife-86972-fig4-figsupp1-data1.zip › Figure 4-S1C/PSD95-1.tif]

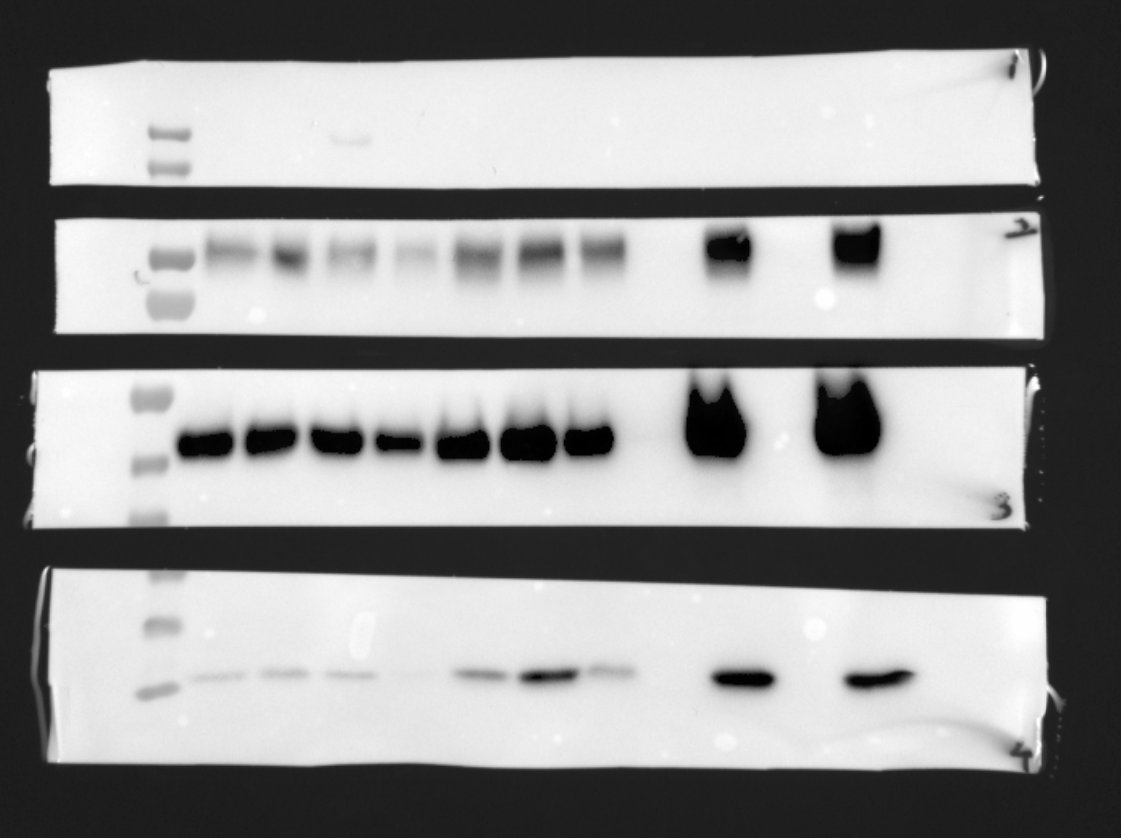

Supplement: Figure 4—figure supplement 1—source data 1. [file elife-86972-fig4-figsupp1-data1.zip › Figure 4-S1C/Syb2.tif]

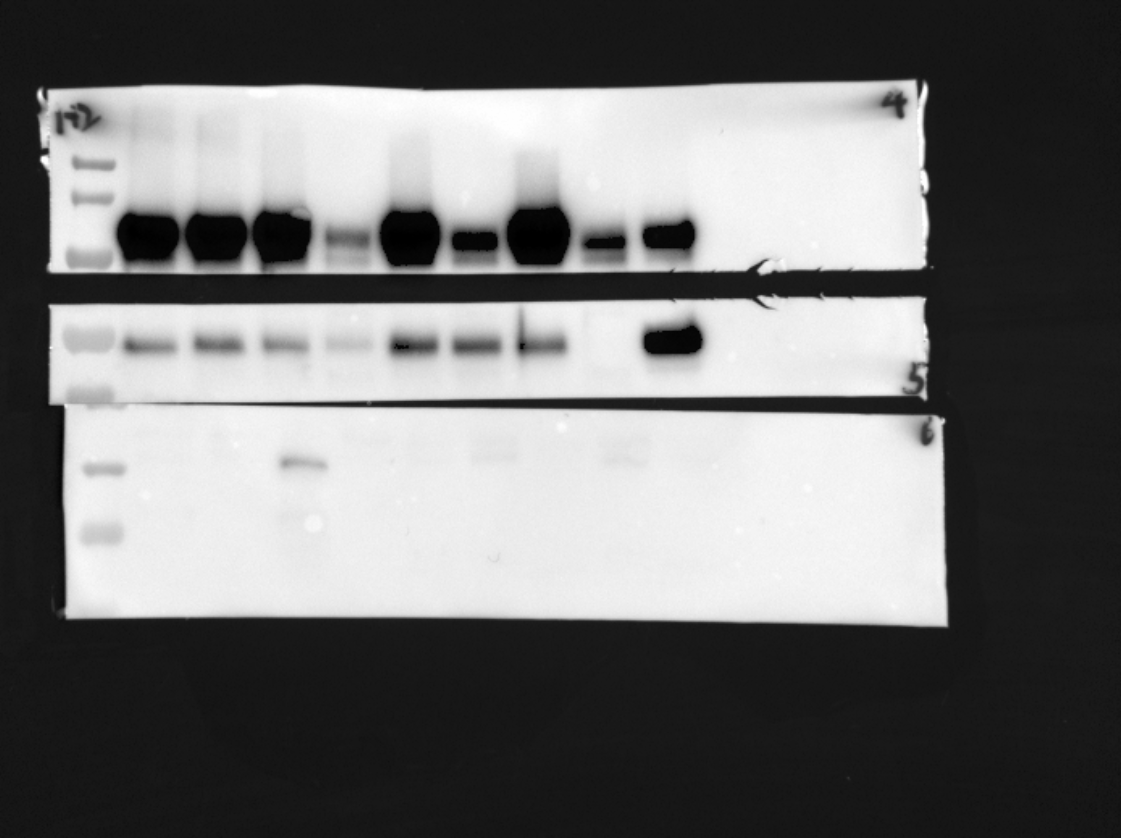

Supplement: Figure 4—figure supplement 1—source data 1. [file elife-86972-fig4-figsupp1-data1.zip › Figure 4-S1C/Syt1-2.tif]

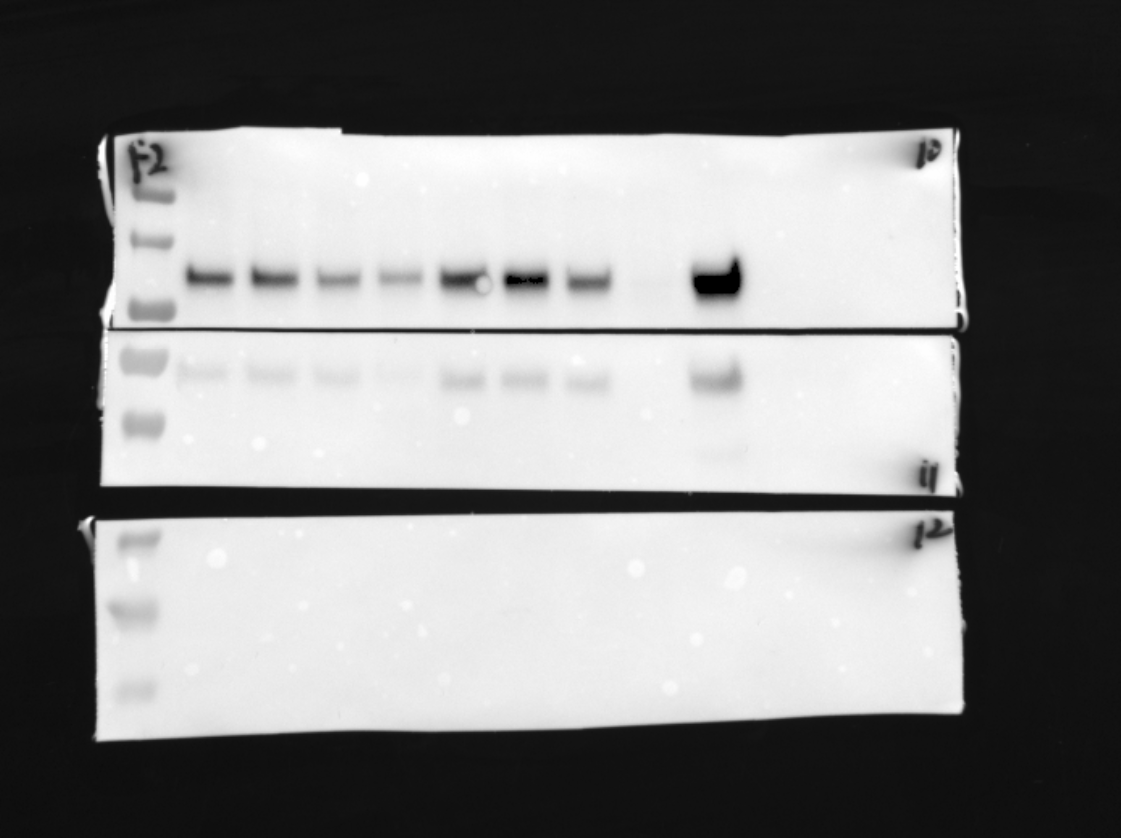

Supplement: Figure 4—figure supplement 1—source data 1. [file elife-86972-fig4-figsupp1-data1.zip › Figure 4-S1C/V-ATPase1.tif]

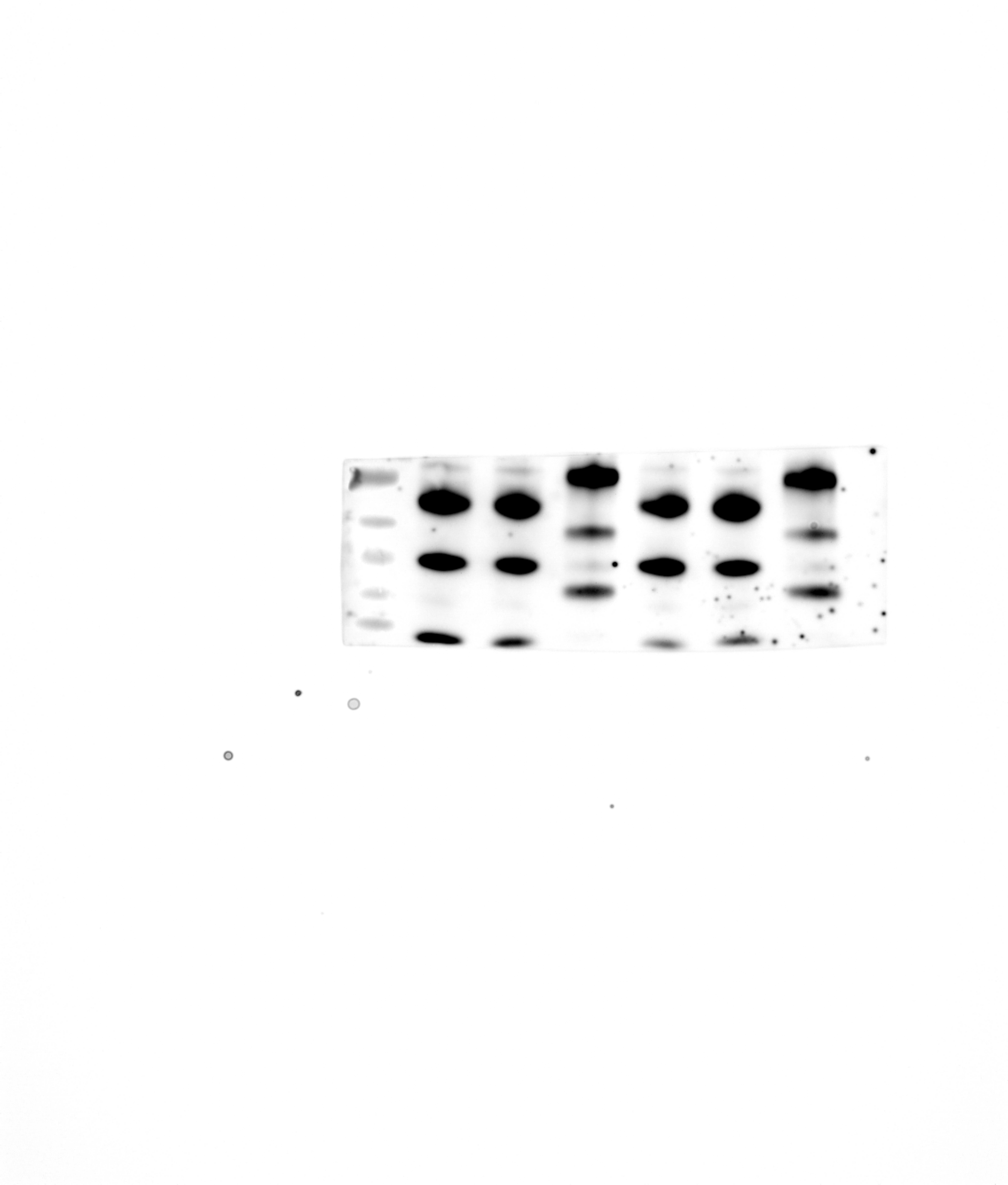

Supplement: Figure 4—figure supplement 1—source data 1. [file elife-86972-fig4-figsupp1-data1.zip › Figure 4-S1D/Cathepsin D.tif]

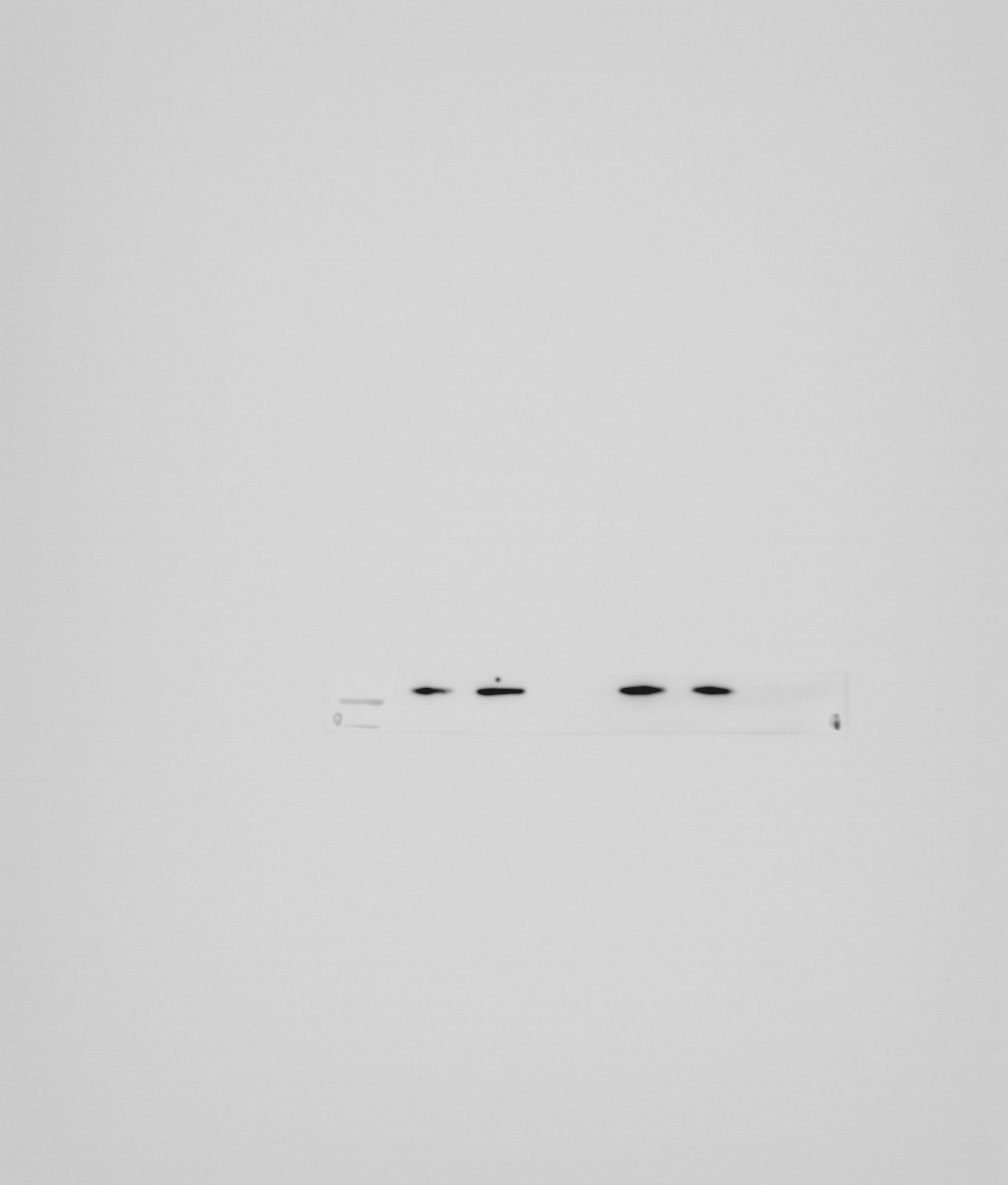

Supplement: Figure 4—figure supplement 1—source data 1. [file elife-86972-fig4-figsupp1-data1.zip › Figure 4-S1D/EEA1.tif]

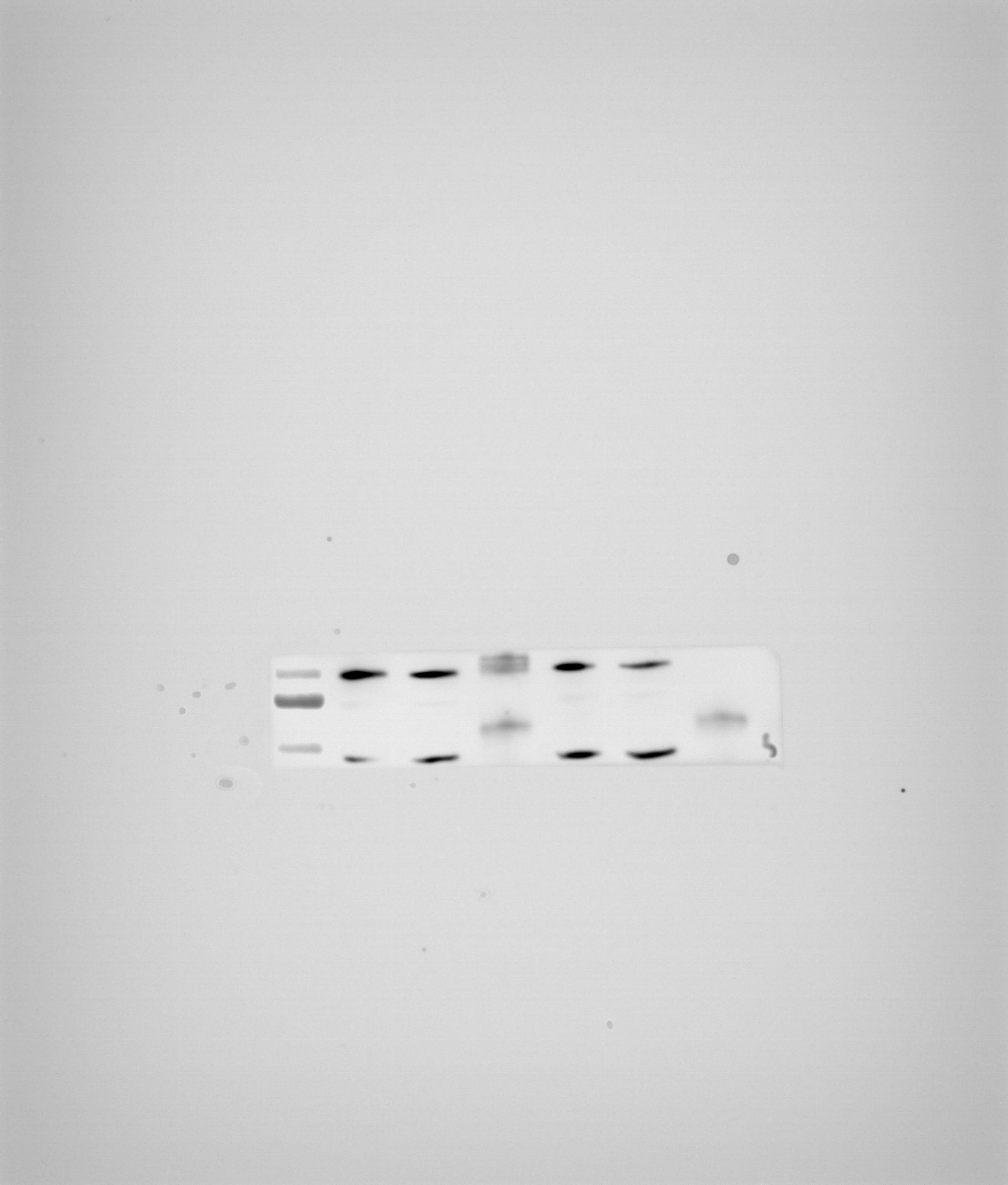

Supplement: Figure 4—figure supplement 1—source data 1. [file elife-86972-fig4-figsupp1-data1.zip › Figure 4-S1D/EPR72.tif]

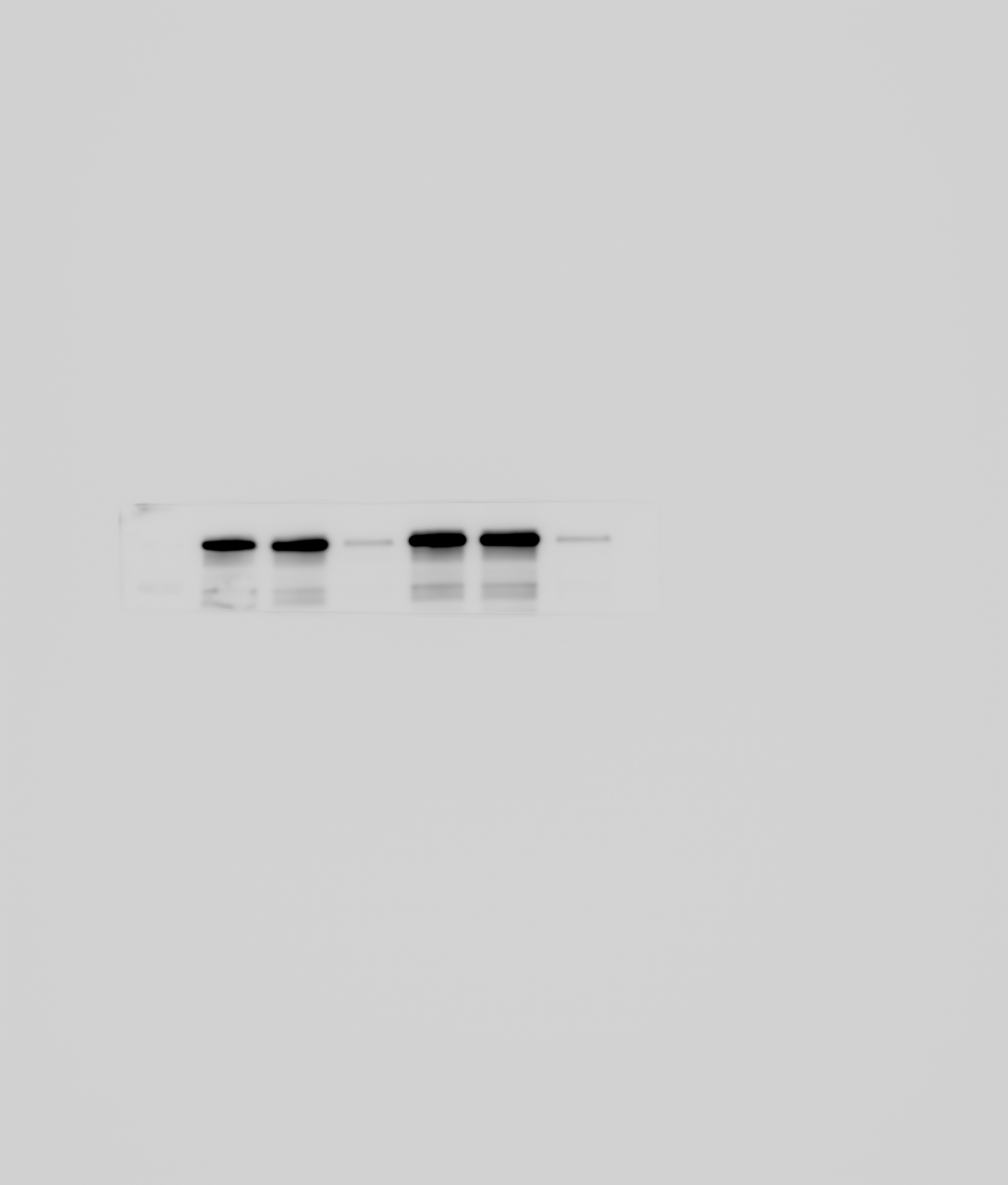

Supplement: Figure 4—figure supplement 1—source data 1. [file elife-86972-fig4-figsupp1-data1.zip › Figure 4-S1D/ERC1b-2-1.tif]

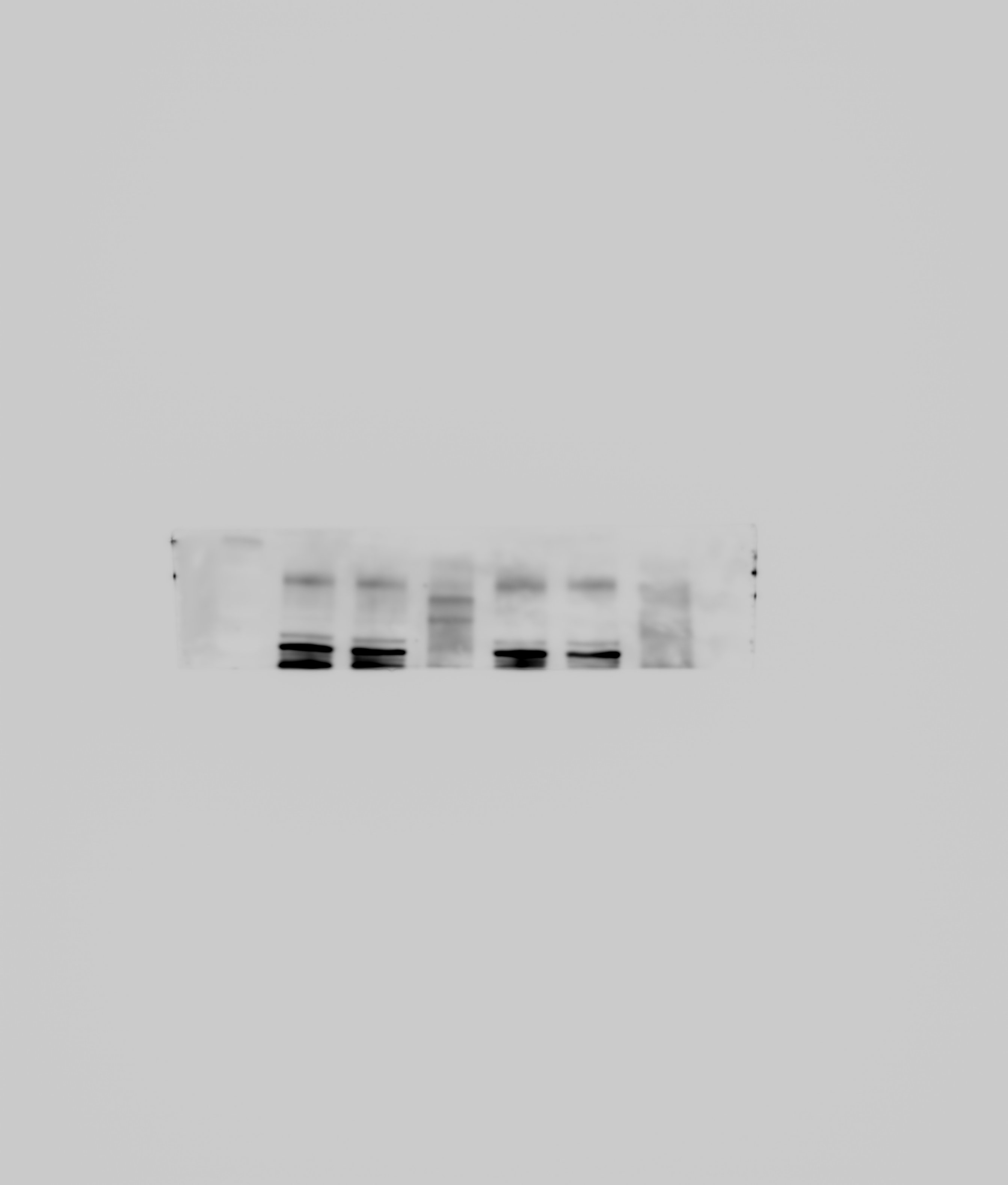

Supplement: Figure 4—figure supplement 1—source data 1. [file elife-86972-fig4-figsupp1-data1.zip › Figure 4-S1D/GluN1-1s.tif]

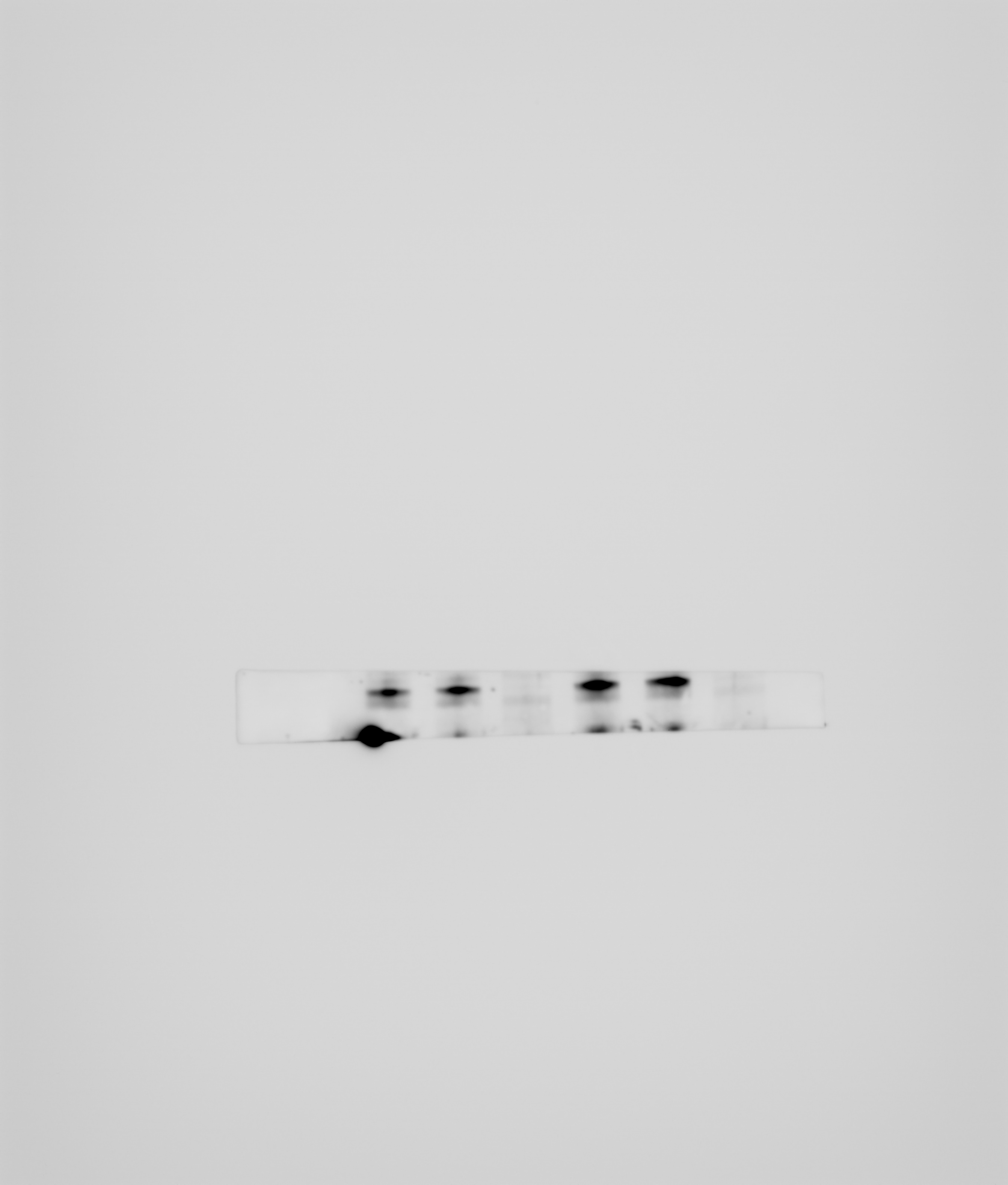

Supplement: Figure 4—figure supplement 1—source data 1. [file elife-86972-fig4-figsupp1-data1.zip › Figure 4-S1D/GM130-sample.tif]

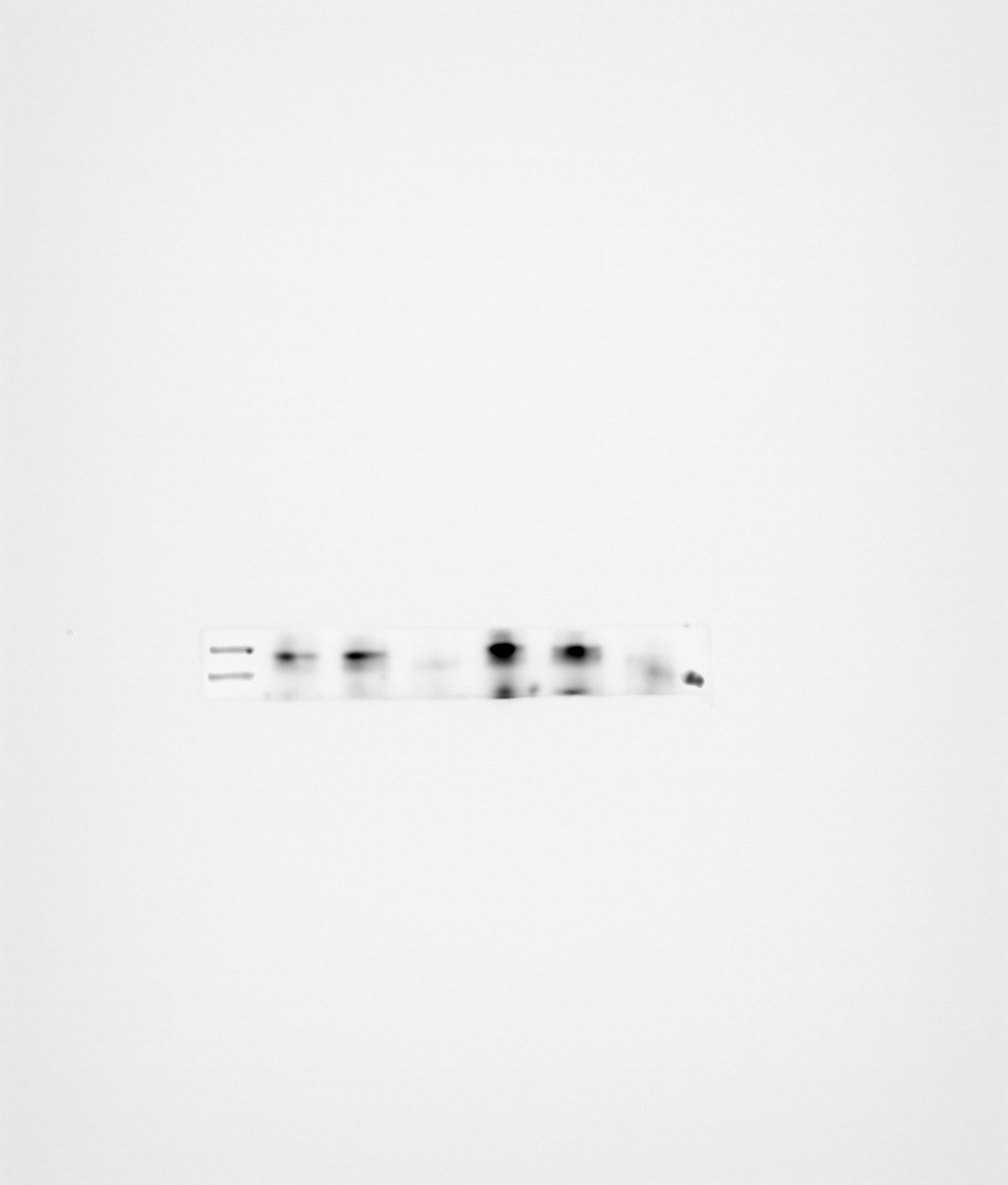

Supplement: Figure 4—figure supplement 1—source data 1. [file elife-86972-fig4-figsupp1-data1.zip › Figure 4-S1D/GM130.tif]

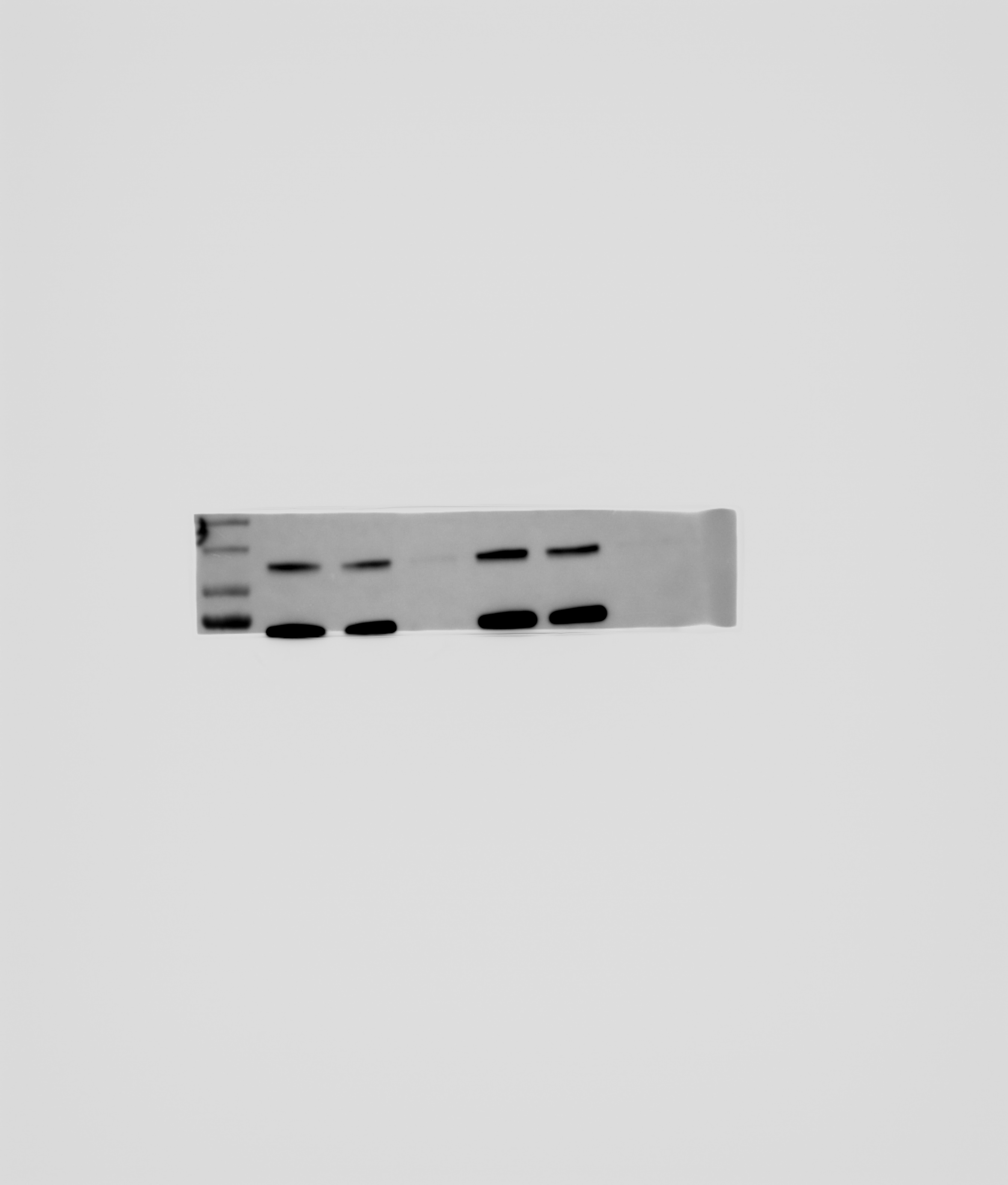

Supplement: Figure 4—figure supplement 1—source data 1. [file elife-86972-fig4-figsupp1-data1.zip › Figure 4-S1D/Goglin-97-2.tif]

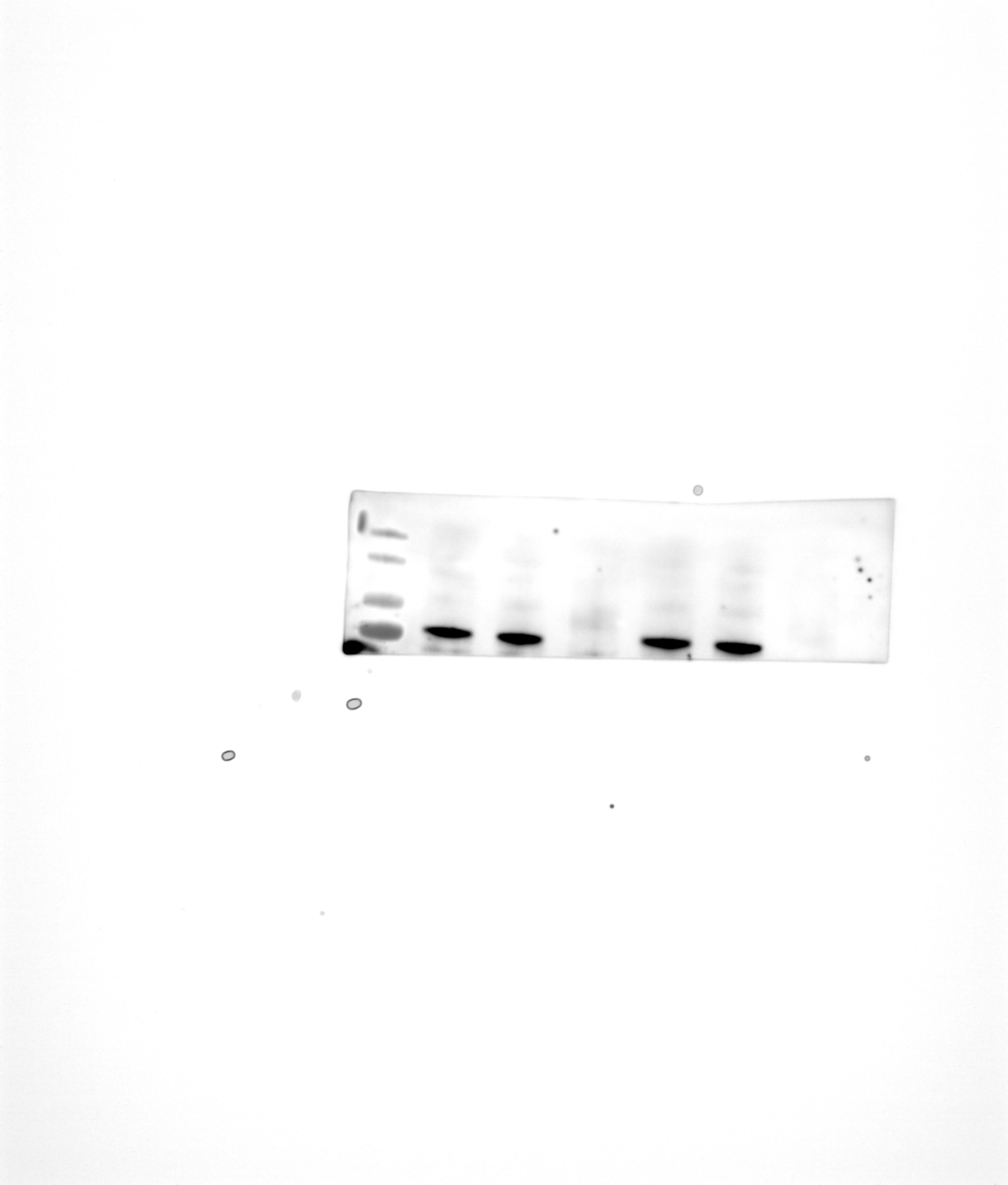

Supplement: Figure 4—figure supplement 1—source data 1. [file elife-86972-fig4-figsupp1-data1.zip › Figure 4-S1D/LAMP2 (2).tif]

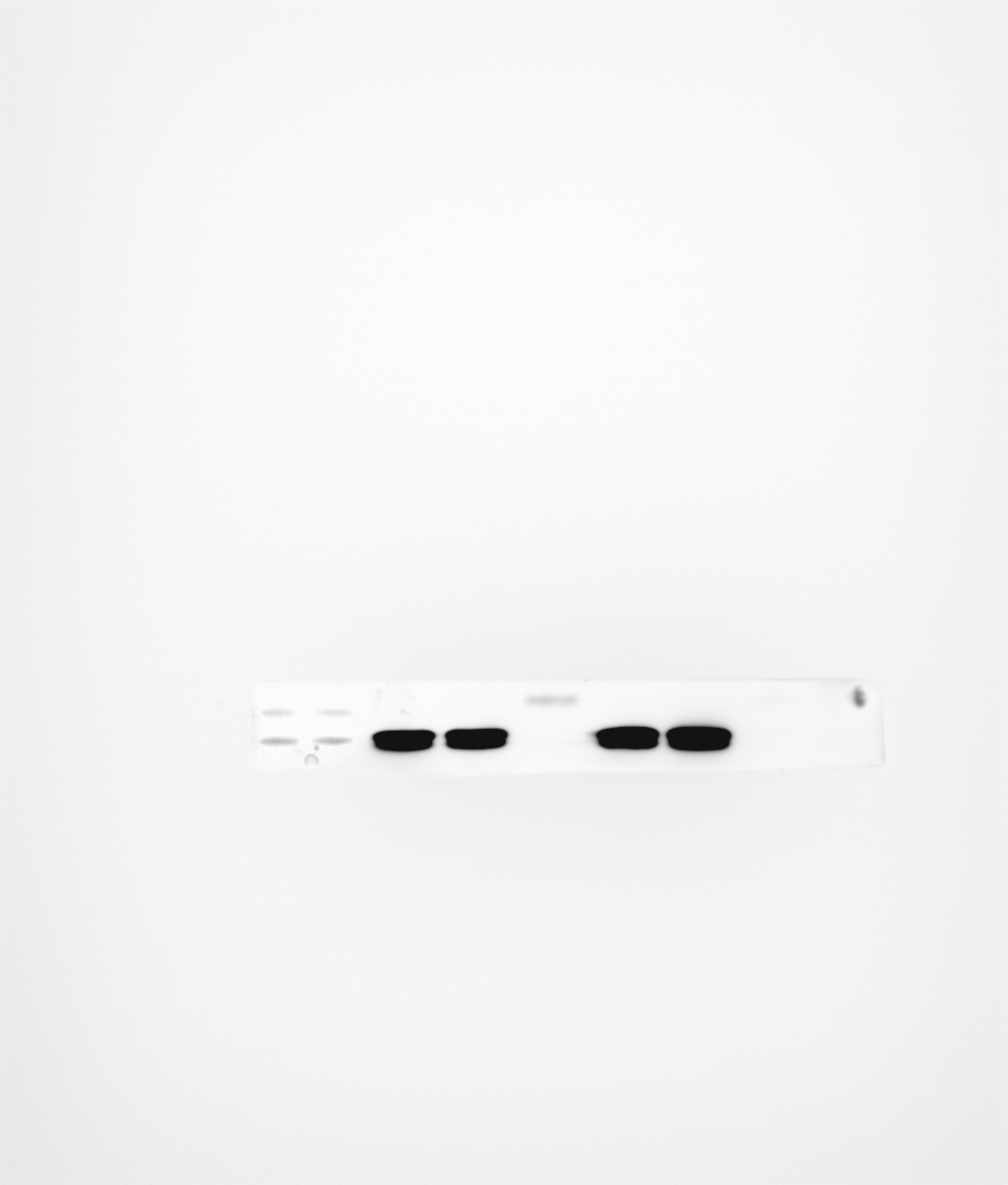

Supplement: Figure 4—figure supplement 1—source data 1. [file elife-86972-fig4-figsupp1-data1.zip › Figure 4-S1D/LC3B.tif]

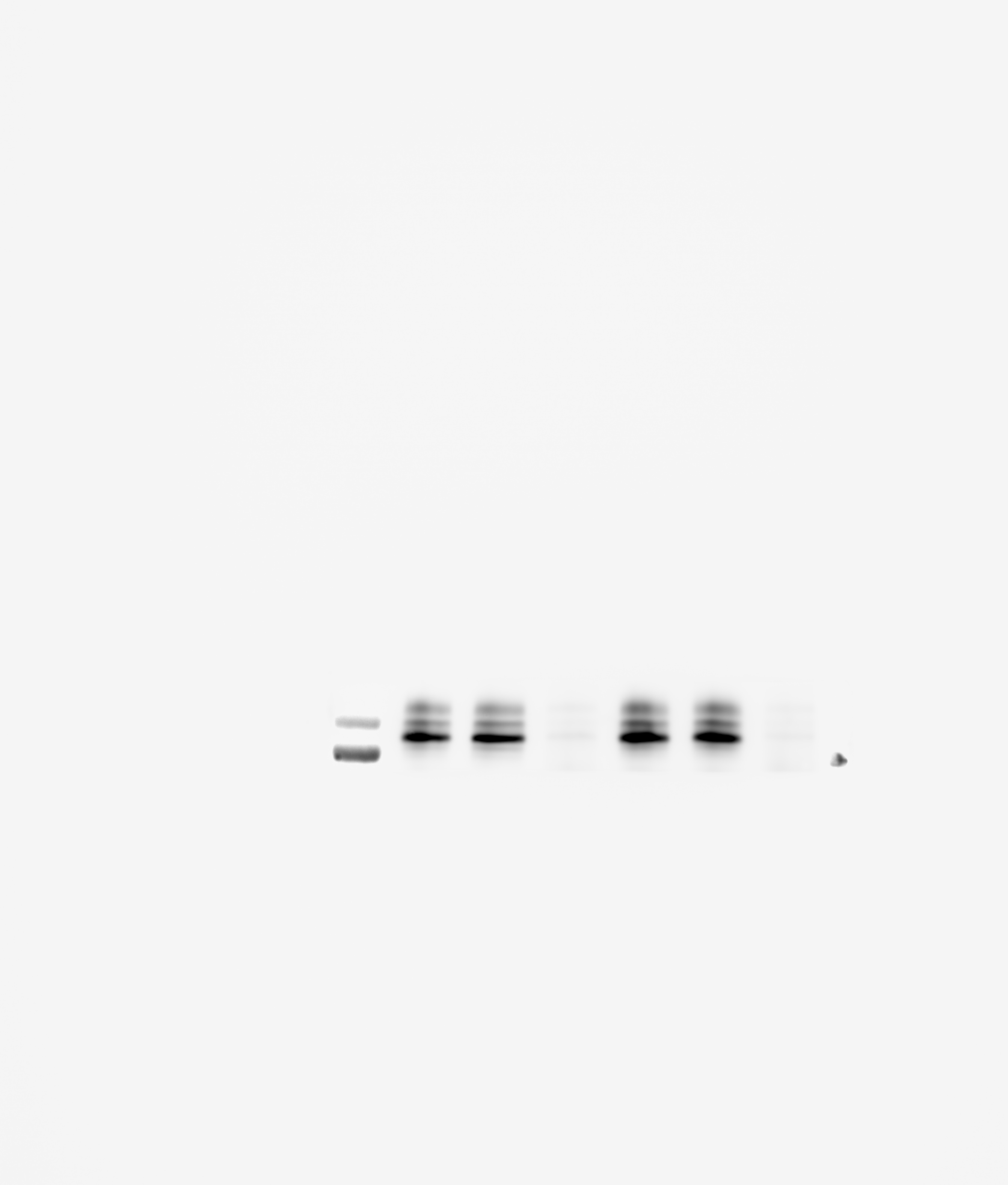

Supplement: Figure 4—figure supplement 1—source data 1. [file elife-86972-fig4-figsupp1-data1.zip › Figure 4-S1D/PSD95.tif]

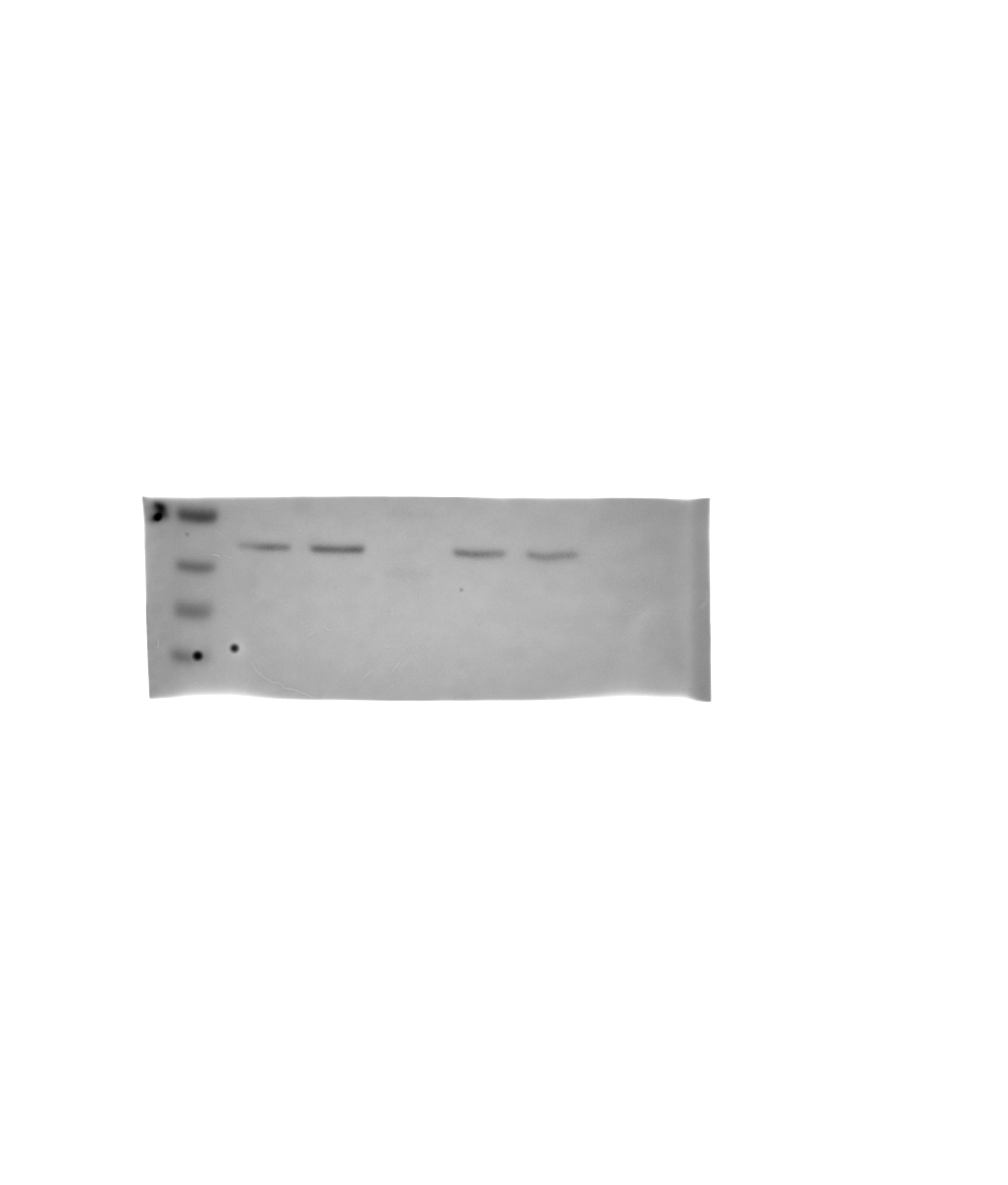

Supplement: Figure 4—figure supplement 1—source data 1. [file elife-86972-fig4-figsupp1-data1.zip › Figure 4-S1D/PSMC6.tif]

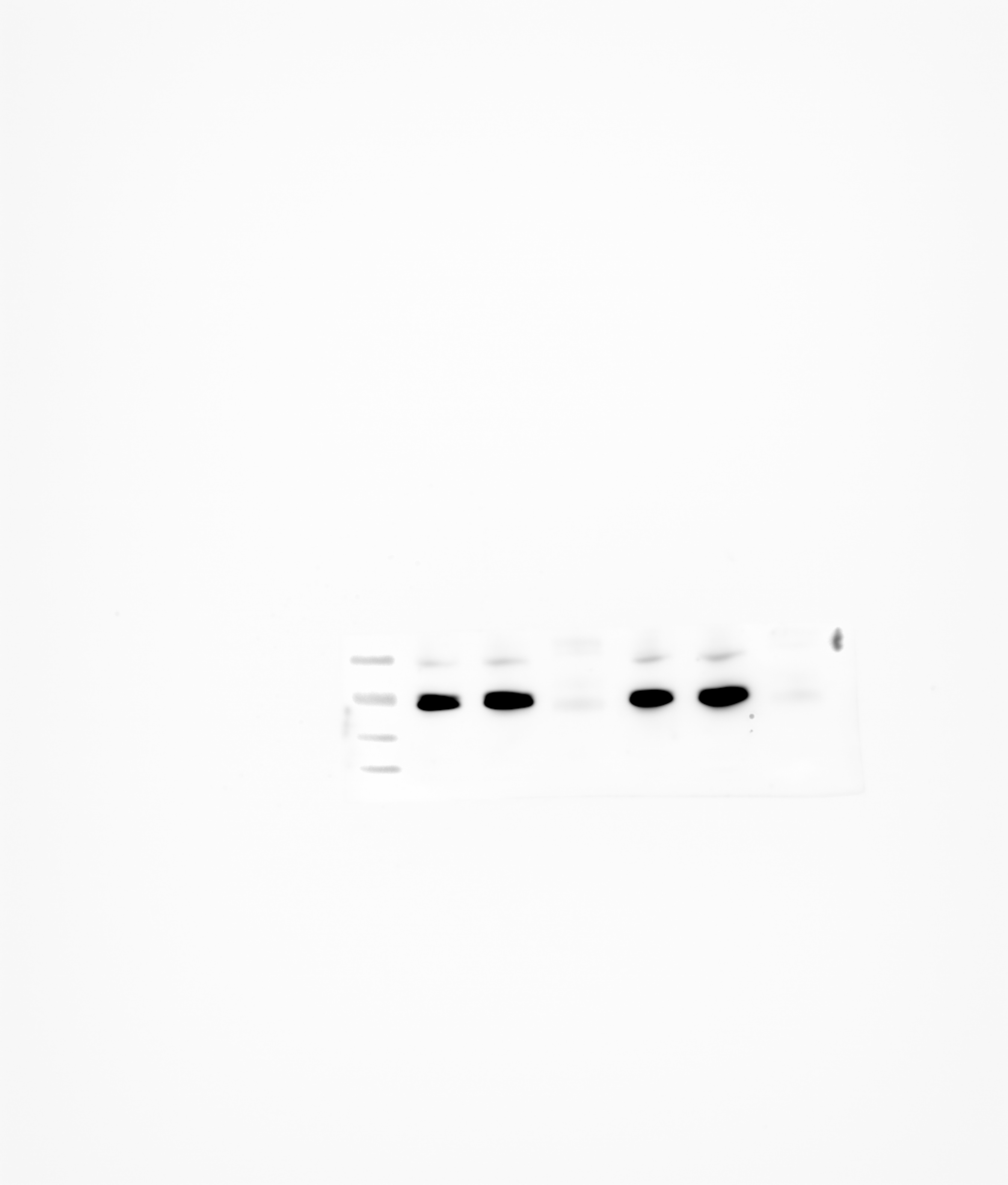

Supplement: Figure 4—figure supplement 1—source data 1. [file elife-86972-fig4-figsupp1-data1.zip › Figure 4-S1D/SNAP23.tif]

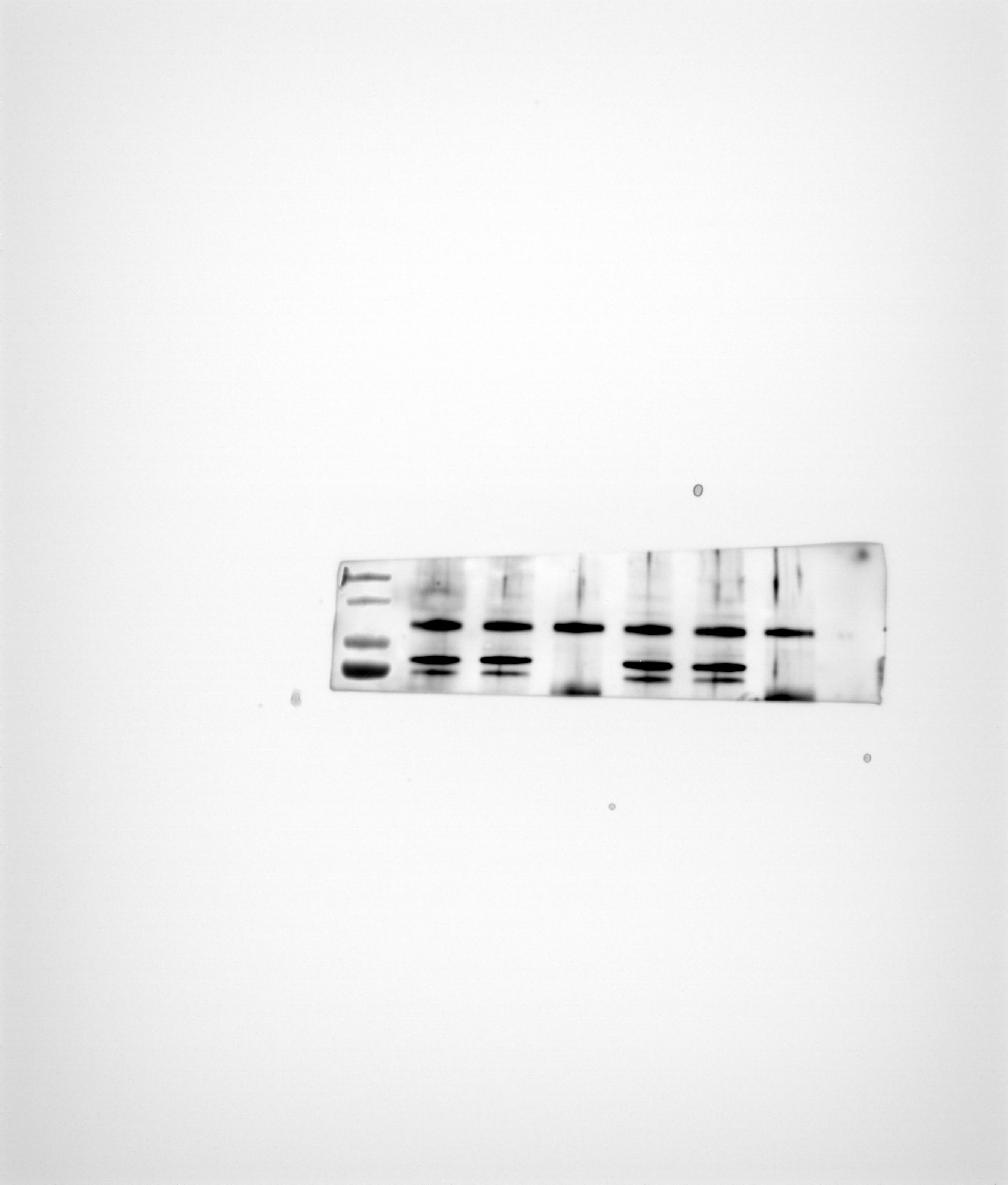

Supplement: Figure 4—figure supplement 1—source data 1. [file elife-86972-fig4-figsupp1-data1.zip › Figure 4-S1D/TR.tif]

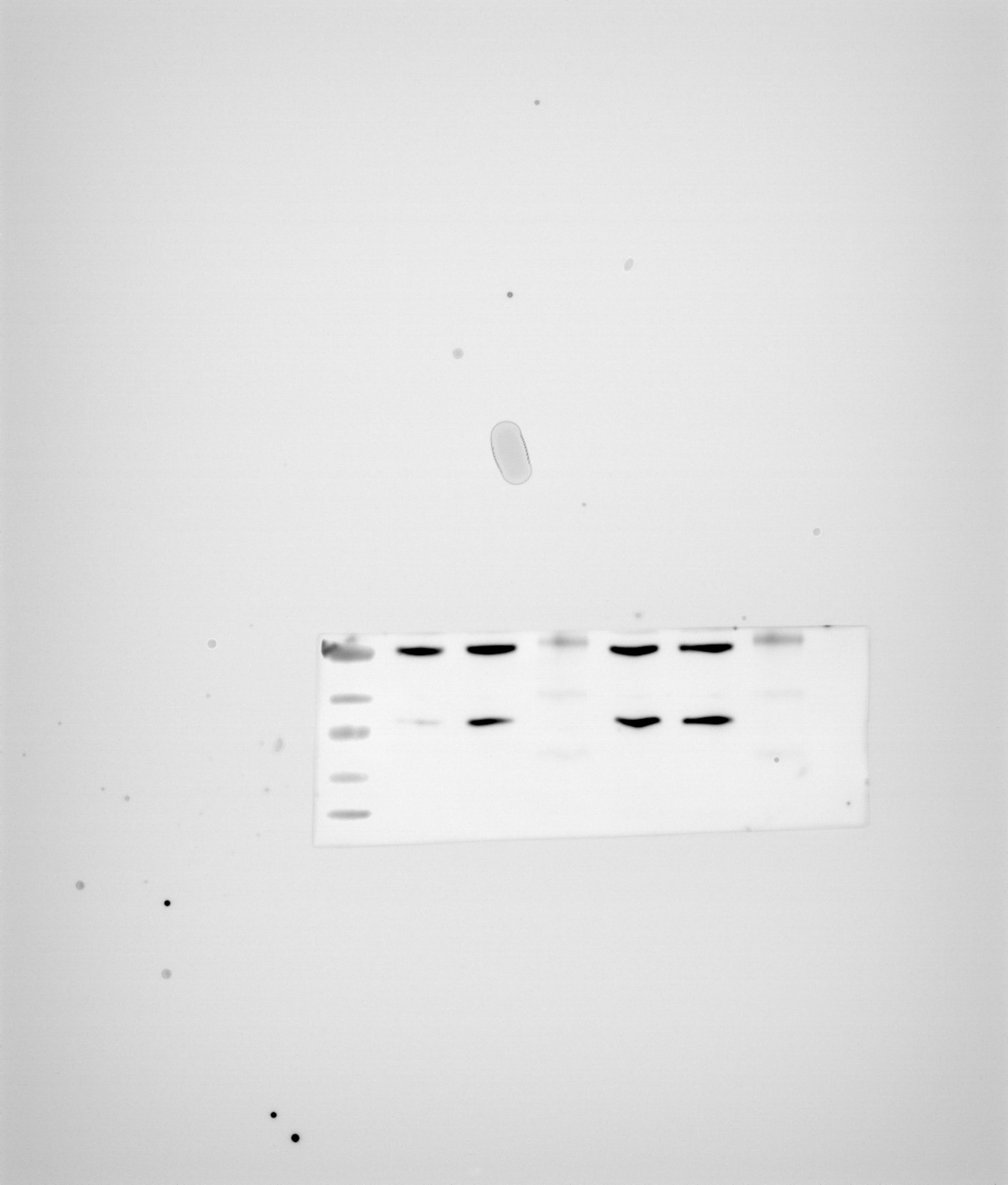

Supplement: Figure 4—figure supplement 1—source data 1. [file elife-86972-fig4-figsupp1-data1.zip › Figure 4-S1D/VDAC.tif]

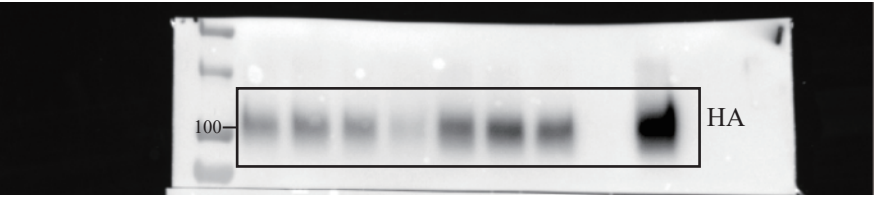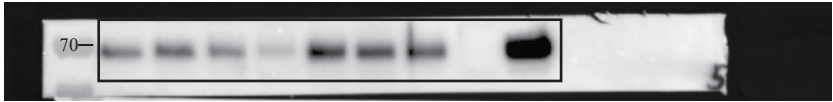

Synaptotagmin1

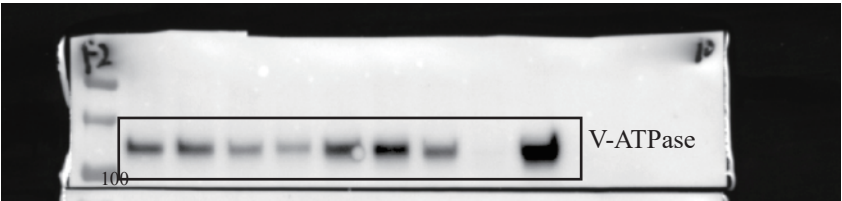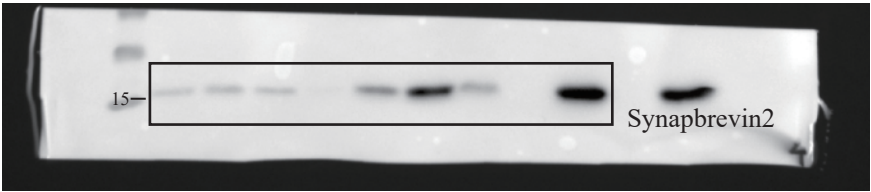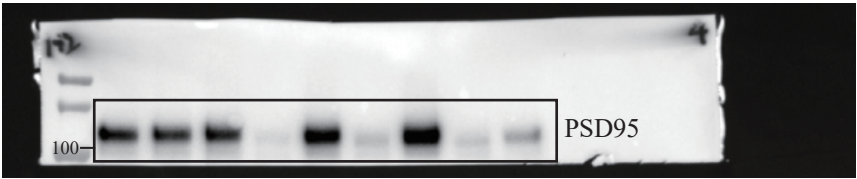

Supplement: Figure 4—figure supplement 1—source data 2. [file elife-86972-fig4-figsupp1-data2.zip › FigureS5C-Source Data-WB.pdf]

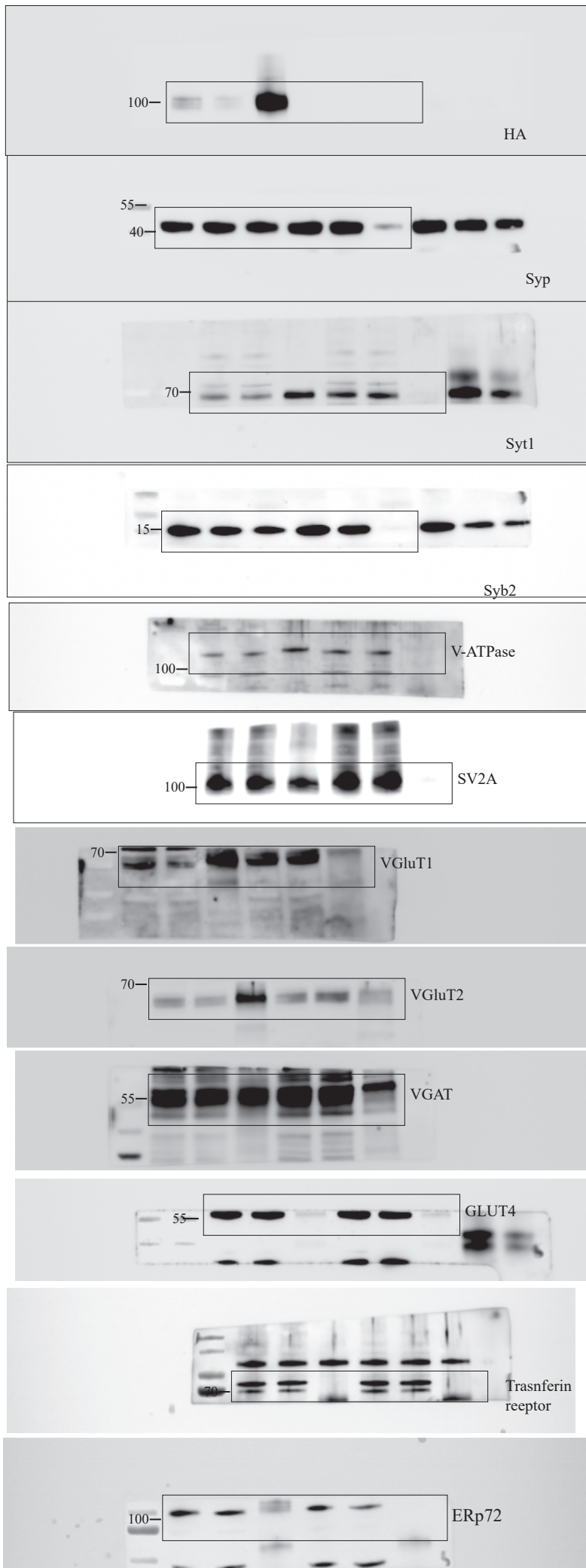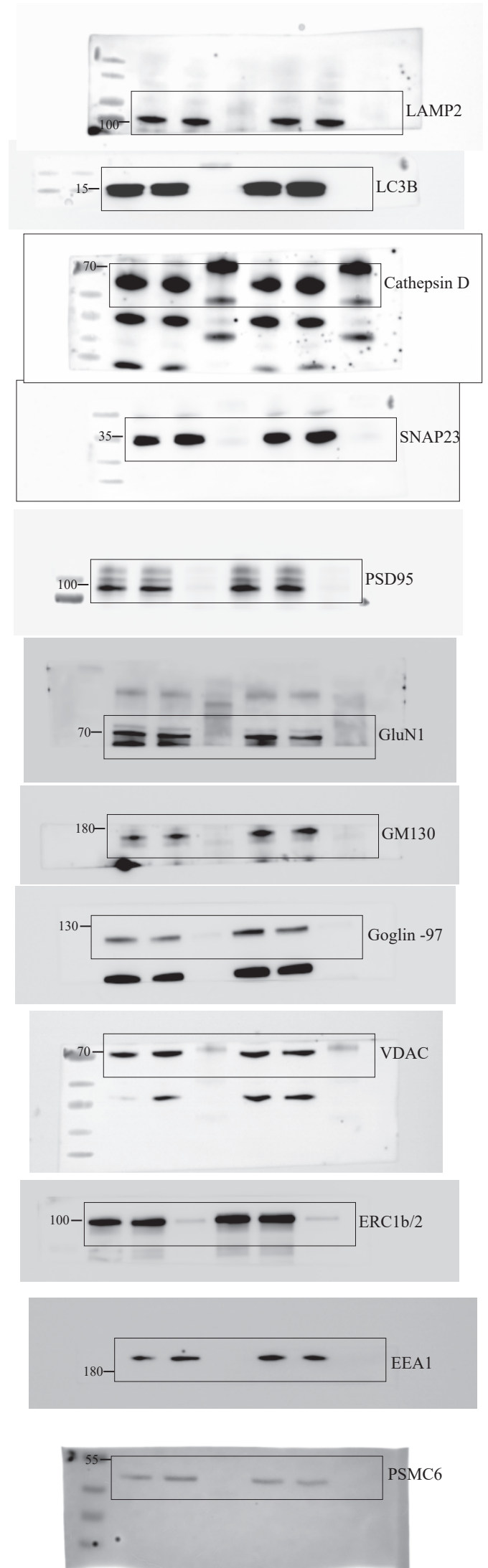

Supplement: Figure 4—figure supplement 1—source data 2. [file elife-86972-fig4-figsupp1-data2.zip › FigureS5D-Source Data-WB.pdf]

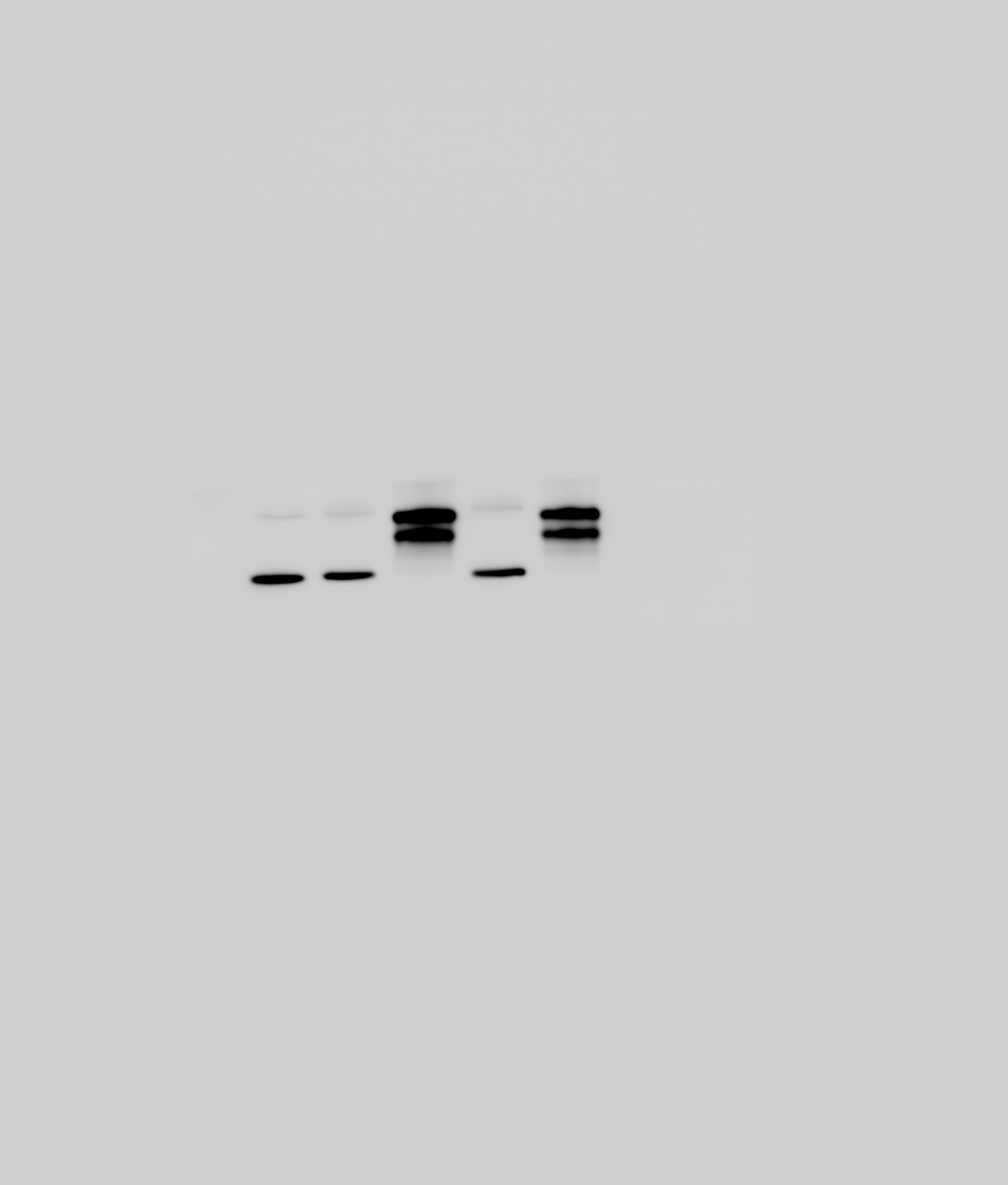

Supplement: Figure 5—figure supplement 1—source data 1. [file elife-86972-fig5-figsupp1-data1.zip › Figure 5-S1A/Cathepsin D.tif]

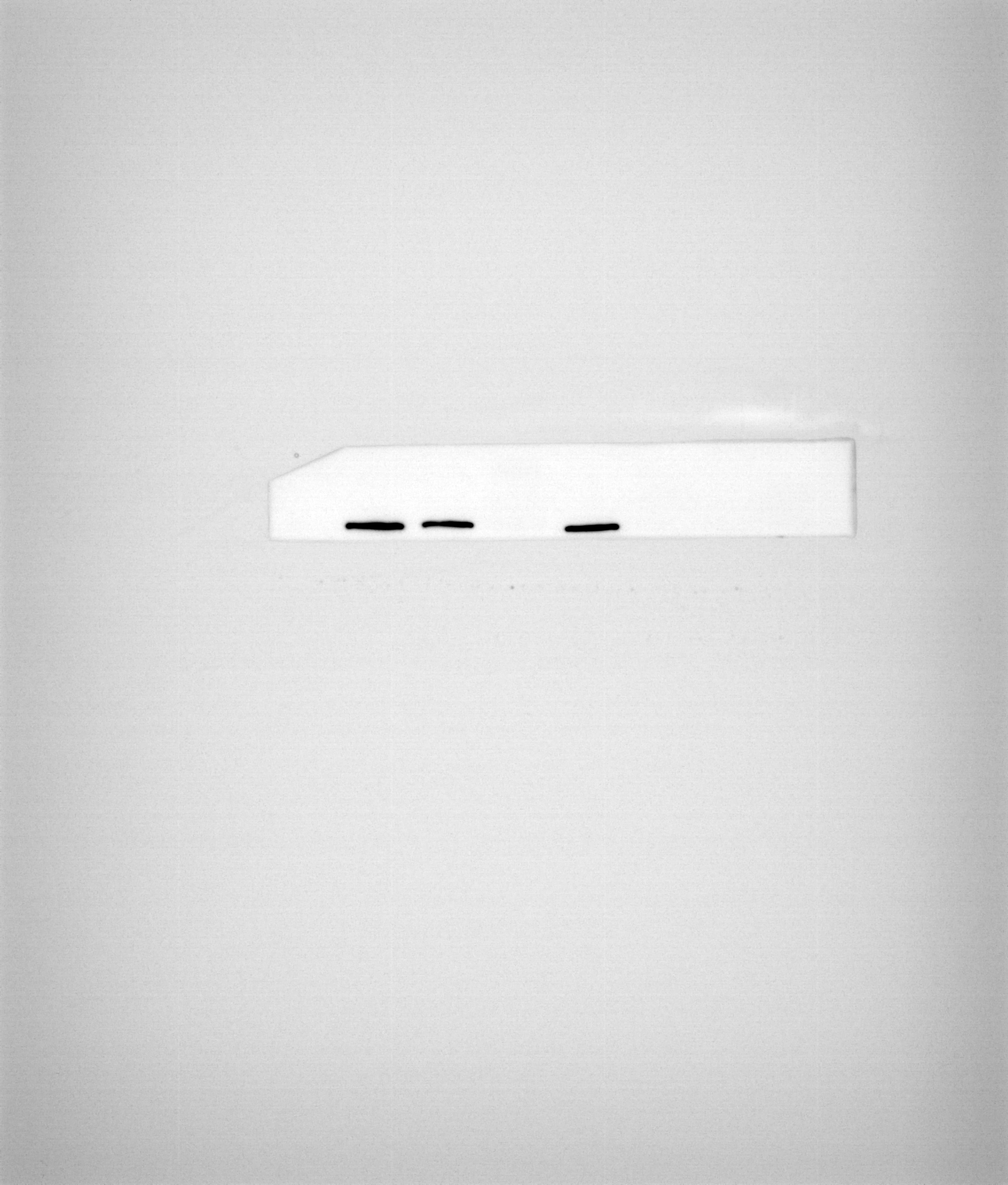

Supplement: Figure 5—figure supplement 1—source data 1. [file elife-86972-fig5-figsupp1-data1.zip › Figure 5-S1A/EEA1.tif]

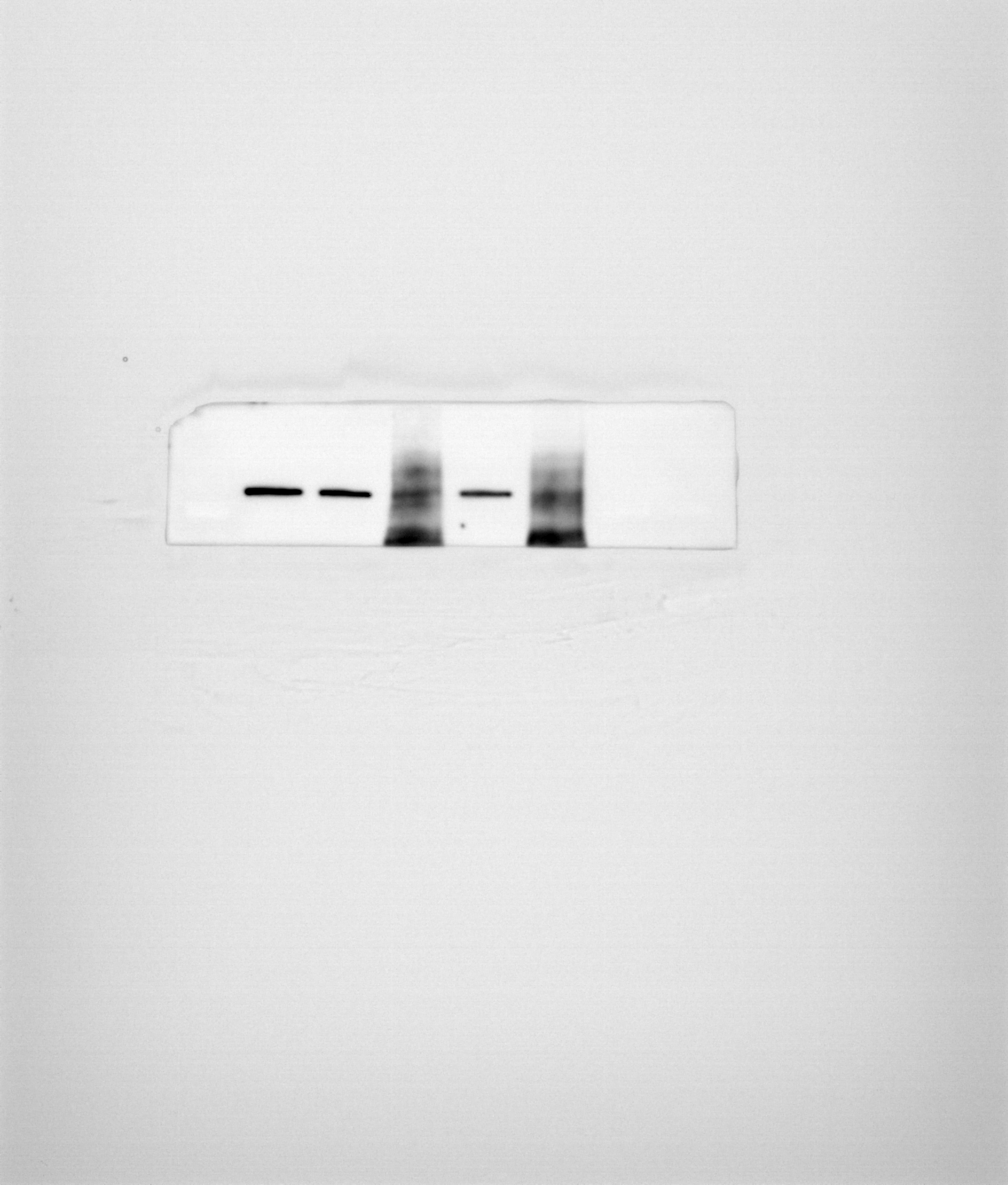

Supplement: Figure 5—figure supplement 1—source data 1. [file elife-86972-fig5-figsupp1-data1.zip › Figure 5-S1A/ERp72.tif]

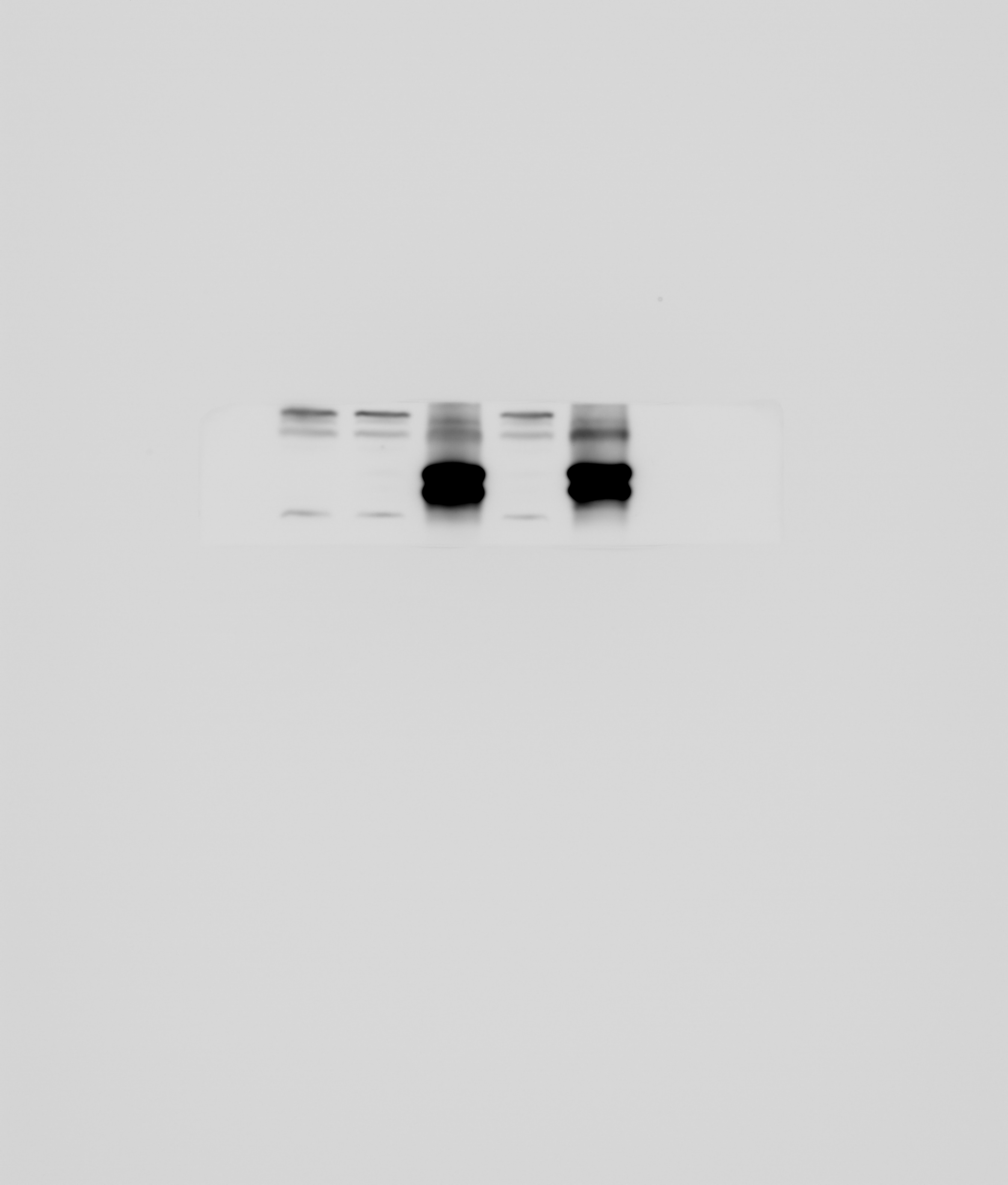

Supplement: Figure 5—figure supplement 1—source data 1. [file elife-86972-fig5-figsupp1-data1.zip › Figure 5-S1A/GluT4.tif]

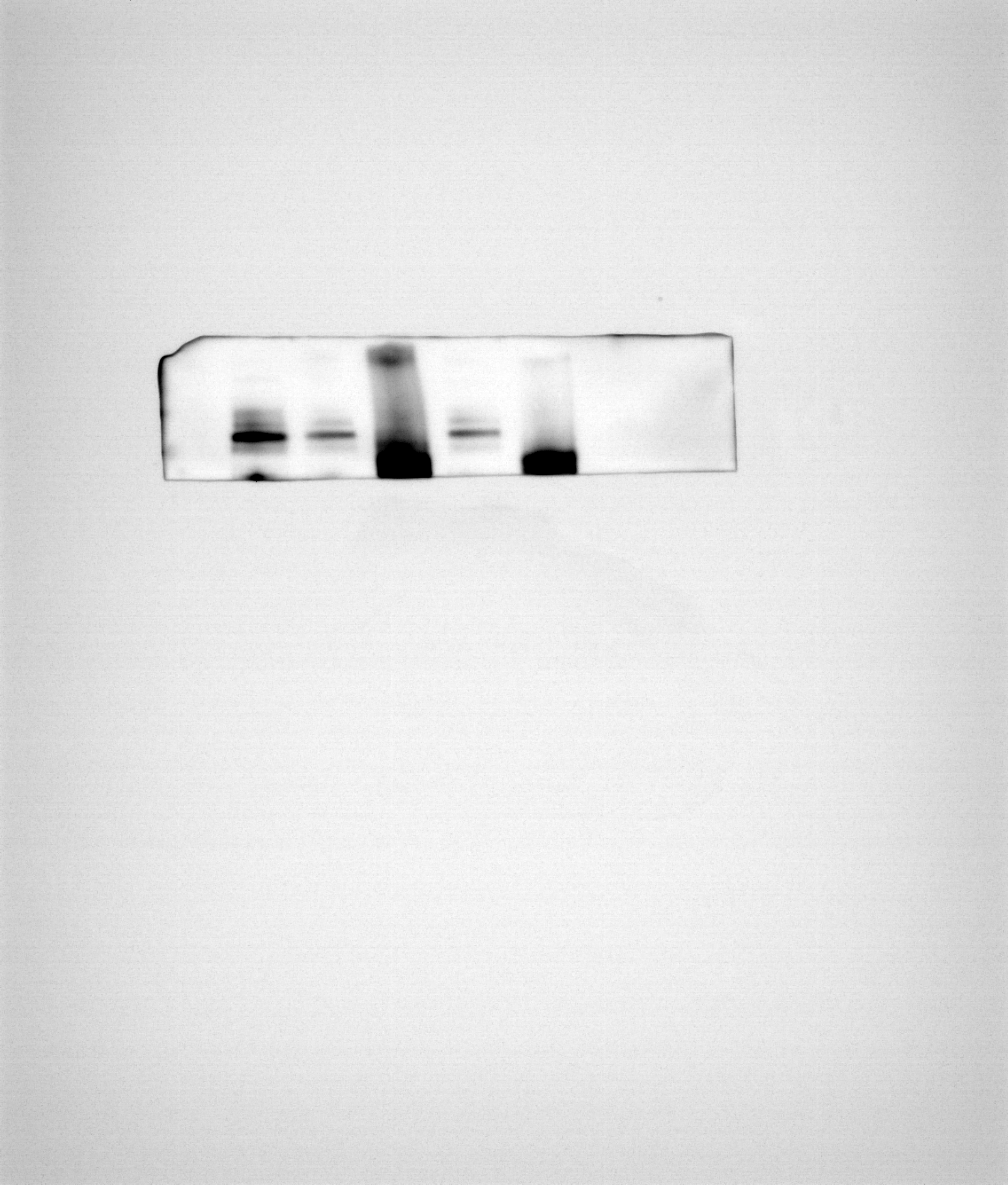

Supplement: Figure 5—figure supplement 1—source data 1. [file elife-86972-fig5-figsupp1-data1.zip › Figure 5-S1A/GM130.tif]

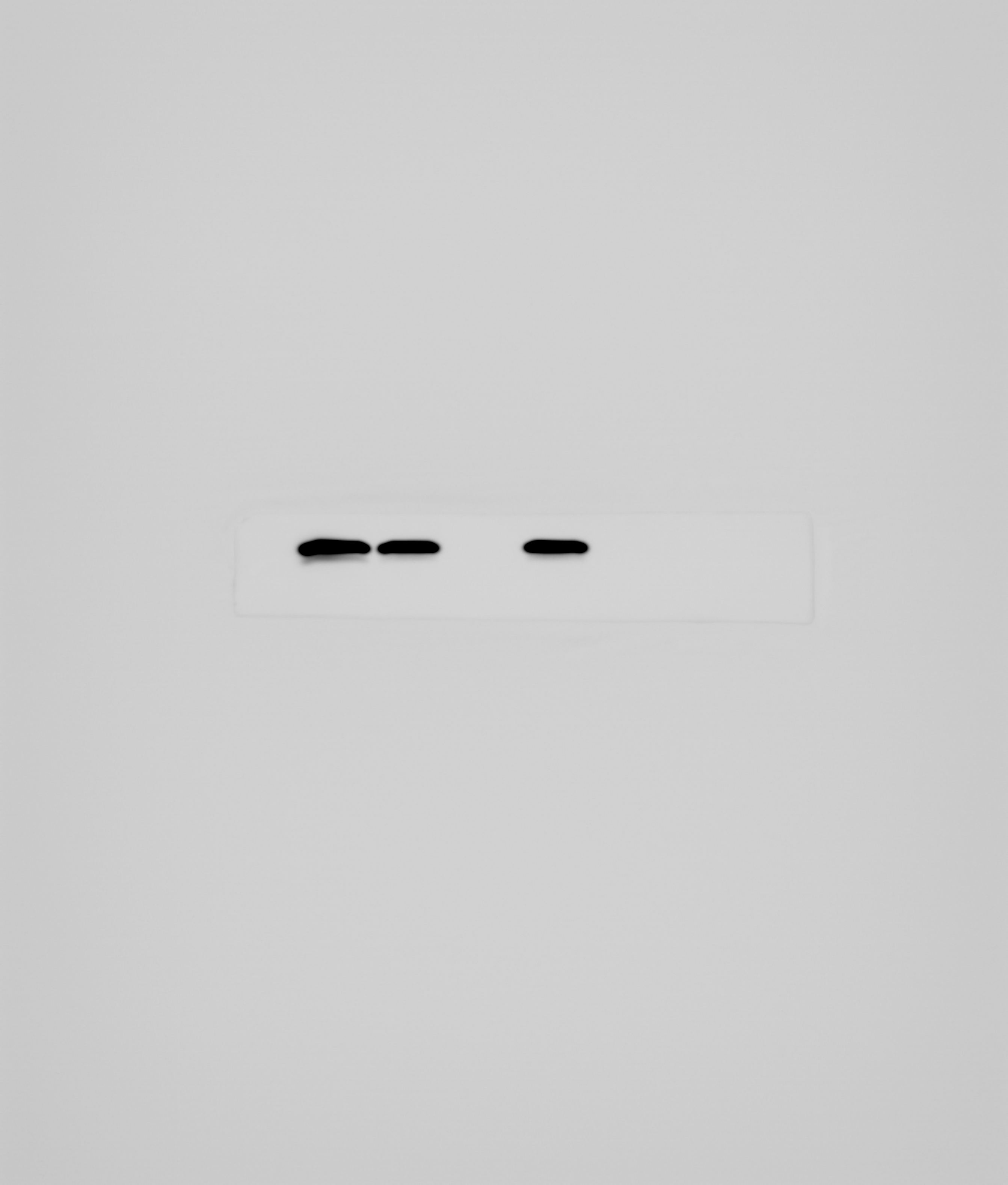

Supplement: Figure 5—figure supplement 1—source data 1. [file elife-86972-fig5-figsupp1-data1.zip › Figure 5-S1A/LC3B.tif]

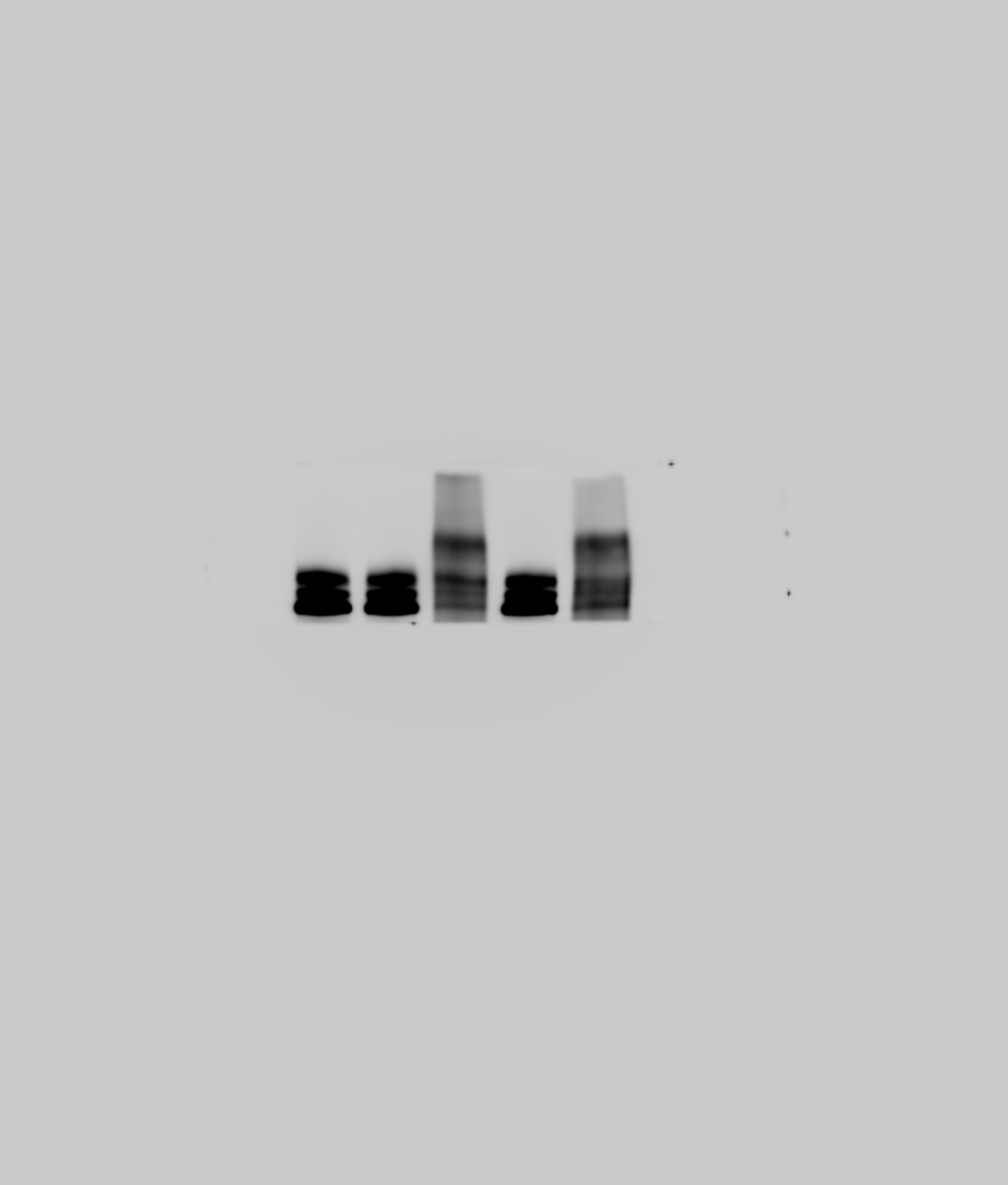

Supplement: Figure 5—figure supplement 1—source data 1. [file elife-86972-fig5-figsupp1-data1.zip › Figure 5-S1A/PSD95.tif]

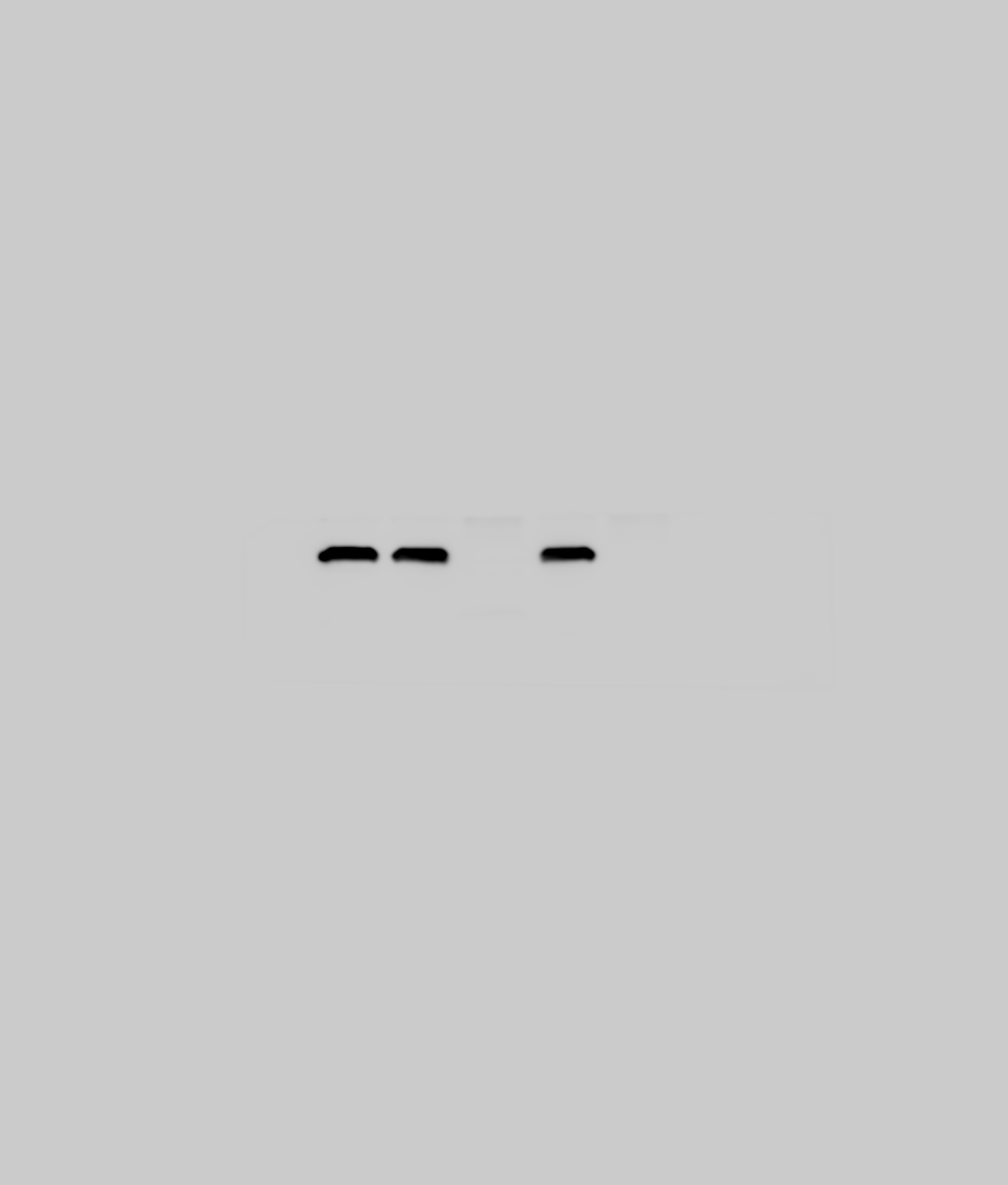

Supplement: Figure 5—figure supplement 1—source data 1. [file elife-86972-fig5-figsupp1-data1.zip › Figure 5-S1A/SNAP23.tif]

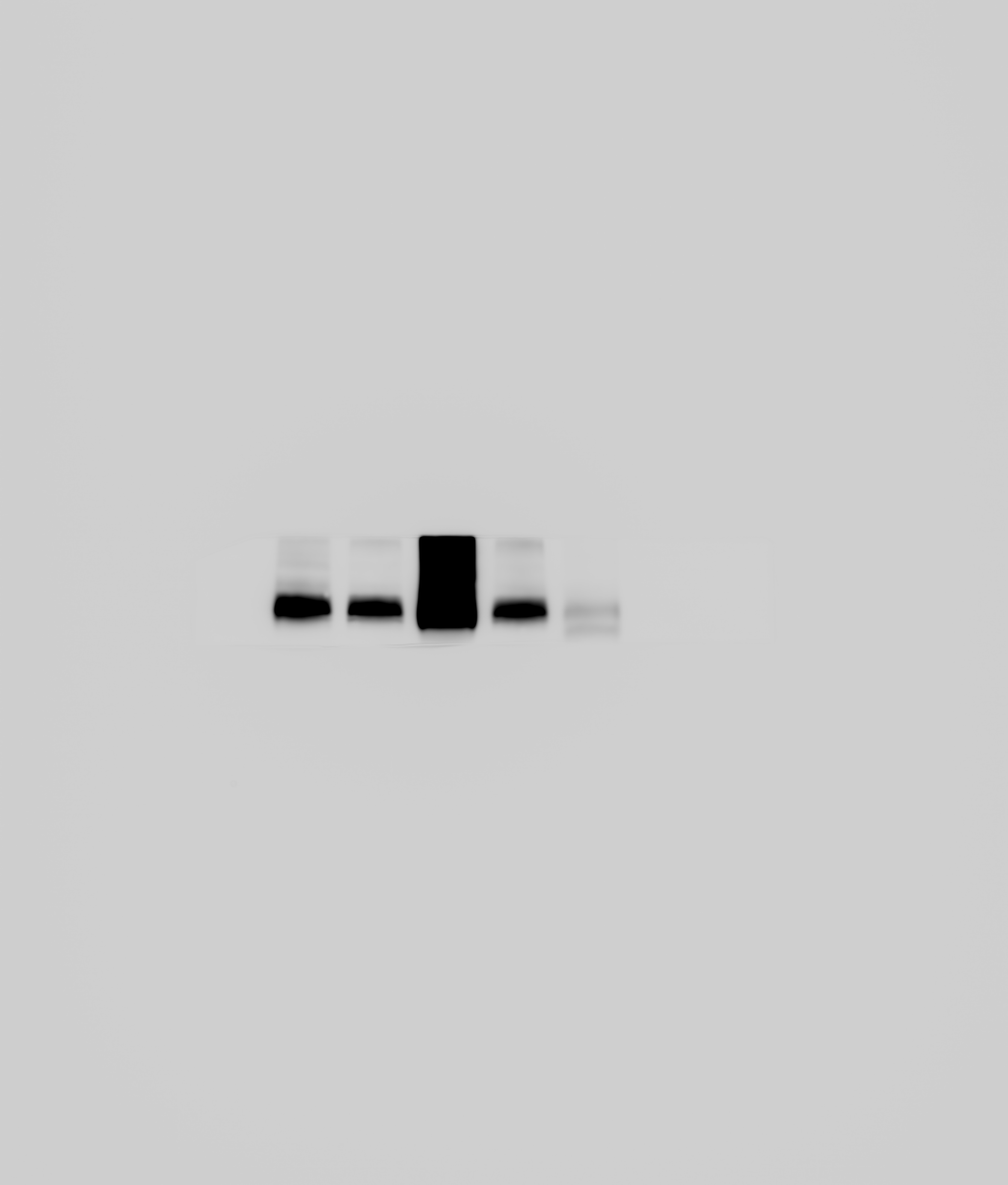

Supplement: Figure 5—figure supplement 1—source data 1. [file elife-86972-fig5-figsupp1-data1.zip › Figure 5-S1A/SV2A.tif]

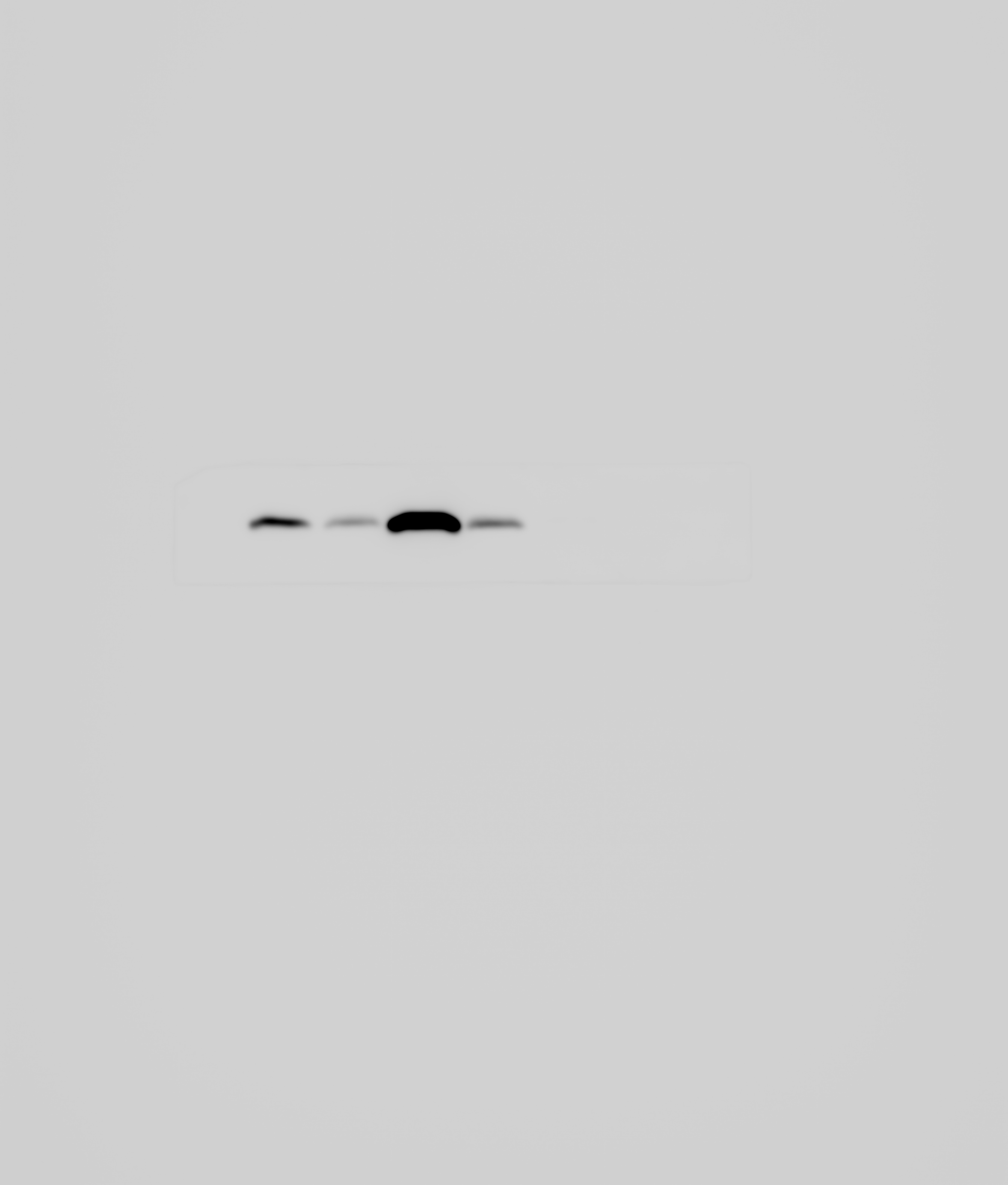

Supplement: Figure 5—figure supplement 1—source data 1. [file elife-86972-fig5-figsupp1-data1.zip › Figure 5-S1A/Syb2.tif]

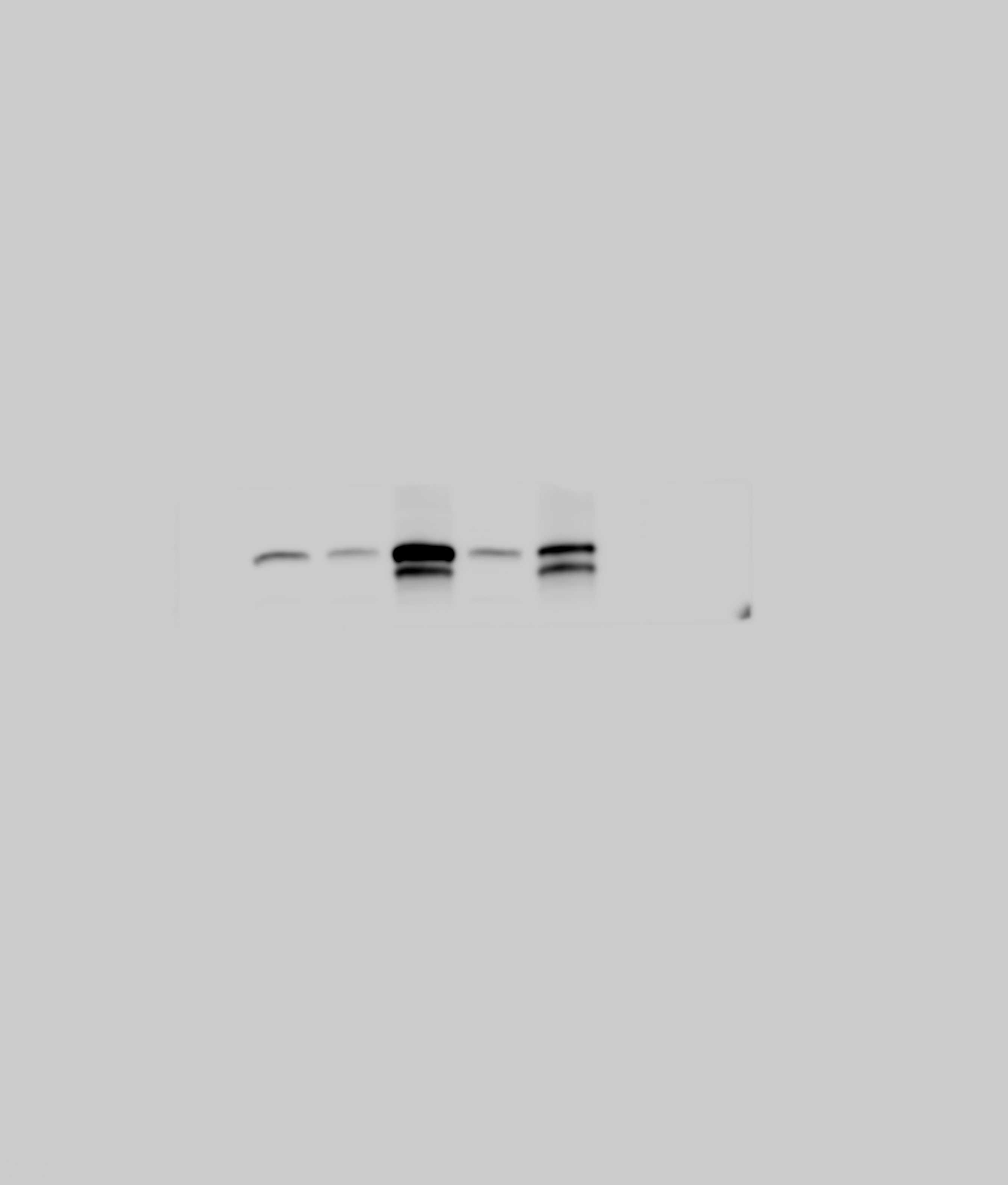

Supplement: Figure 5—figure supplement 1—source data 1. [file elife-86972-fig5-figsupp1-data1.zip › Figure 5-S1A/Syp.tif]

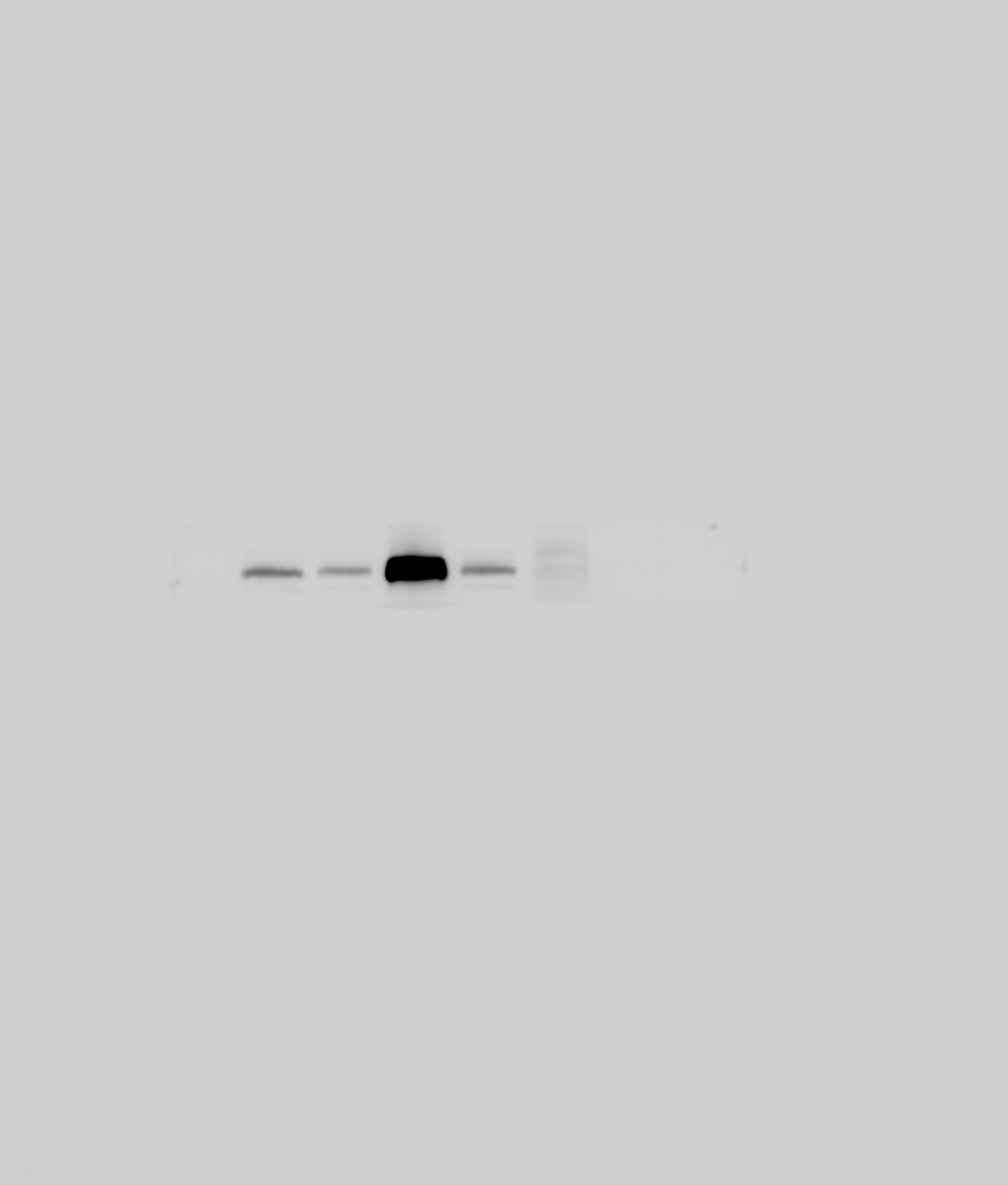

Supplement: Figure 5—figure supplement 1—source data 1. [file elife-86972-fig5-figsupp1-data1.zip › Figure 5-S1A/Syt1.tif]

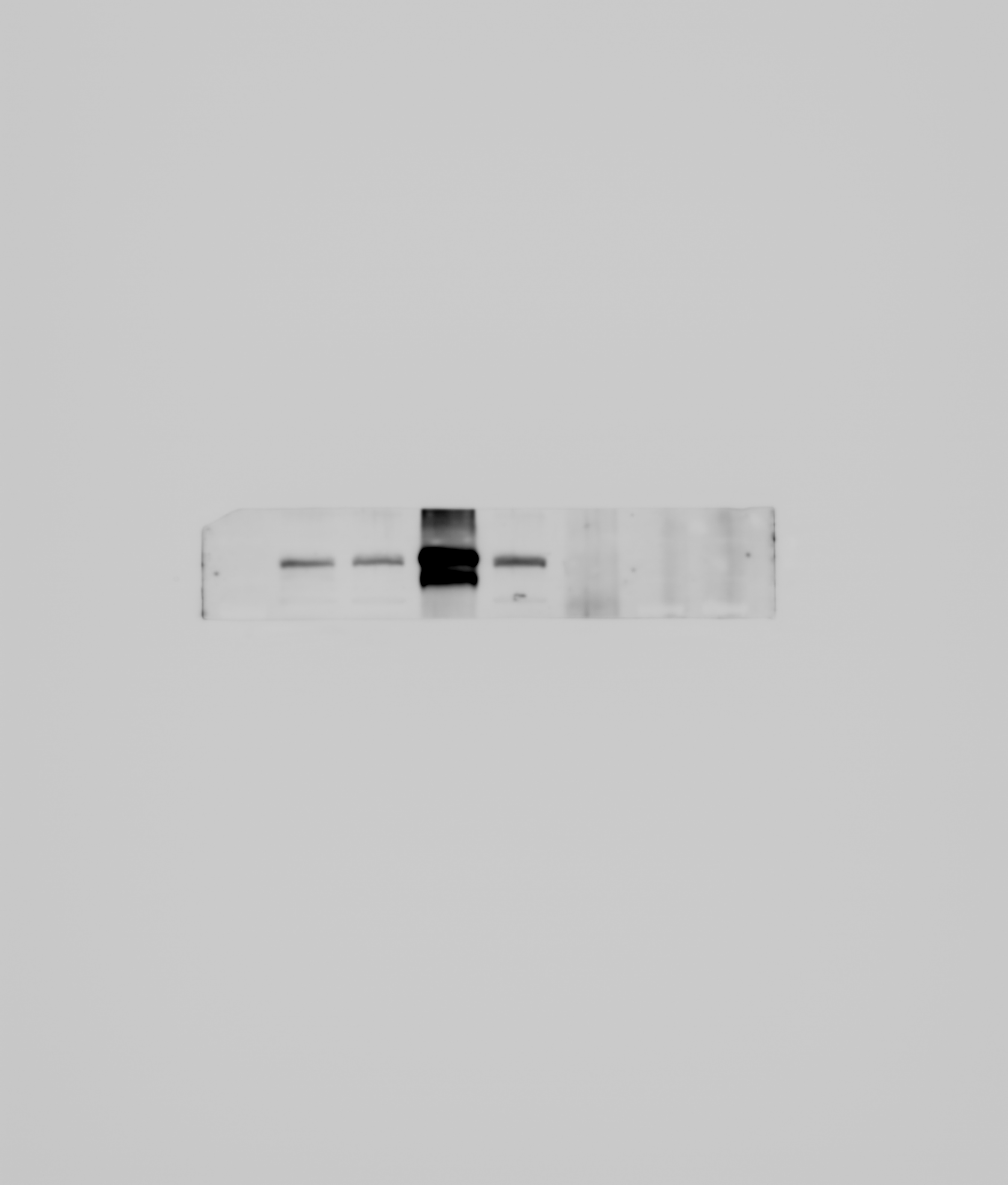

Supplement: Figure 5—figure supplement 1—source data 1. [file elife-86972-fig5-figsupp1-data1.zip › Figure 5-S1A/V-ATPase.tif]

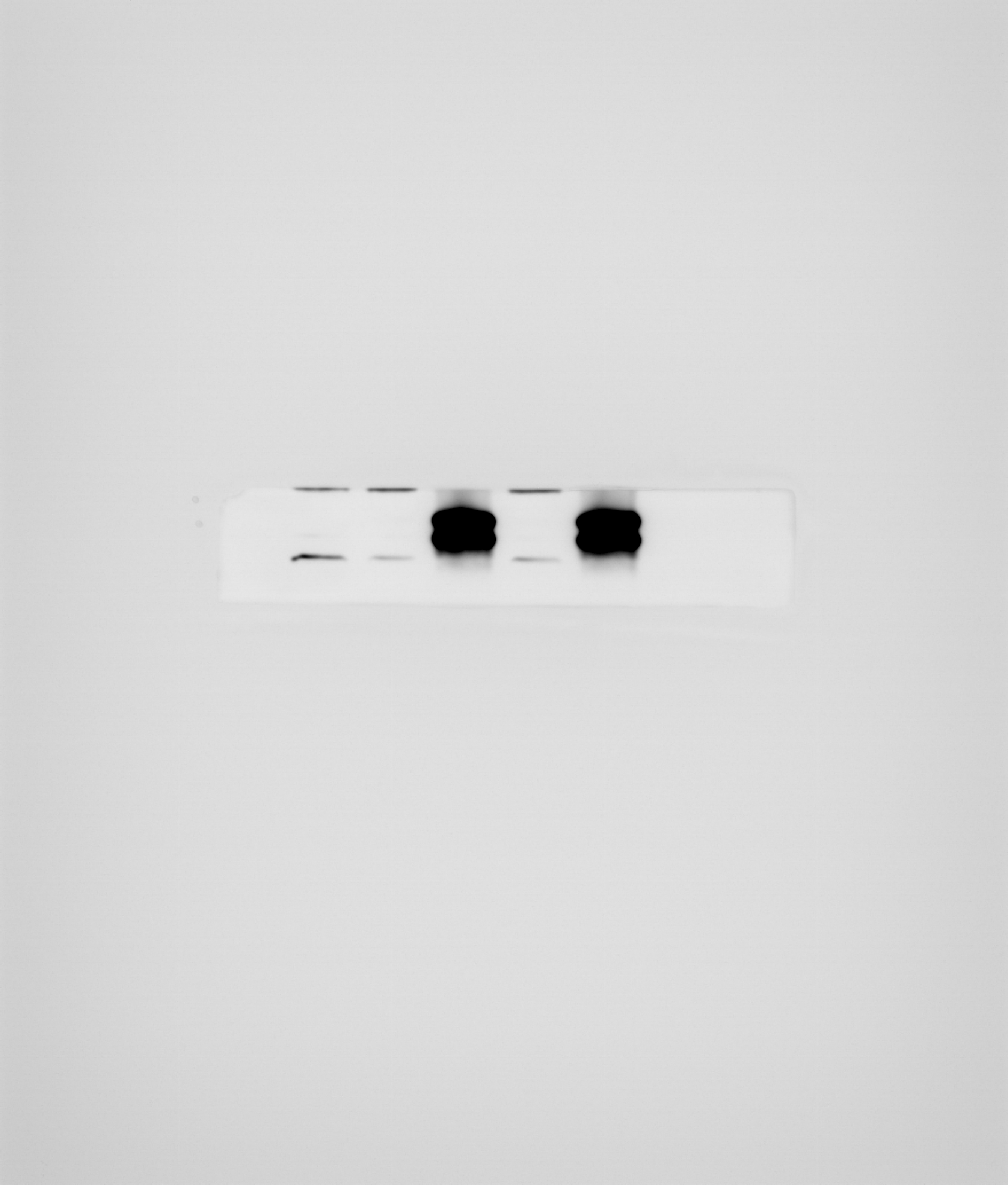

Supplement: Figure 5—figure supplement 1—source data 1. [file elife-86972-fig5-figsupp1-data1.zip › Figure 5-S1A/VDAC.tif]

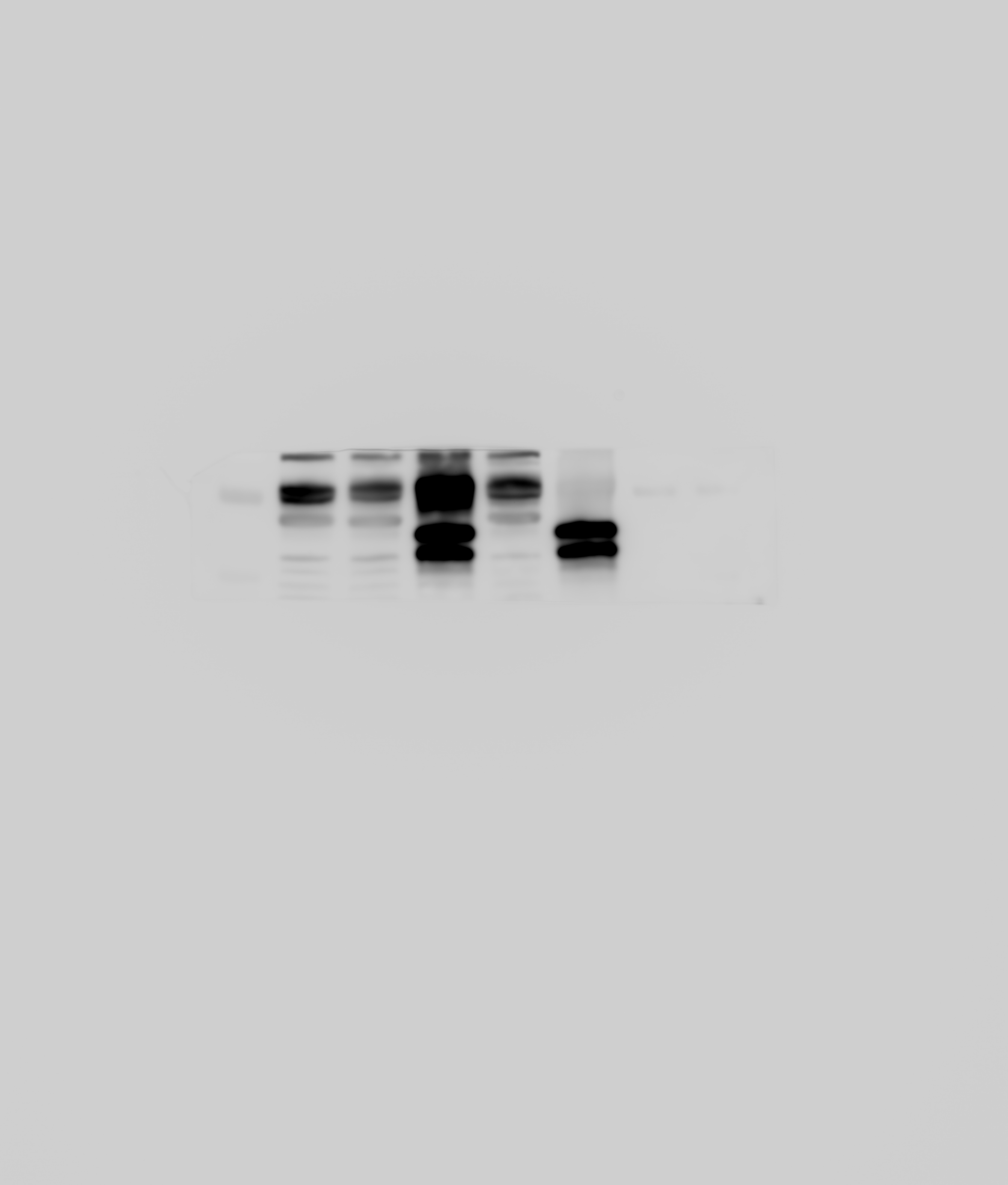

Supplement: Figure 5—figure supplement 1—source data 1. [file elife-86972-fig5-figsupp1-data1.zip › Figure 5-S1A/VGAT.tif]

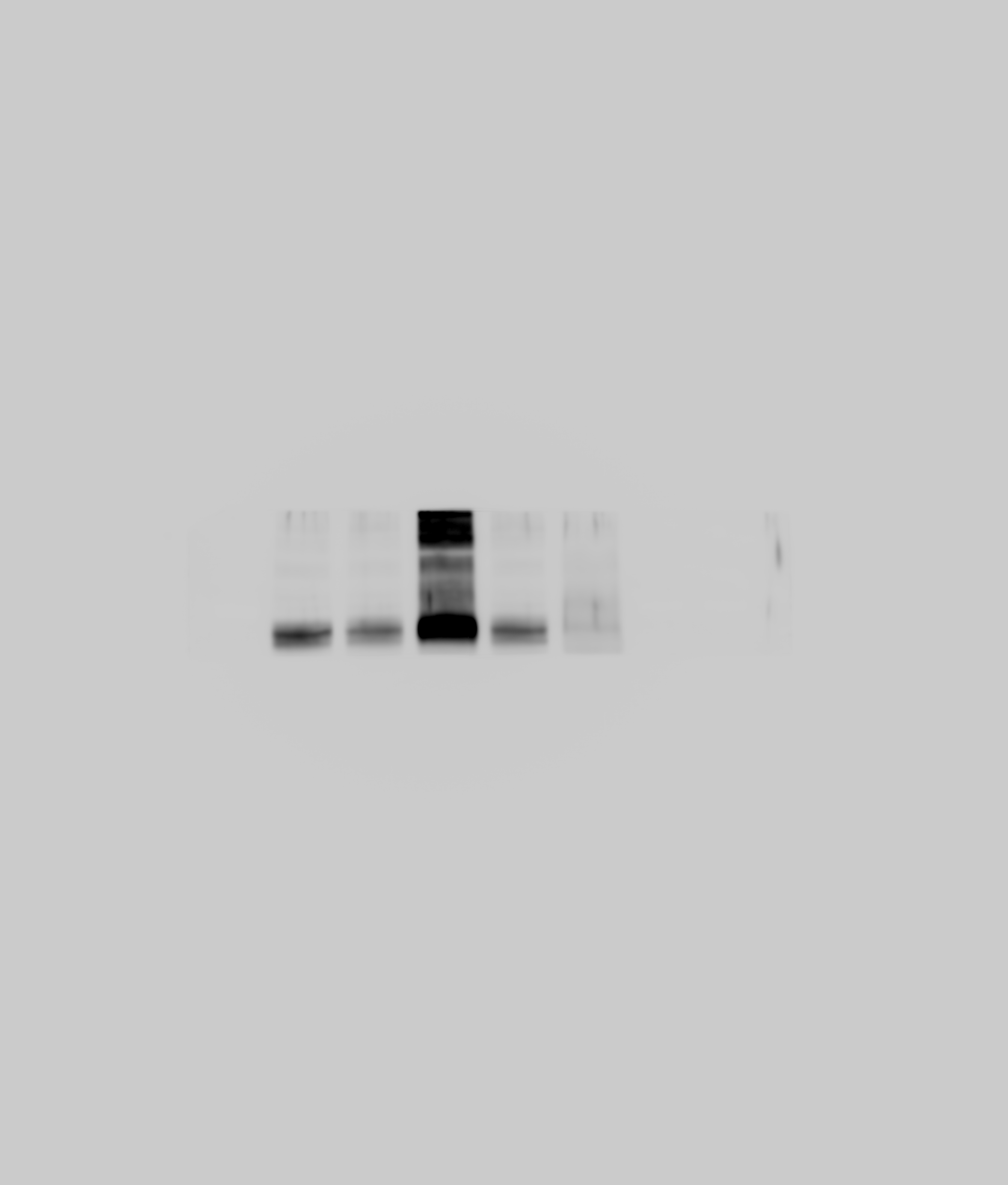

Supplement: Figure 5—figure supplement 1—source data 1. [file elife-86972-fig5-figsupp1-data1.zip › Figure 5-S1A/VGluT2.tif]

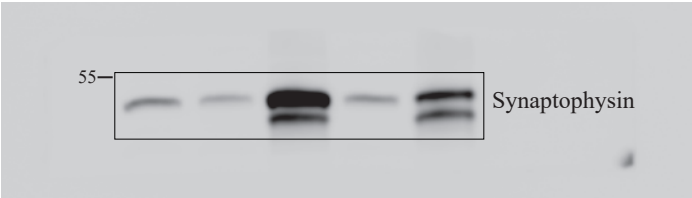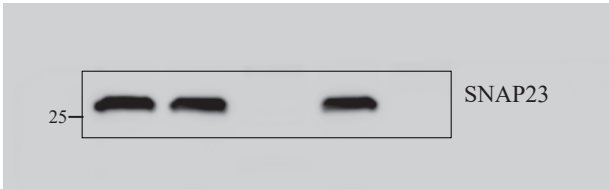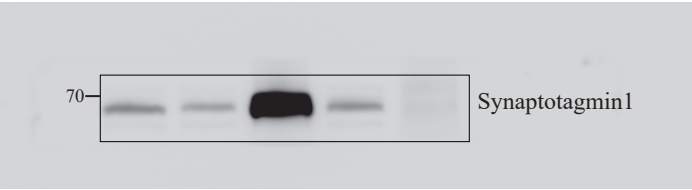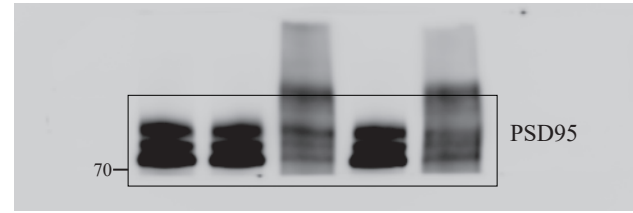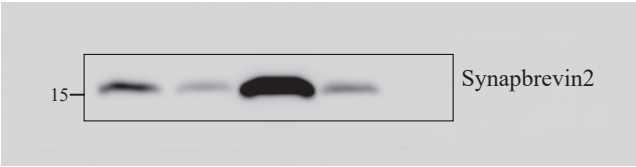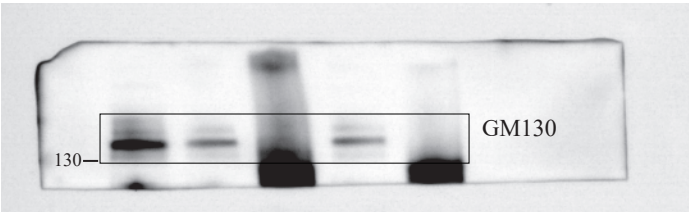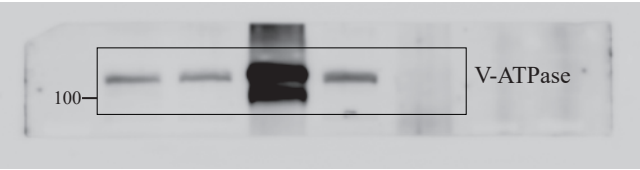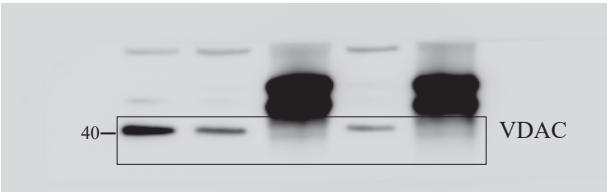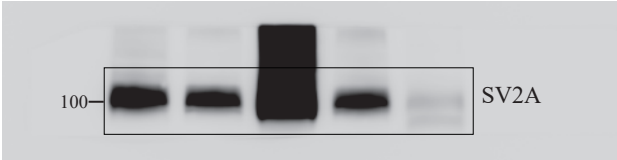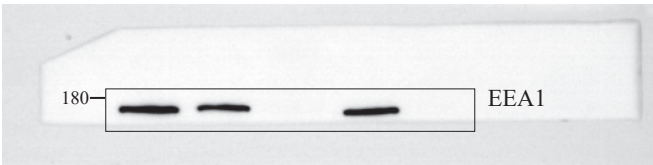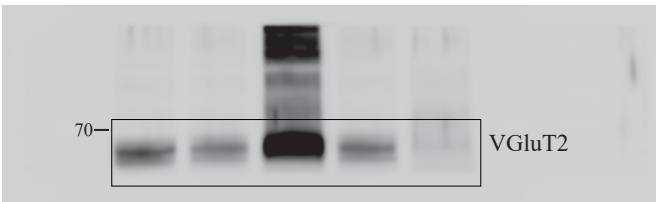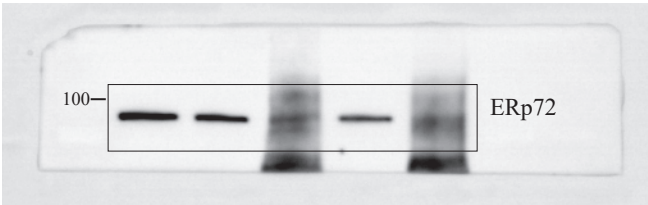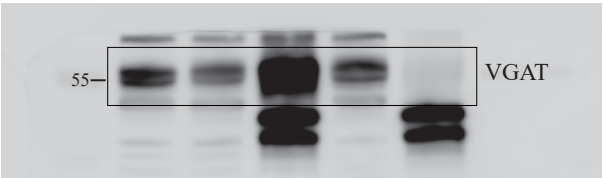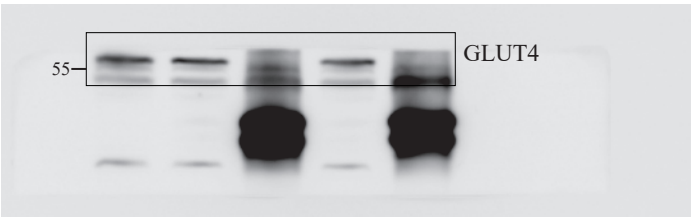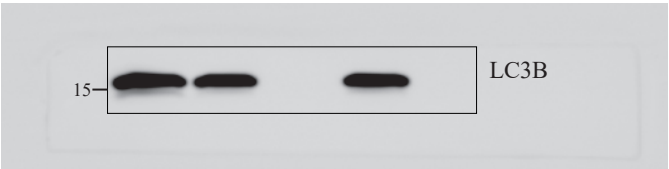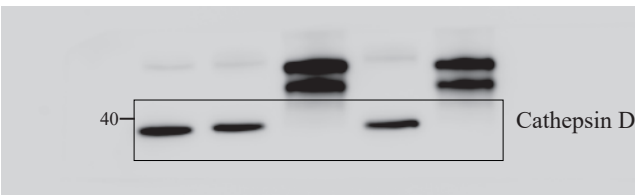

Supplement: Figure 5—figure supplement 1—source data 2. [file elife-86972-fig5-figsupp1-data2.zip › FigureS8A-Source Data-WB.pdf]

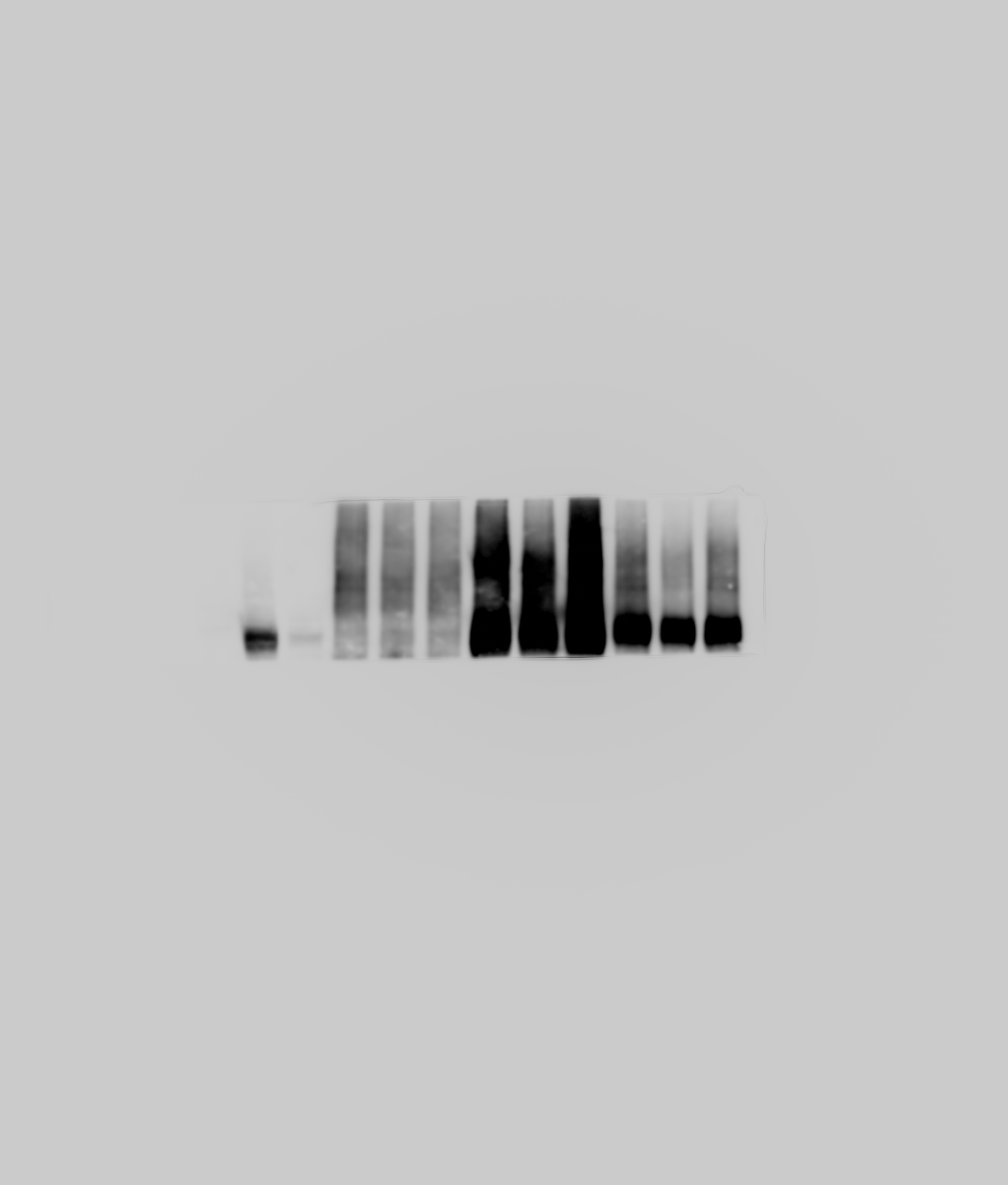

Supplement: Figure 7—figure supplement 1—source data 1. [file elife-86972-fig7-figsupp1-data1.zip › Figure 7-S1A/HA-1s-sample.tif]

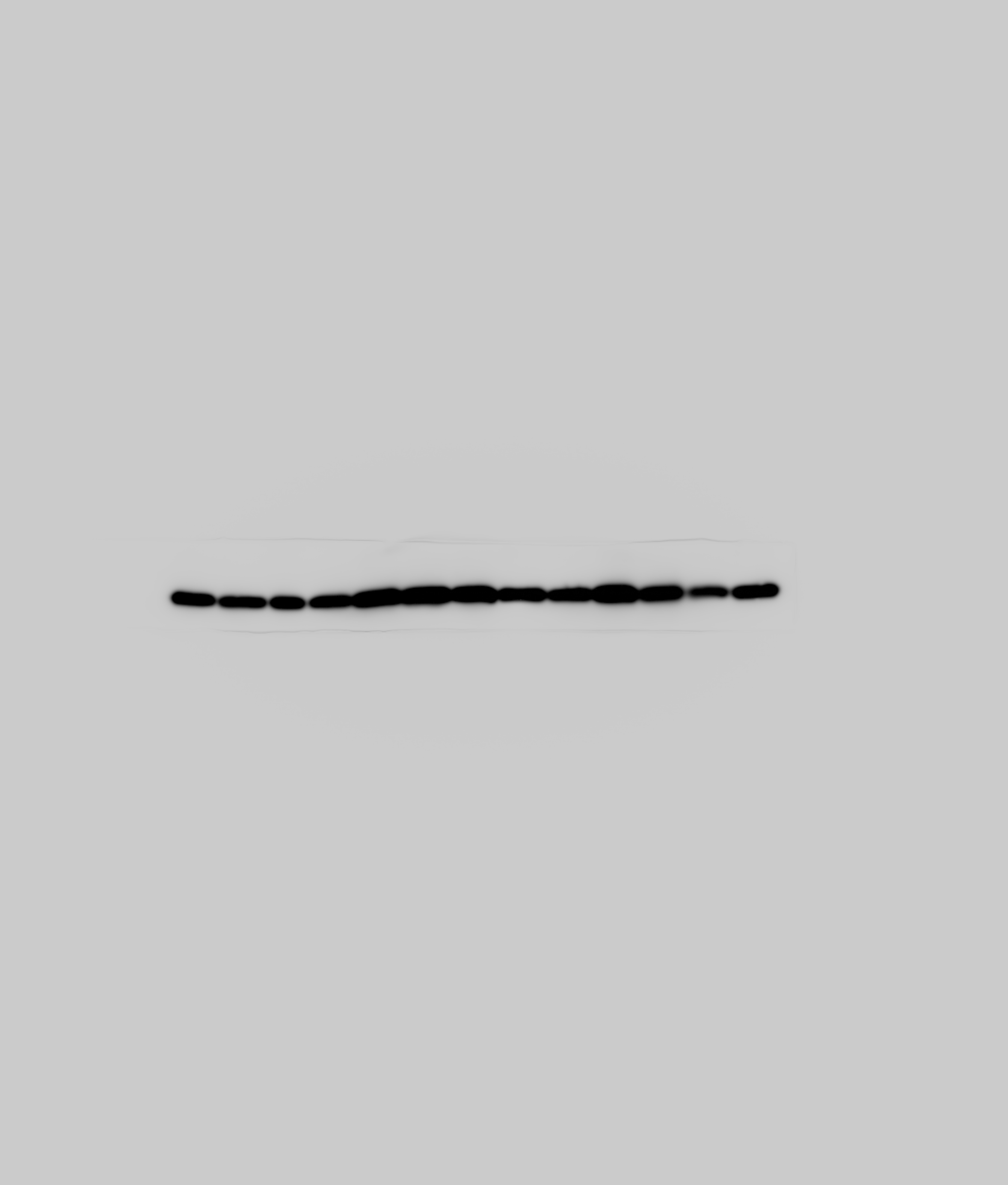

Supplement: Figure 7—figure supplement 1—source data 1. [file elife-86972-fig7-figsupp1-data1.zip › Figure 7-S1A/Syb2-1-sample.tif]

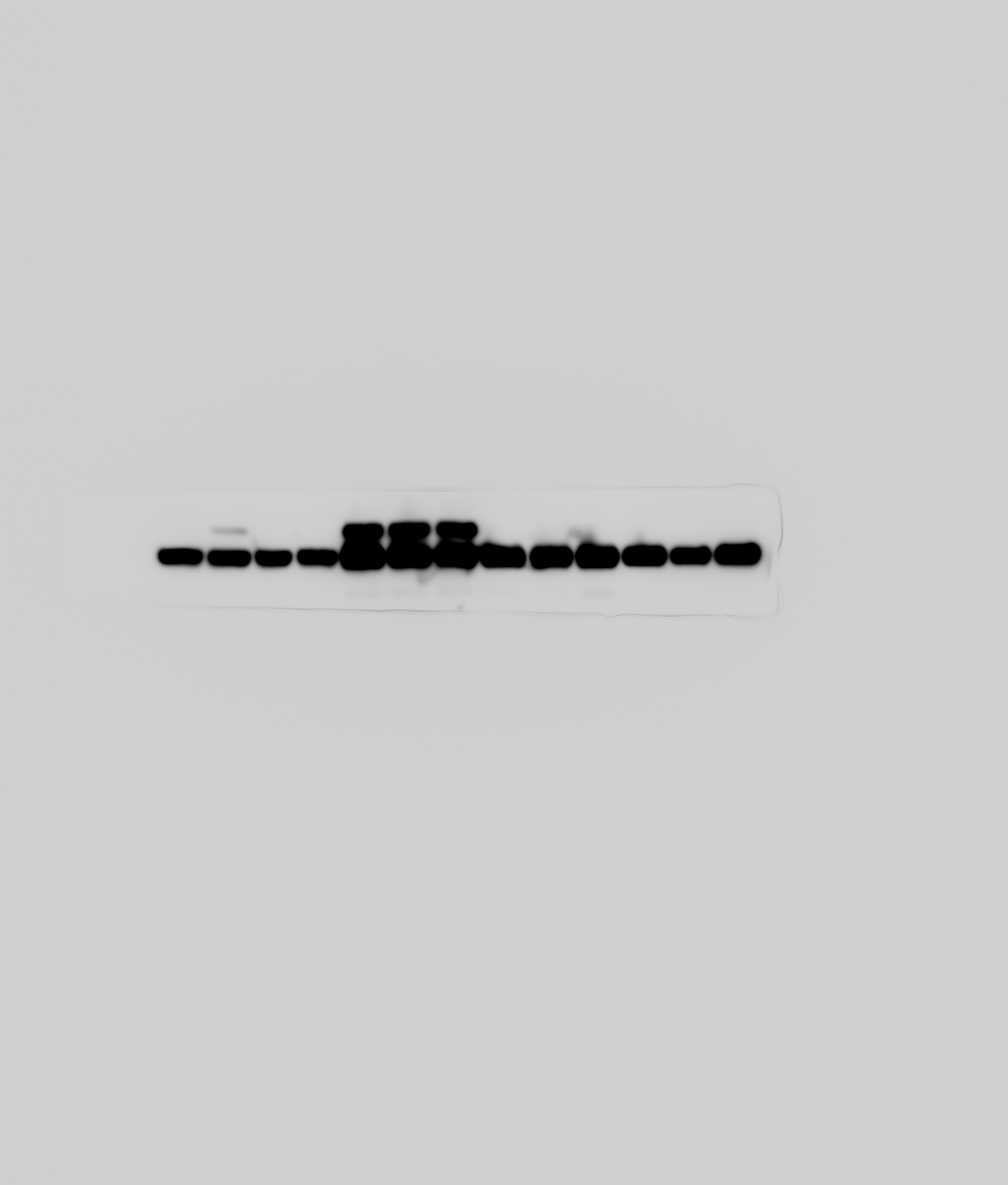

Supplement: Figure 7—figure supplement 1—source data 1. [file elife-86972-fig7-figsupp1-data1.zip › Figure 7-S1A/Syp-2s-sample.tif]

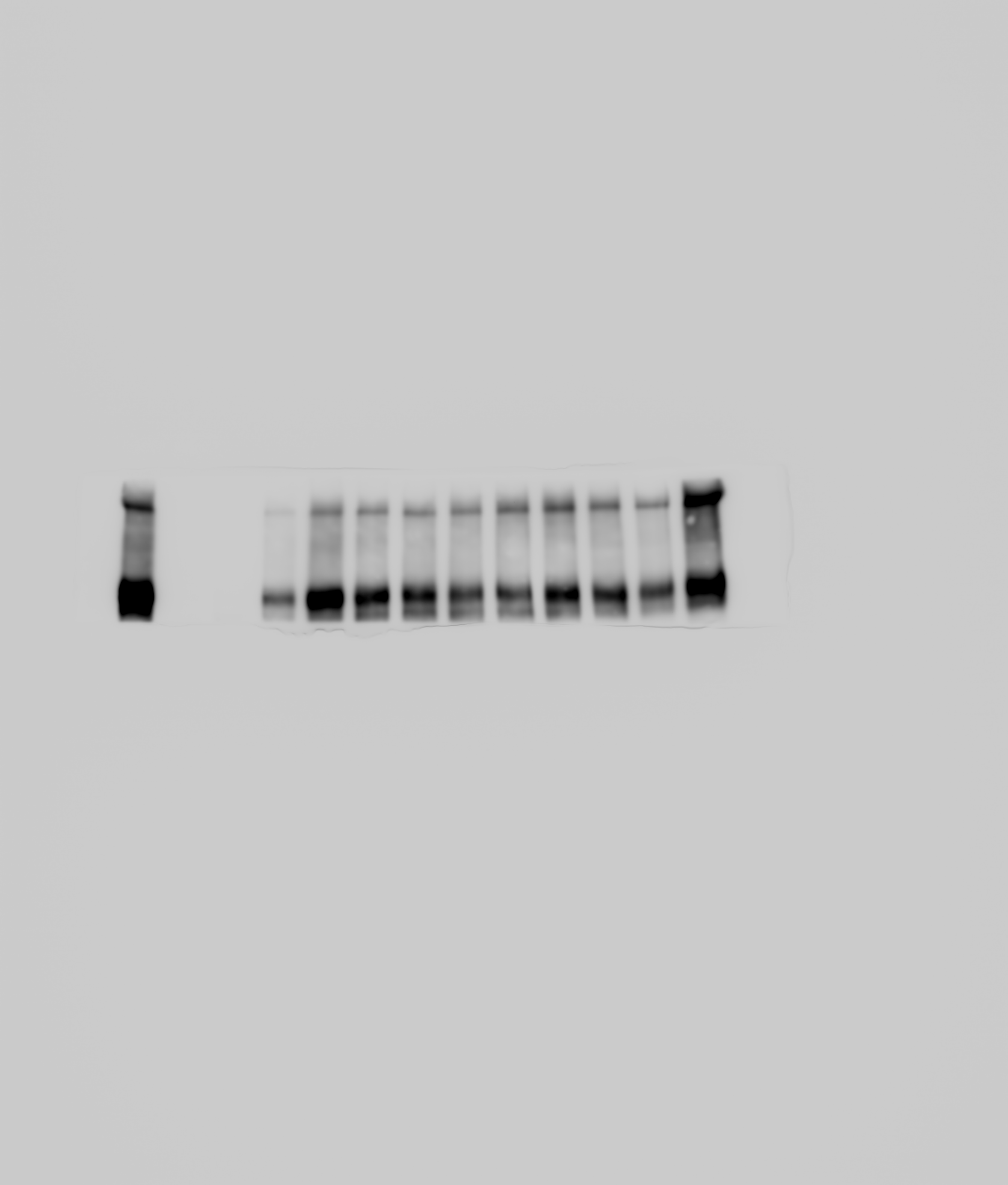

Supplement: Figure 7—figure supplement 1—source data 1. [file elife-86972-fig7-figsupp1-data1.zip › Figure 7-S1E/HA-sample.tif]

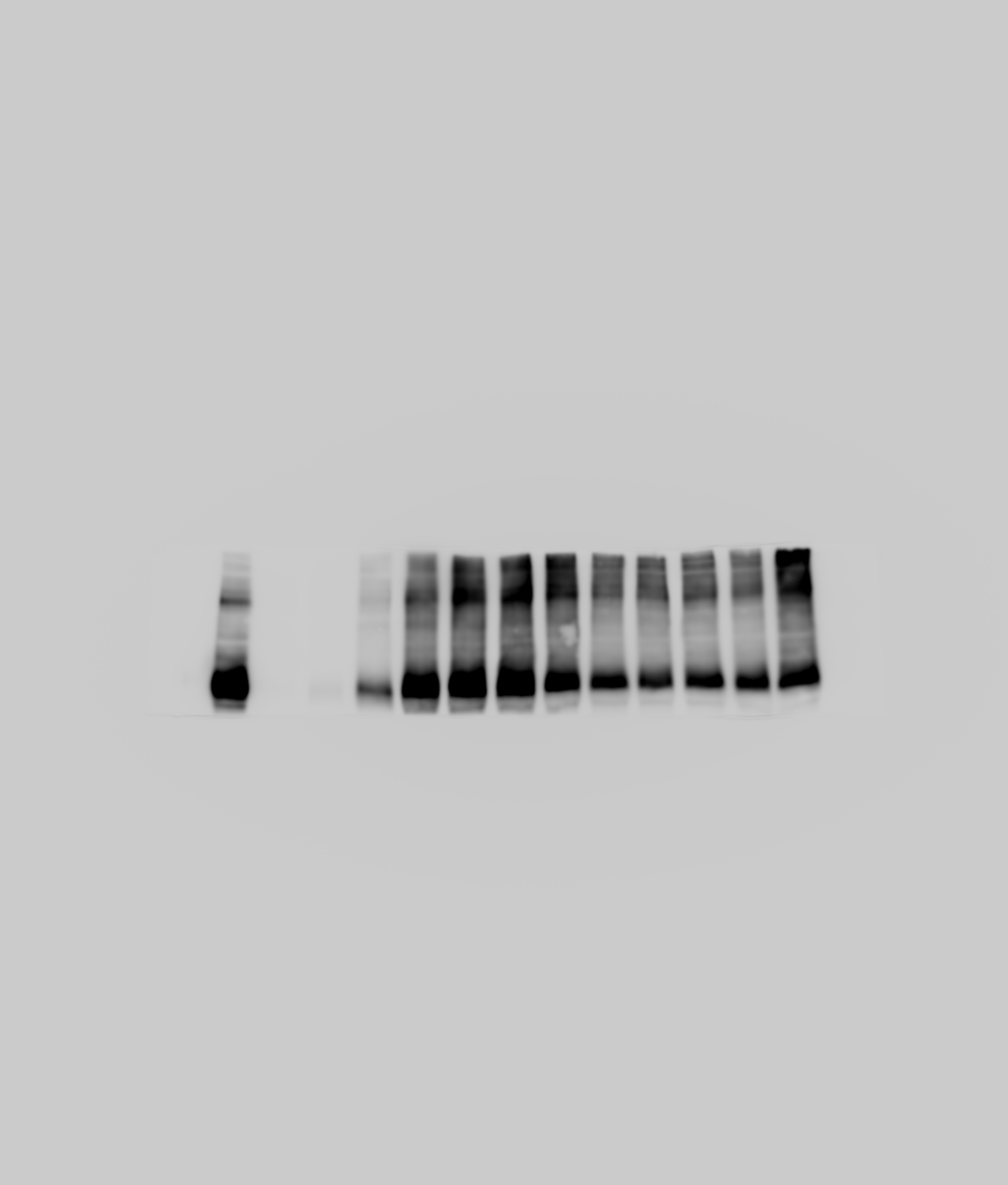

Supplement: Figure 7—figure supplement 1—source data 1. [file elife-86972-fig7-figsupp1-data1.zip › Figure 7-S1E/SV2A.tif]

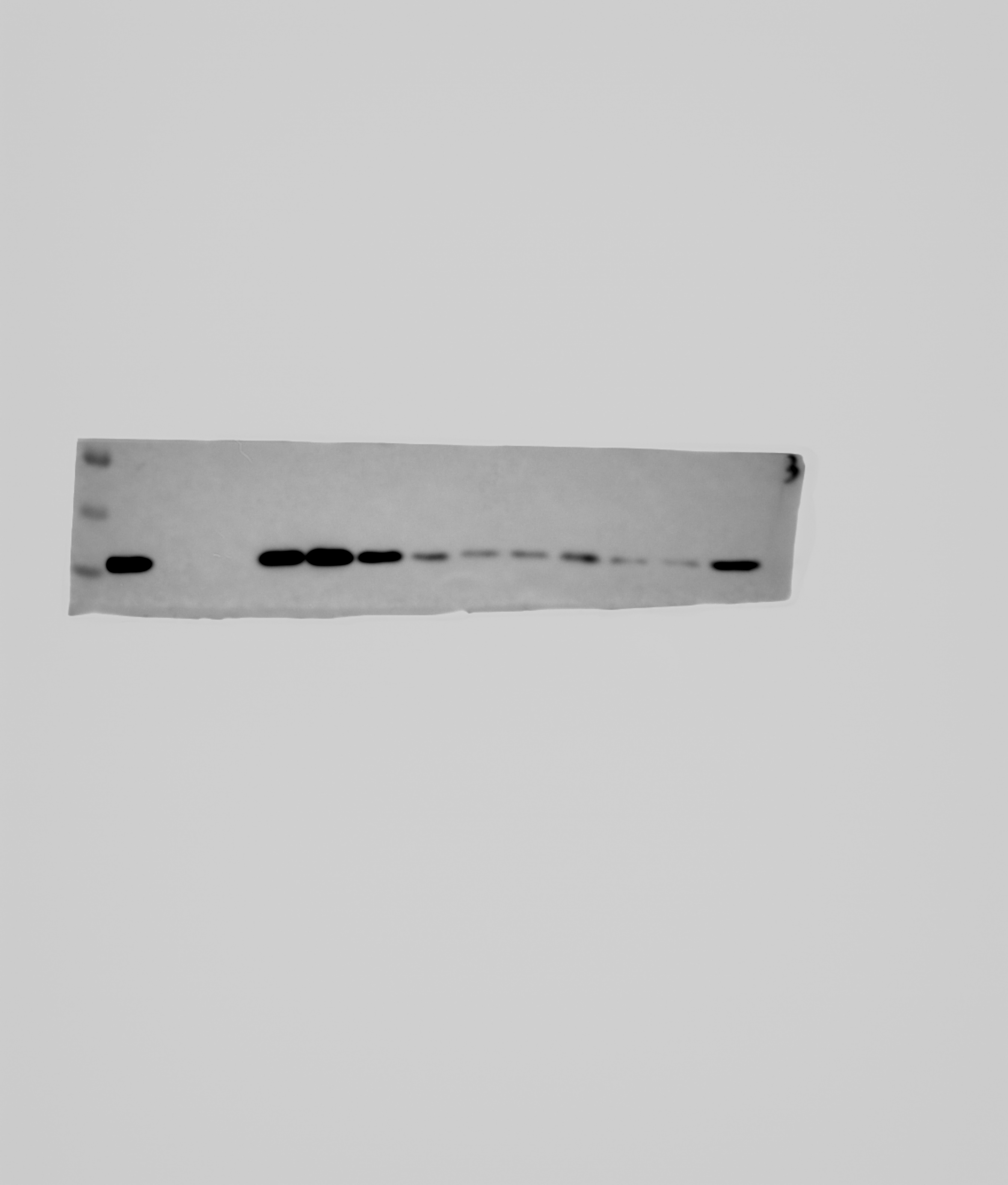

Supplement: Figure 7—figure supplement 1—source data 1. [file elife-86972-fig7-figsupp1-data1.zip › Figure 7-S1E/Syb2.tif]

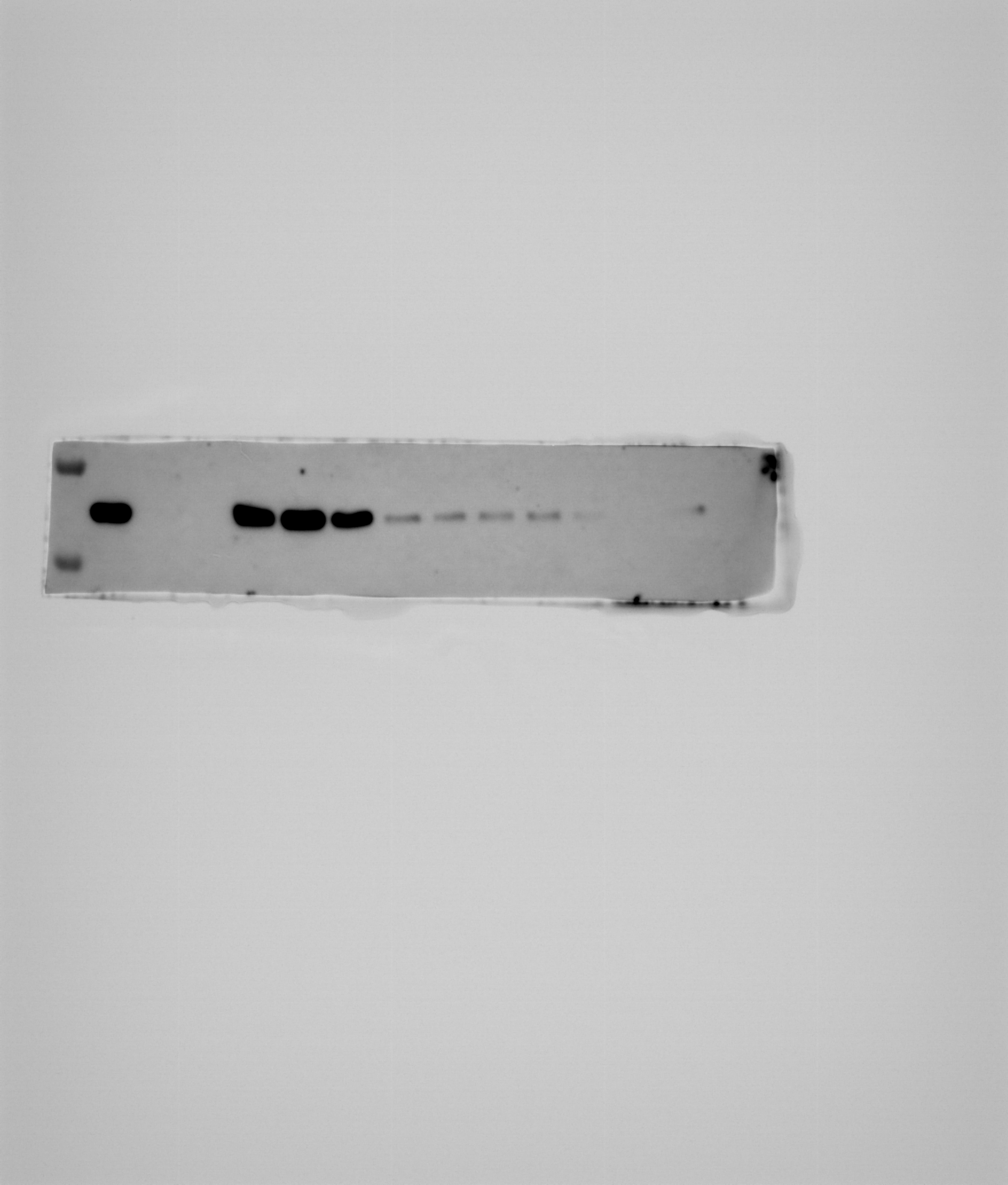

Supplement: Figure 7—figure supplement 1—source data 1. [file elife-86972-fig7-figsupp1-data1.zip › Figure 7-S1E/Syp.tif]

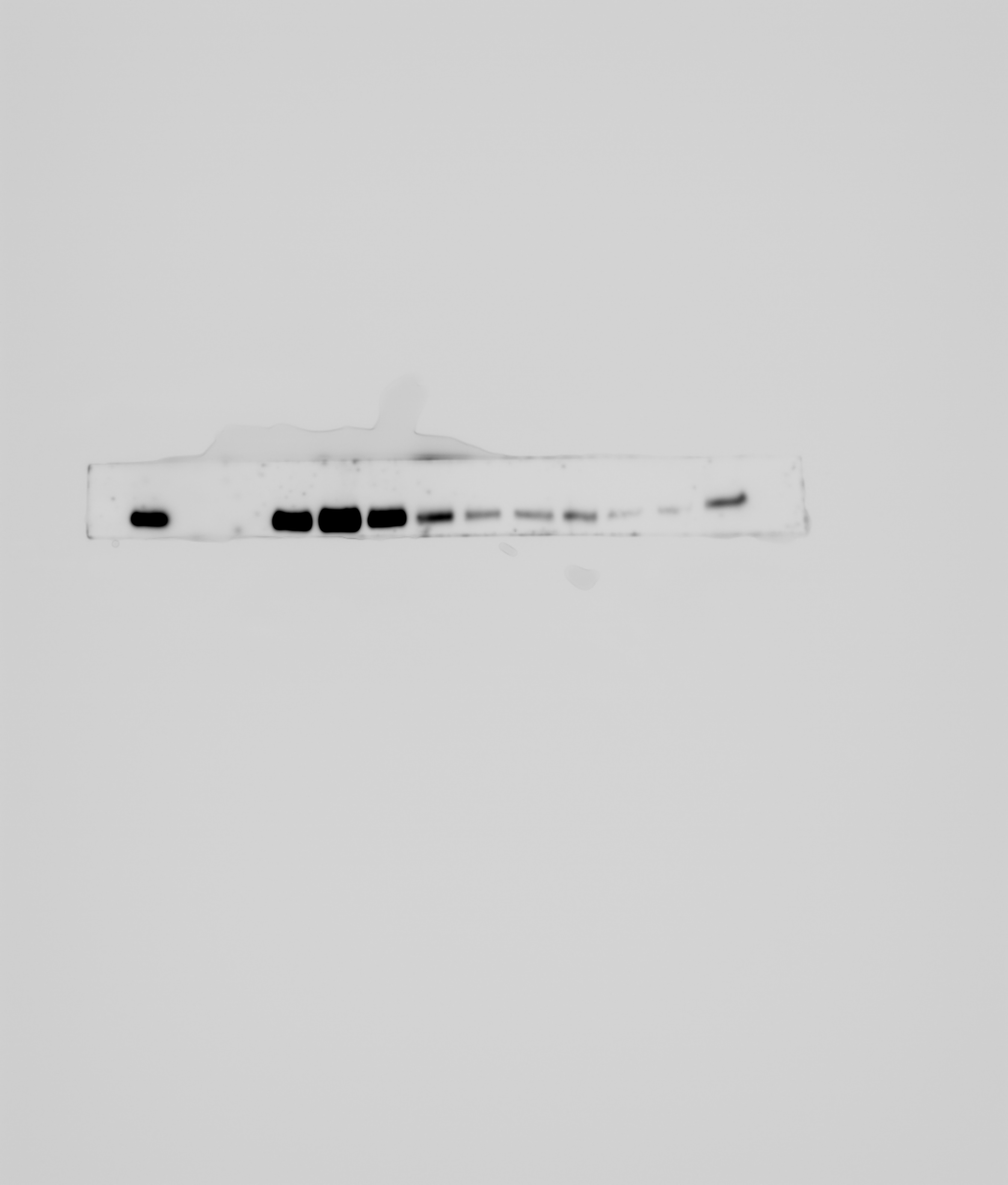

Supplement: Figure 7—figure supplement 1—source data 1. [file elife-86972-fig7-figsupp1-data1.zip › Figure 7-S1E/Syt1.tif]

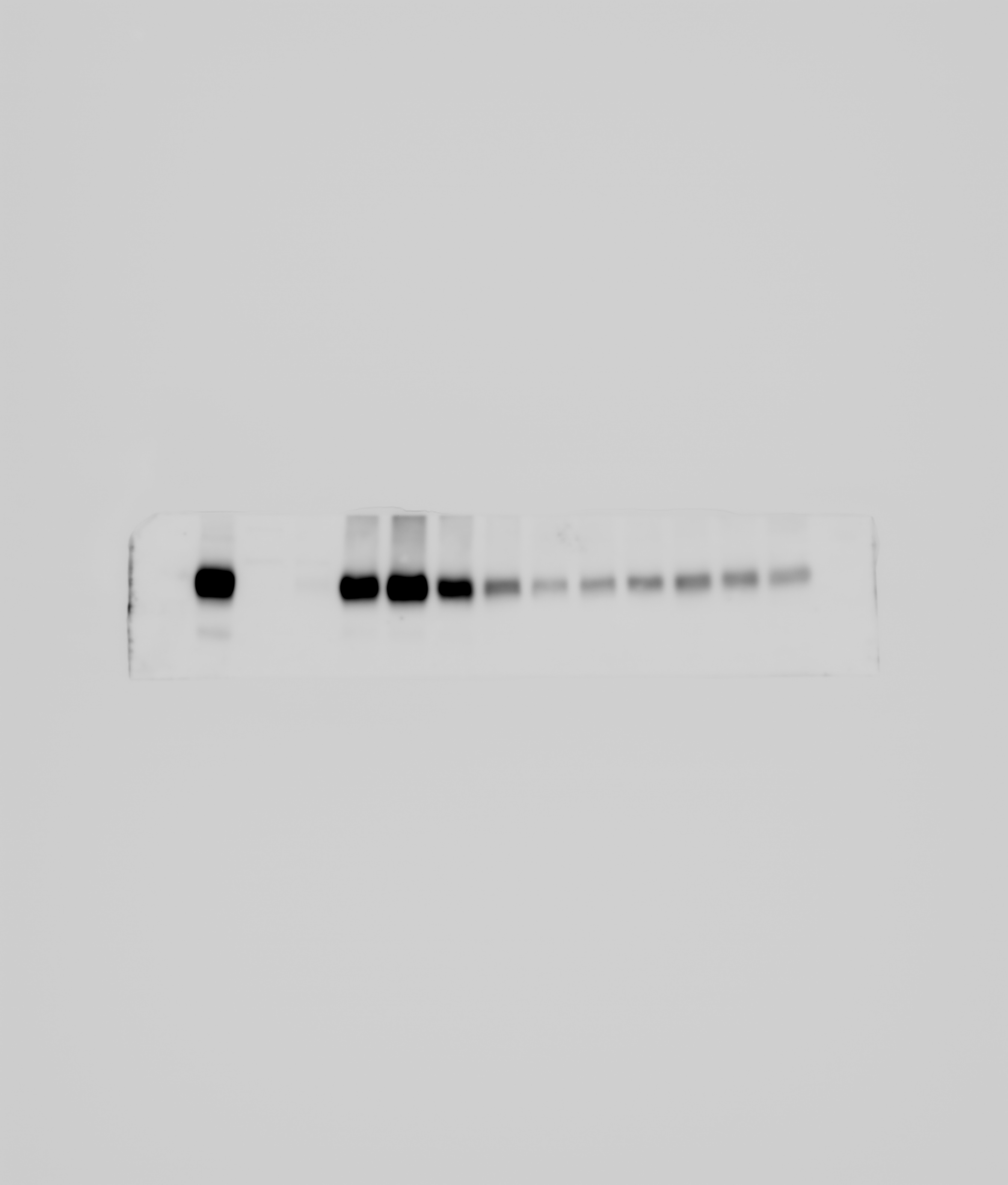

Supplement: Figure 7—figure supplement 1—source data 1. [file elife-86972-fig7-figsupp1-data1.zip › Figure 7-S1E/VGluT1.tif]

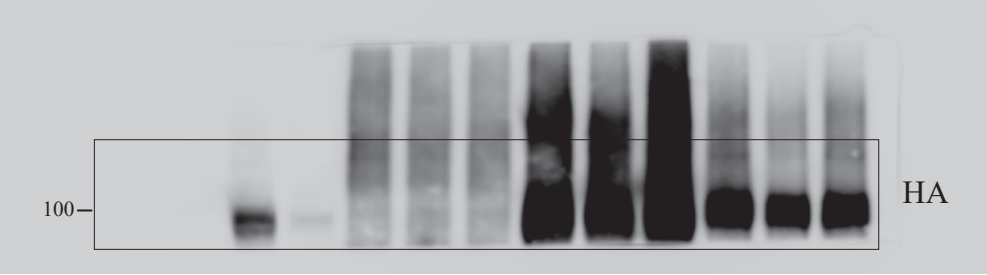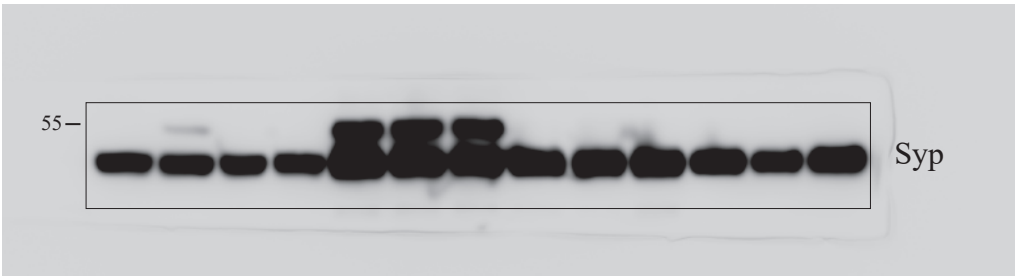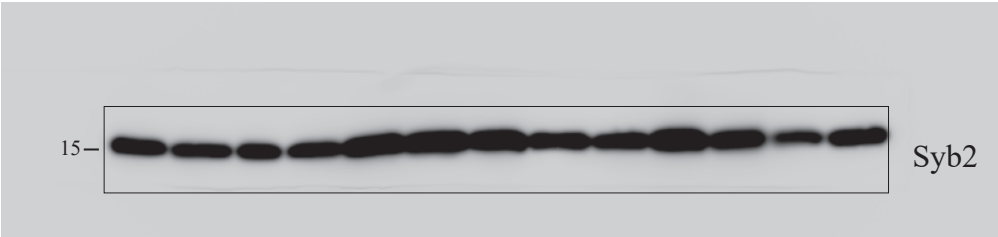

Supplement: Figure 7—figure supplement 1—source data 2. [file elife-86972-fig7-figsupp1-data2.zip › FigureS9A-Source Data-WB.pdf]

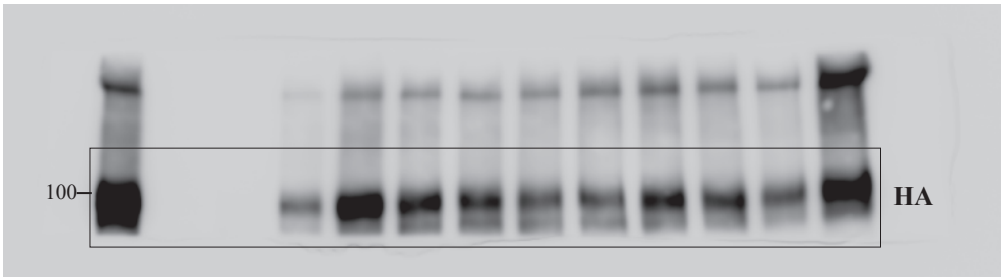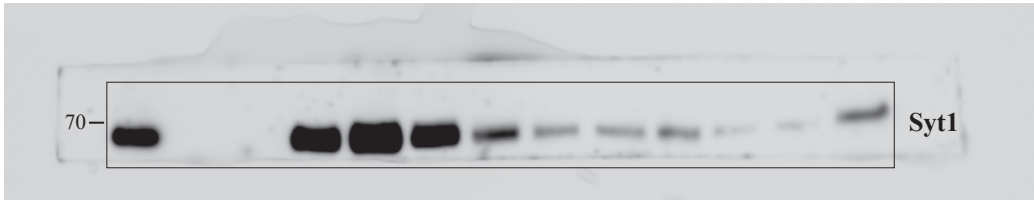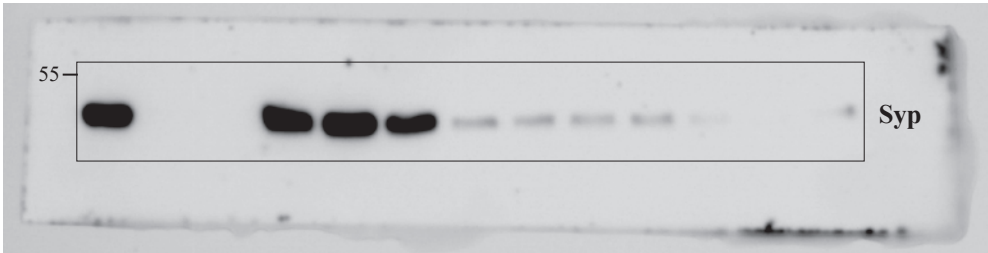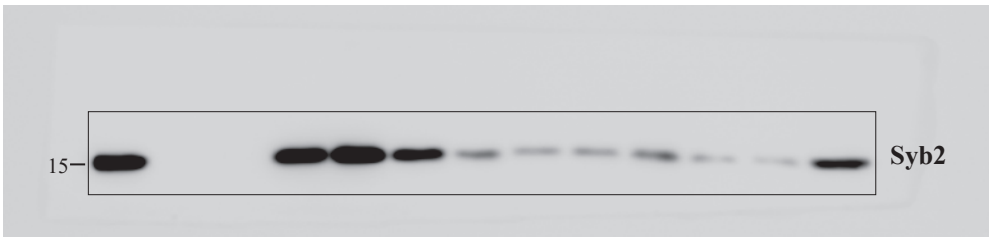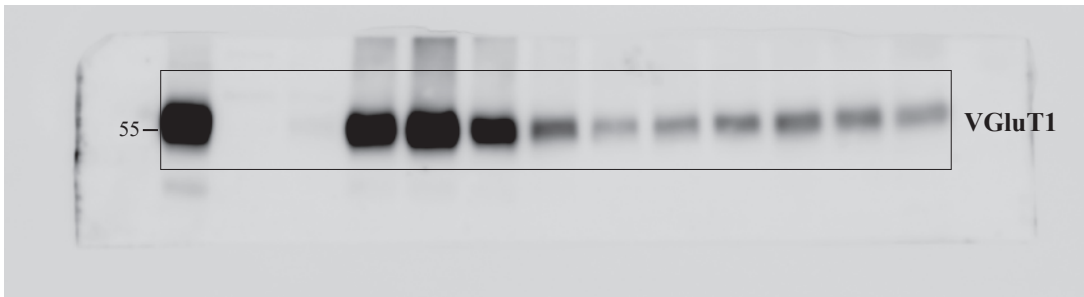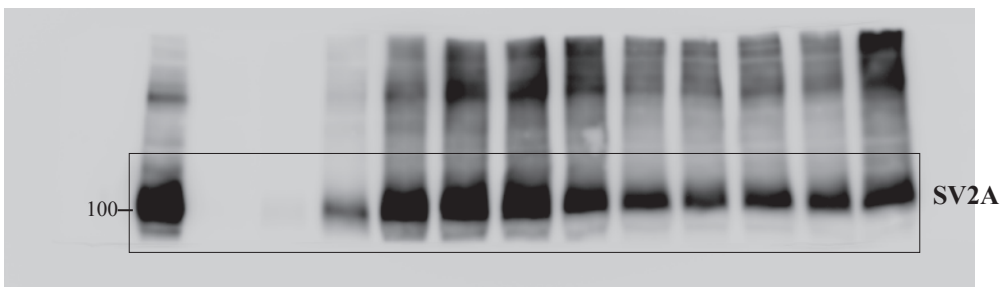

Supplement: Figure 7—figure supplement 1—source data 2. [file elife-86972-fig7-figsupp1-data2.zip › FigureS9E-Source Data-WB.pdf]

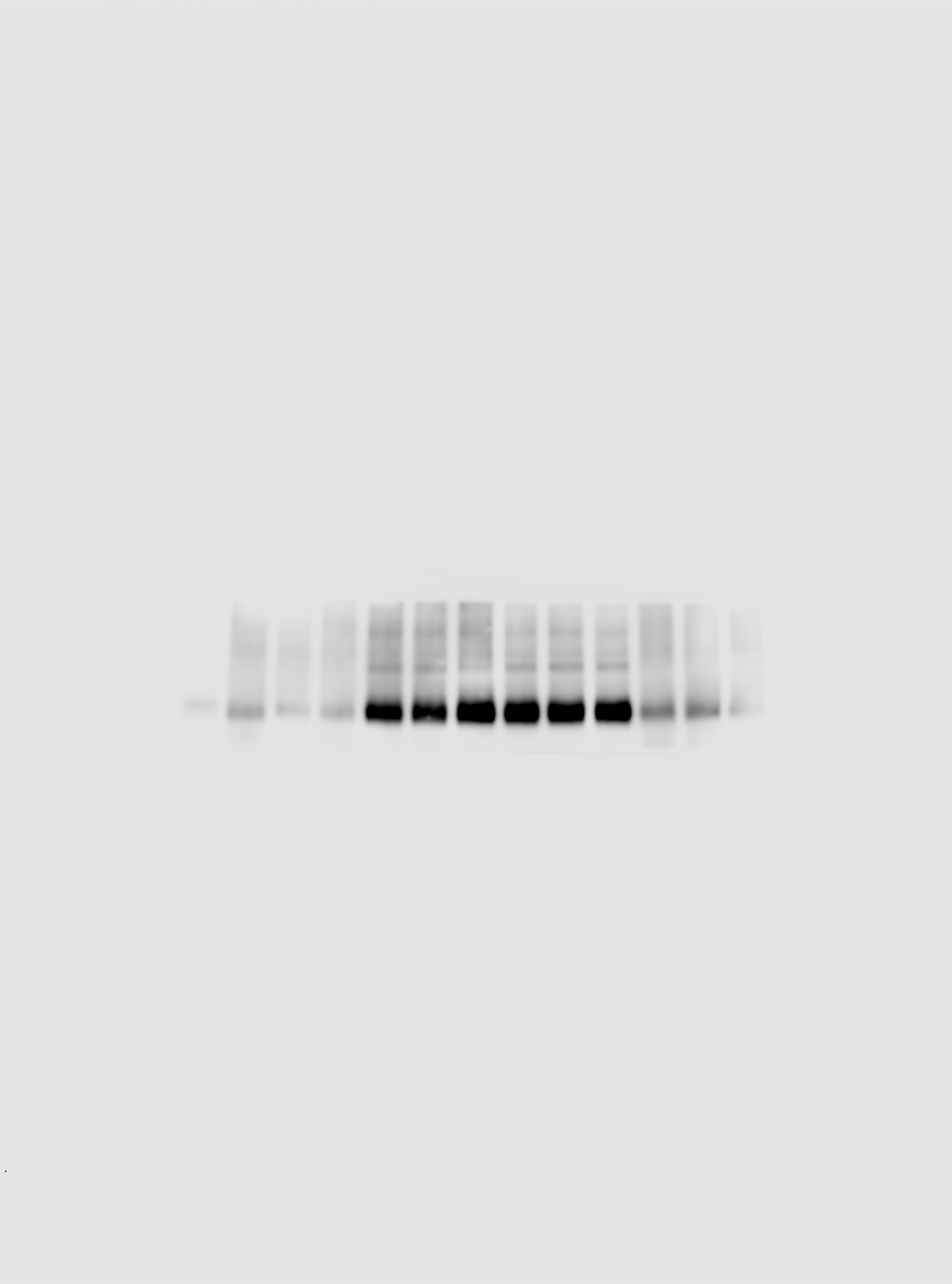

Supplement: Figure 9—source data 2. [file elife-86972-fig9-data2.zip › Figure 9B/HA.tif]

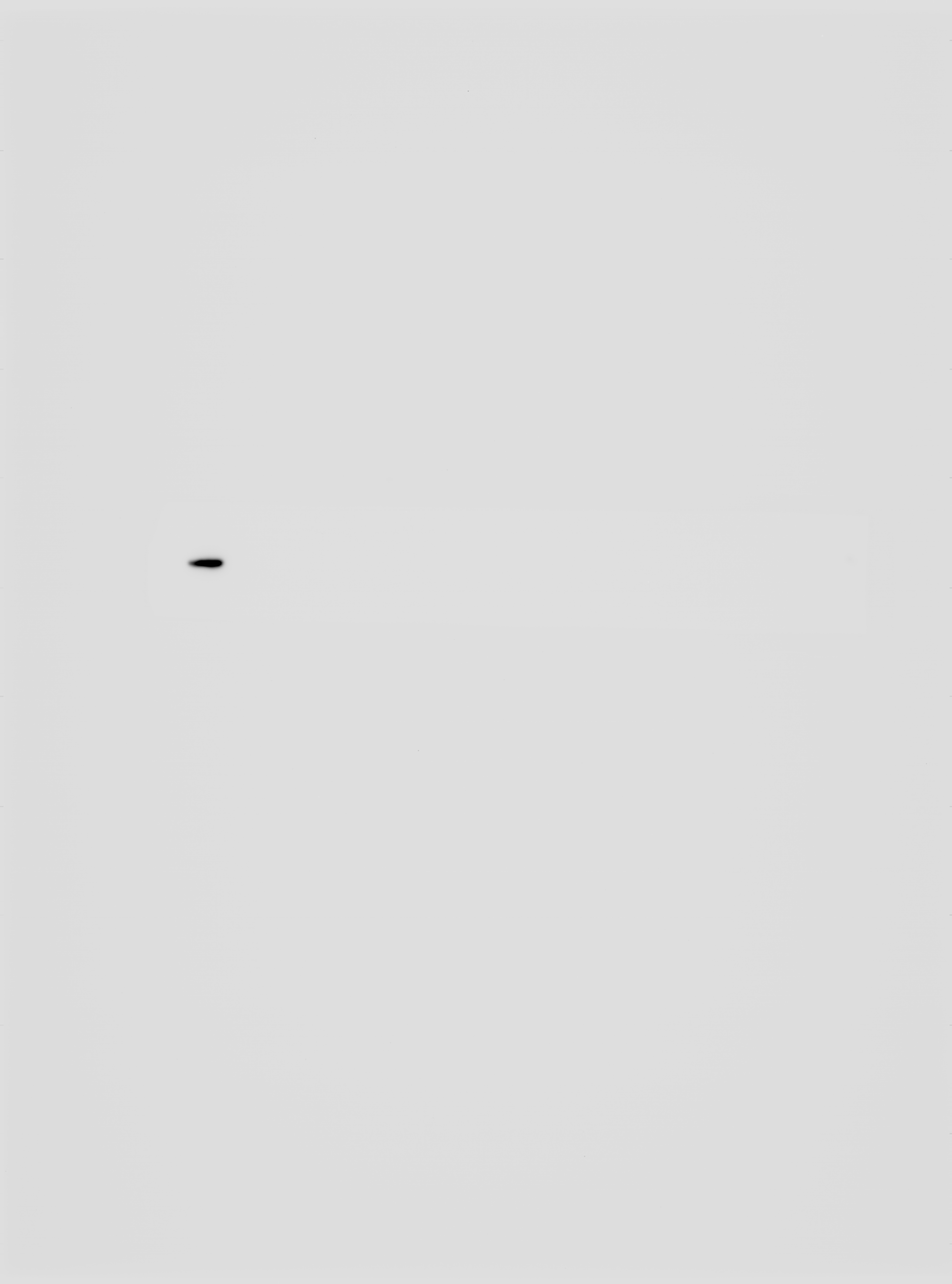

Supplement: Figure 9—source data 2. [file elife-86972-fig9-data2.zip › Figure 9B/LC3B.tif]

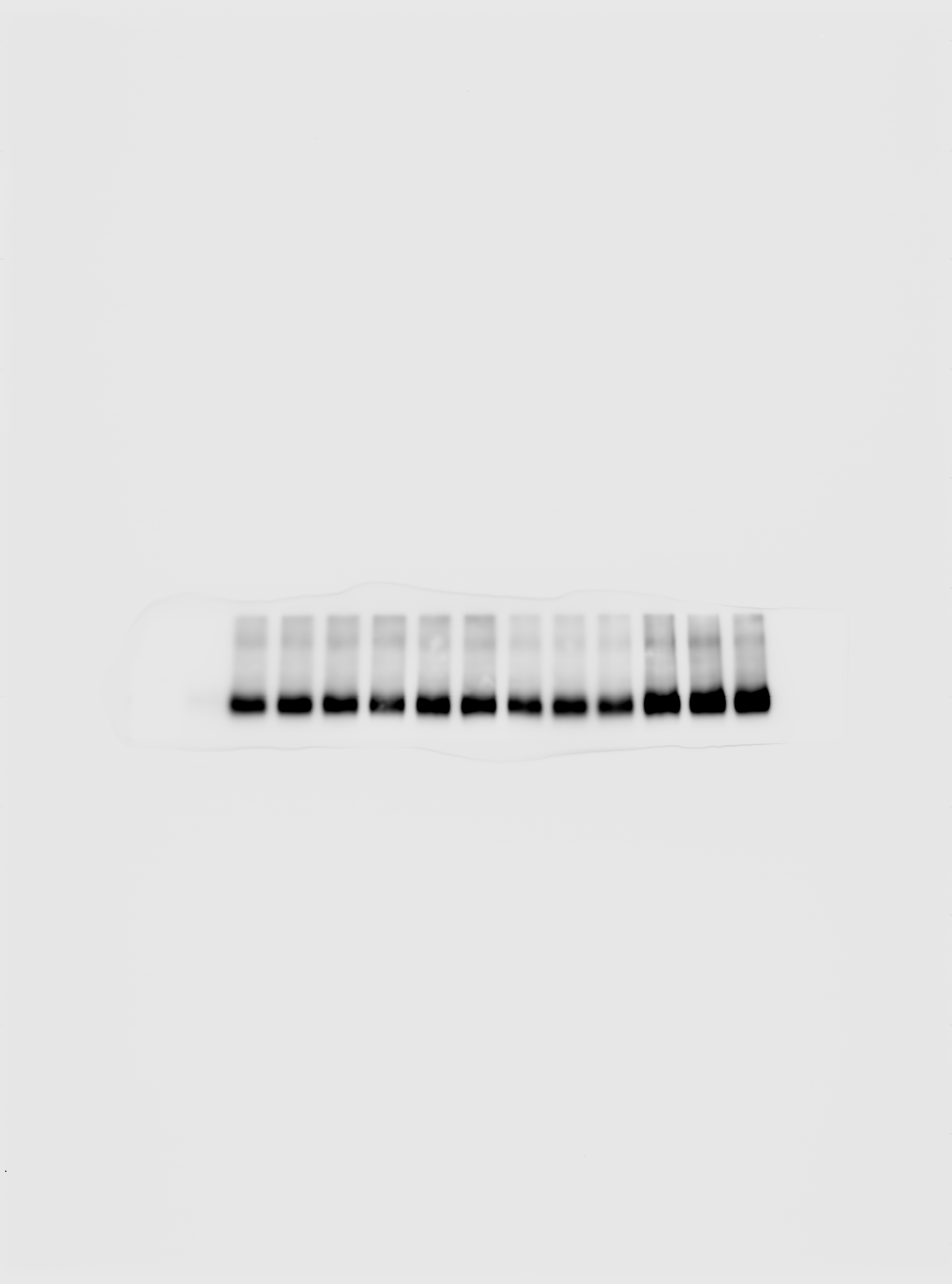

Supplement: Figure 9—source data 2. [file elife-86972-fig9-data2.zip › Figure 9B/SV2A.tif]

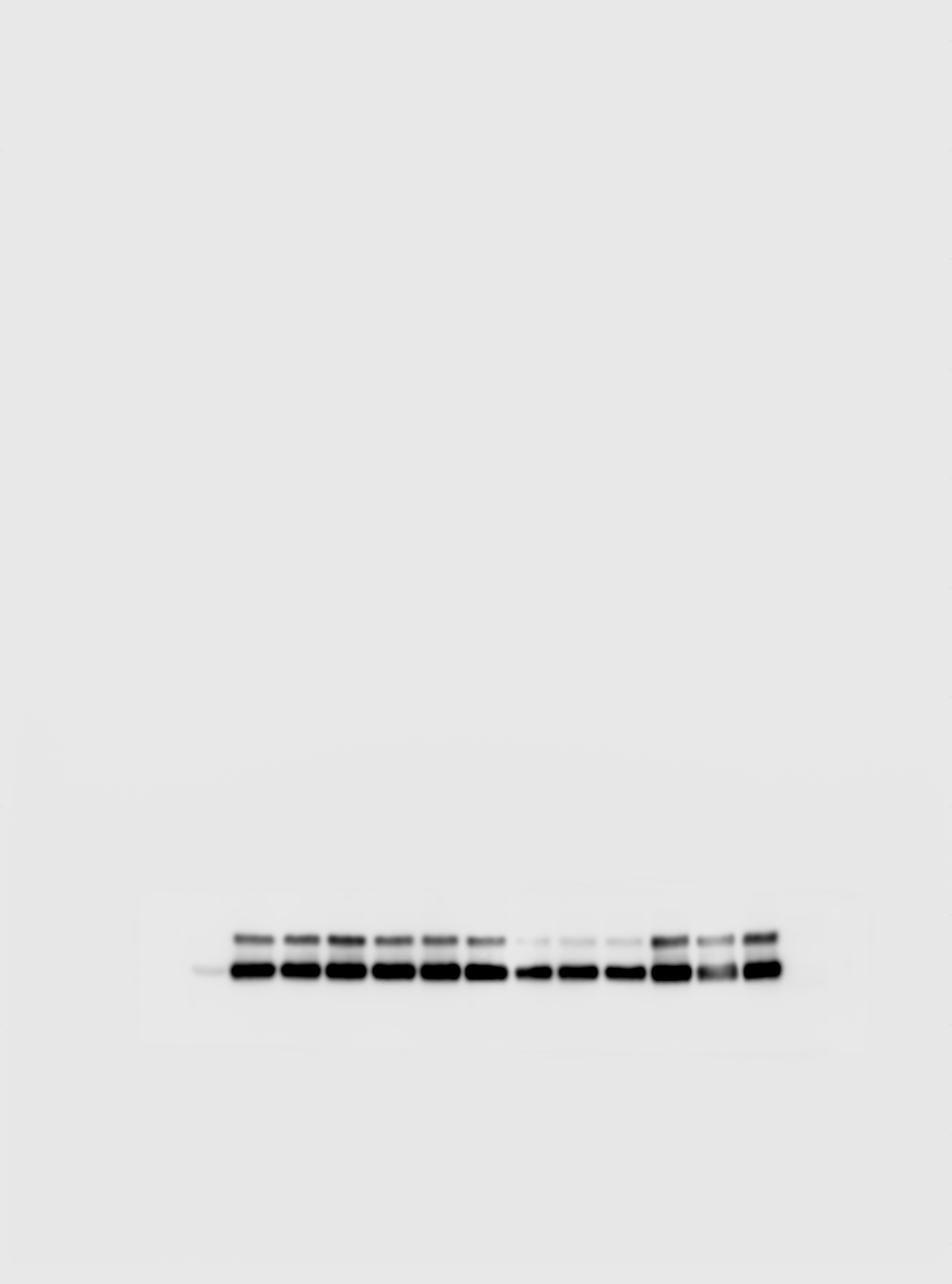

Supplement: Figure 9—source data 2. [file elife-86972-fig9-data2.zip › Figure 9B/Syp.tif]

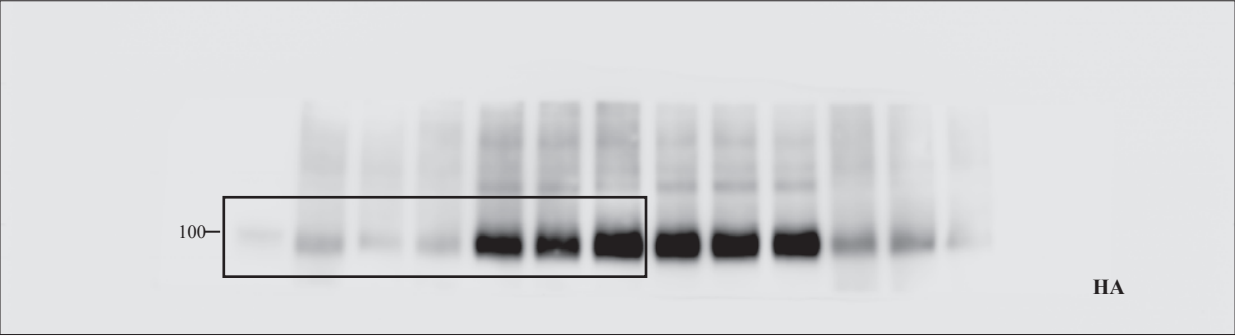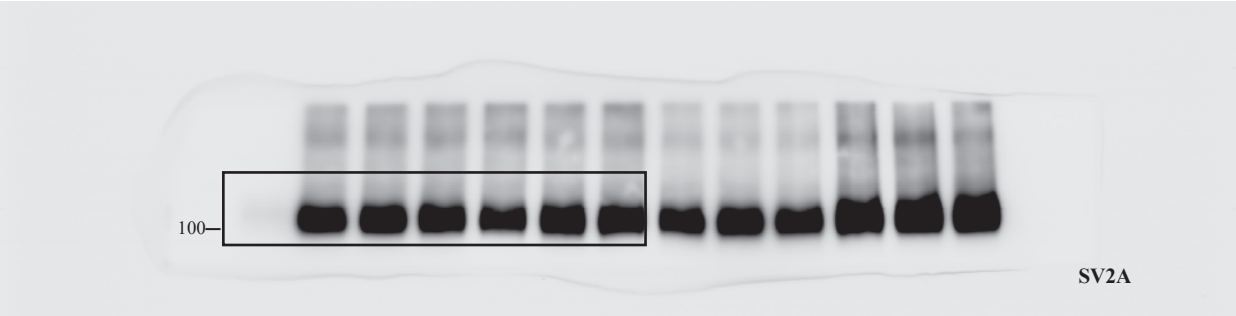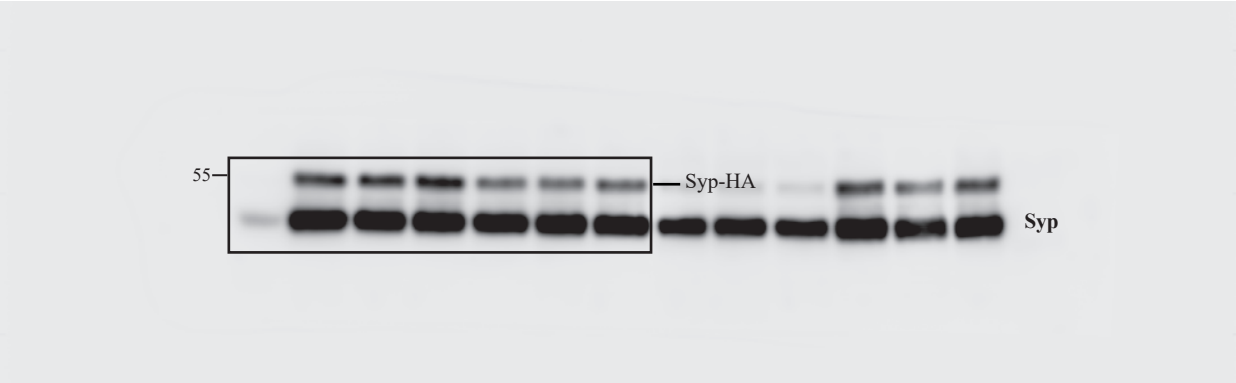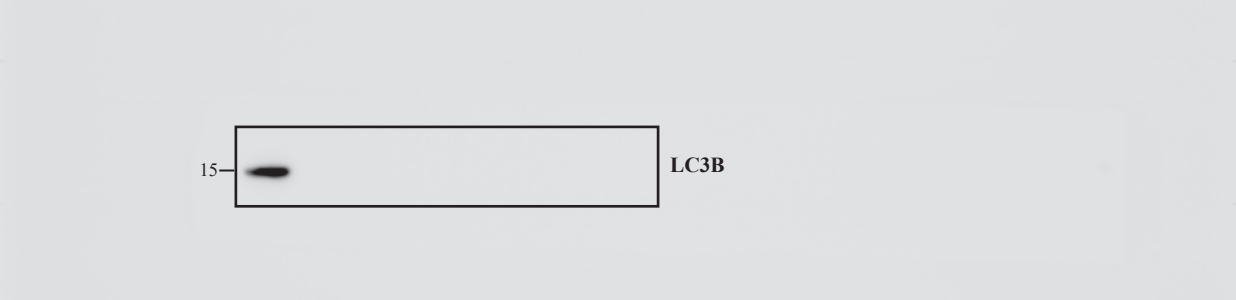

Supplement: Figure 9—source data 3. [file elife-86972-fig9-data3.zip › Figure9B-Source Data-WB.pdf]

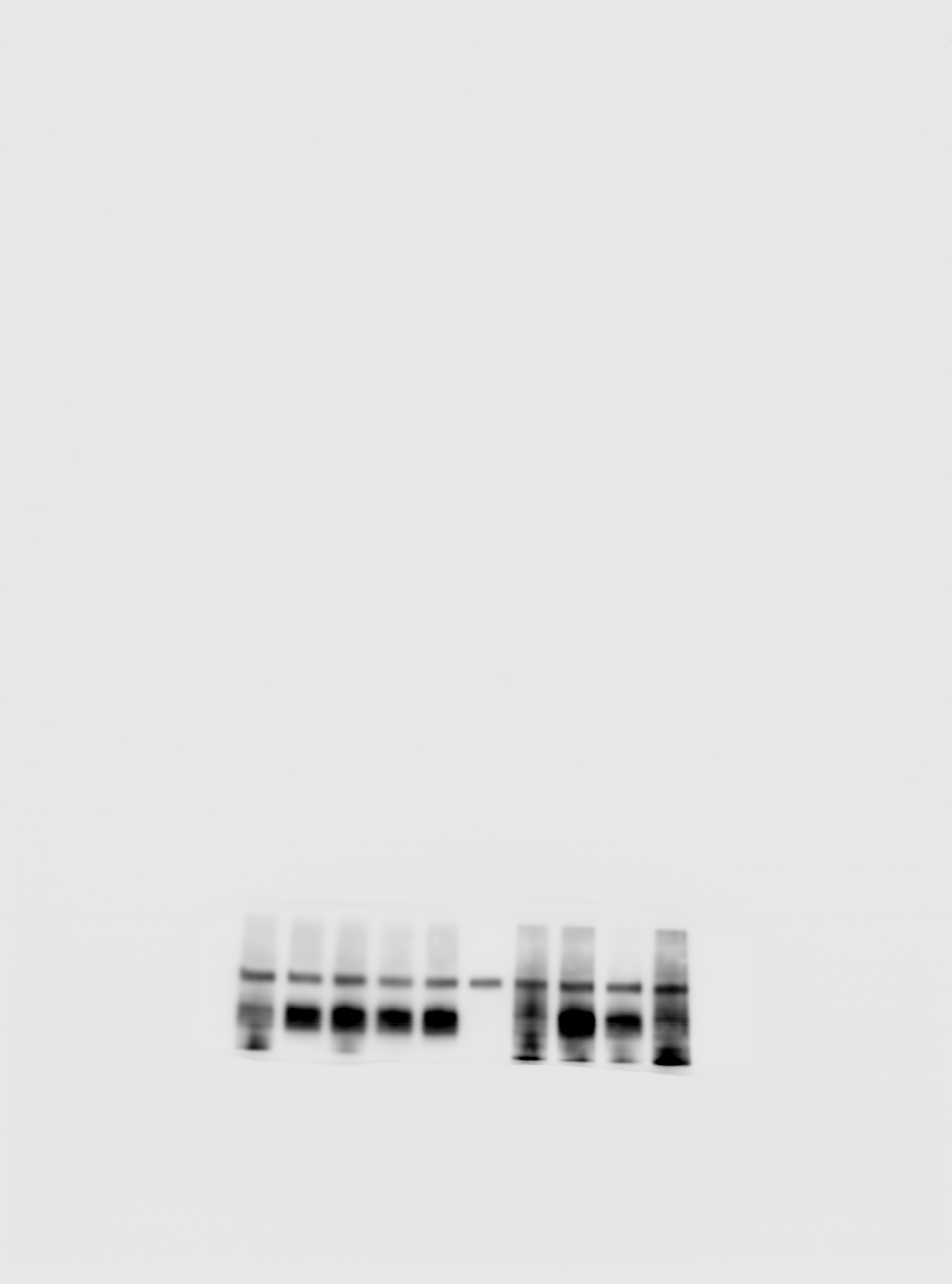

Supplement: Figure 9—figure supplement 1—source data 1. [file elife-86972-fig9-figsupp1-data1.zip › Figure 9-S1B/HA-1.tif]

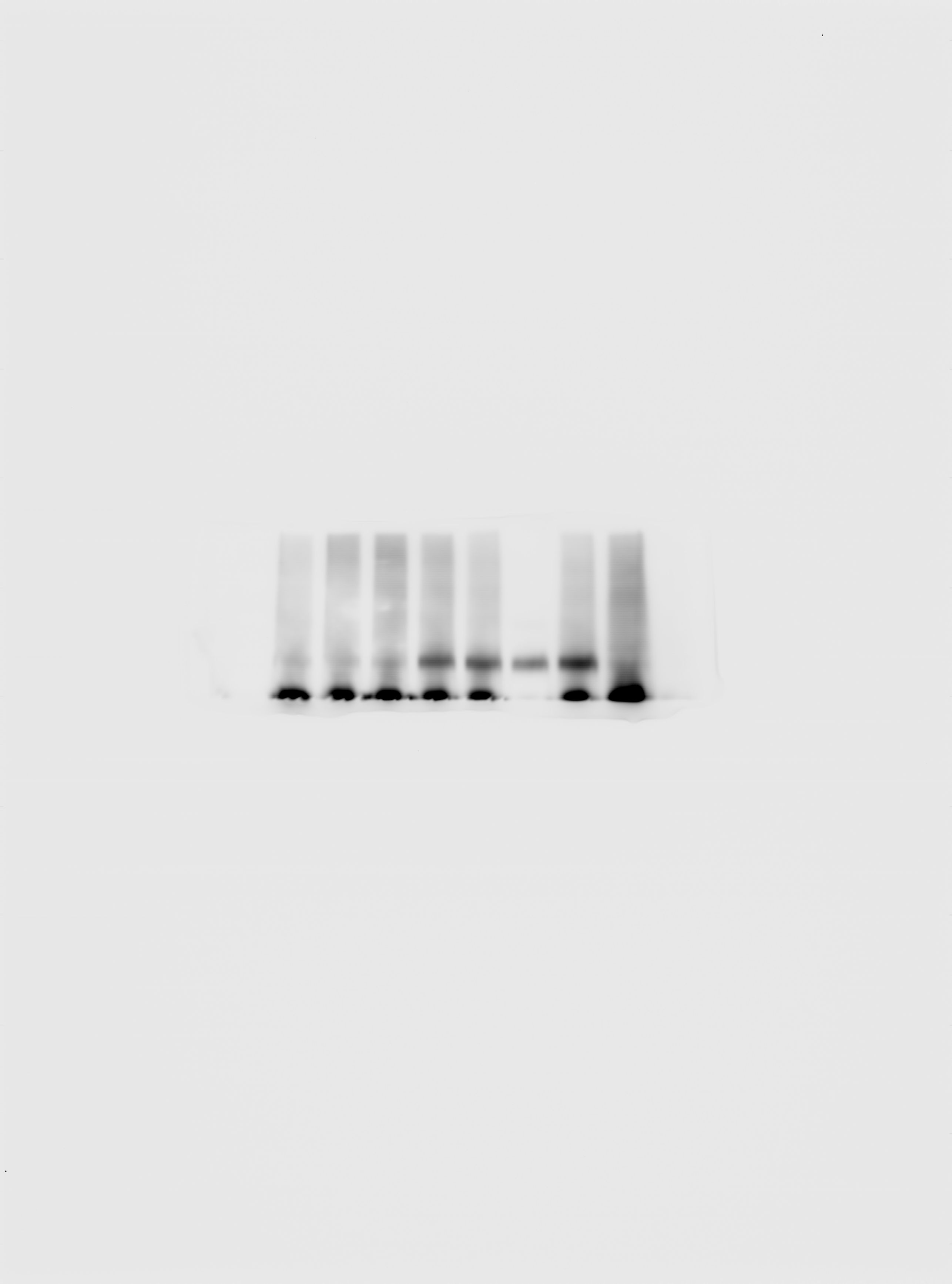

Supplement: Figure 9—figure supplement 1—source data 1. [file elife-86972-fig9-figsupp1-data1.zip › Figure 9-S1B/Input-HA-2.tif]

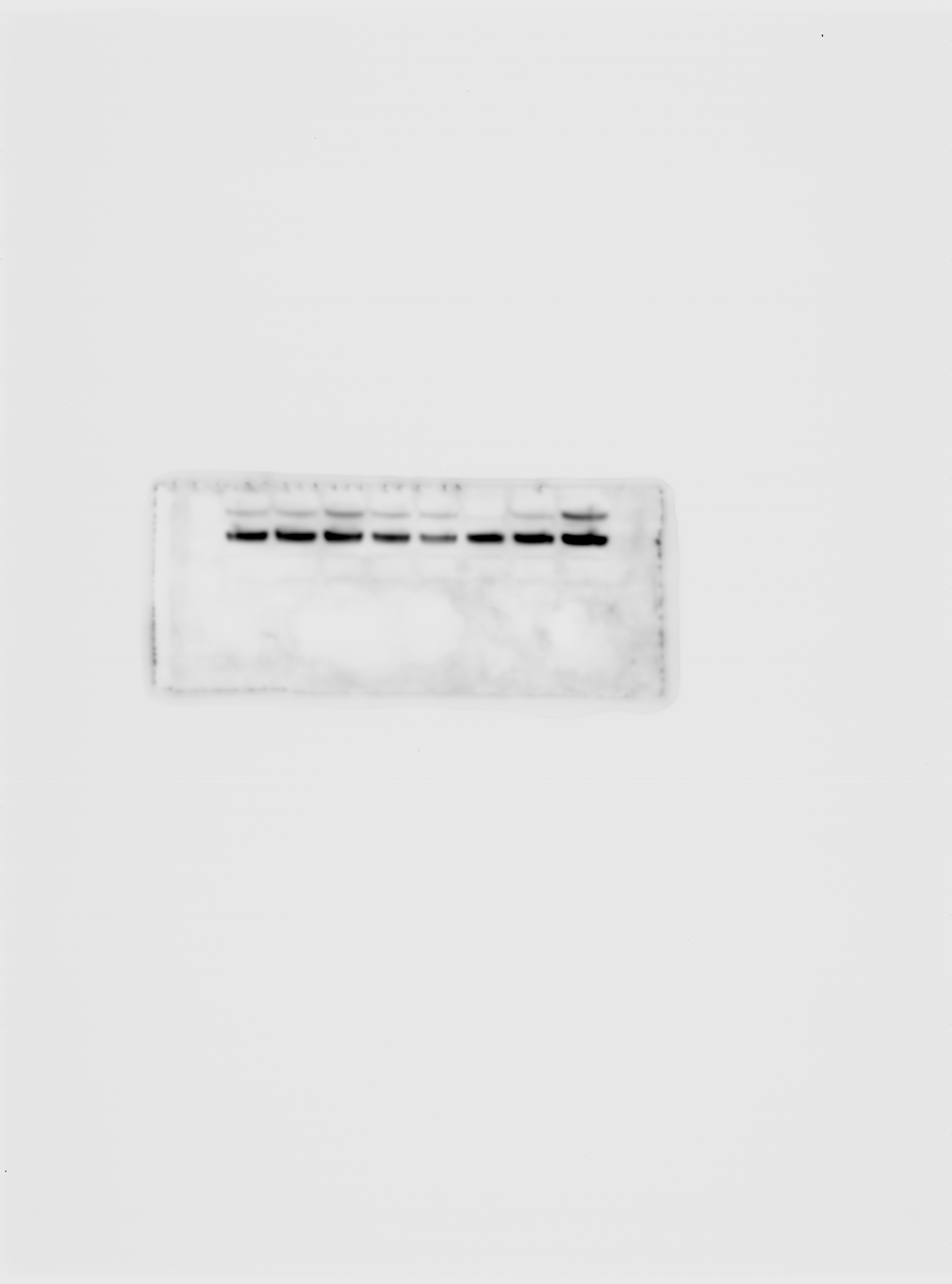

Supplement: Figure 9—figure supplement 1—source data 1. [file elife-86972-fig9-figsupp1-data1.zip › Figure 9-S1B/Input-Syp-2.tif]

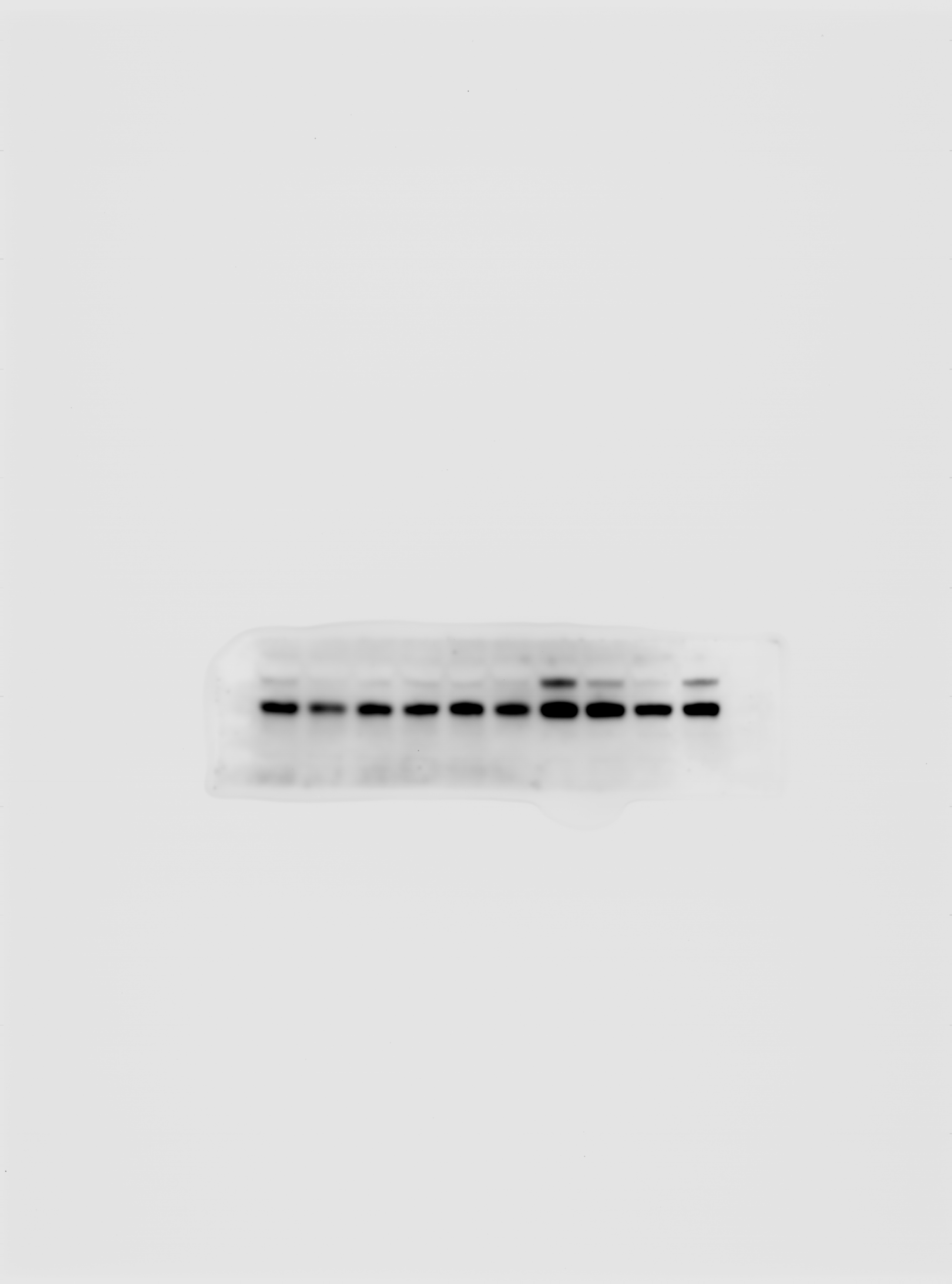

Supplement: Figure 9—figure supplement 1—source data 1. [file elife-86972-fig9-figsupp1-data1.zip › Figure 9-S1B/Syp-1.tif]
