# Supplementary material for: Importance of glutamine in synaptic vesicles revealed by functional studies of SLC6A17 and its mutations pathogenic for intellectual disability
Source: eLife. 2023 Jul 13;12:RP86972. doi: 10.7554/eLife.86972 (PMC10393021; doi:10.7554/eLife.86972)
Supplement: Figure 4—source data 2. [file elife-86972-fig4-data2.zip › Figure4C-Source Data-WB.pdf]

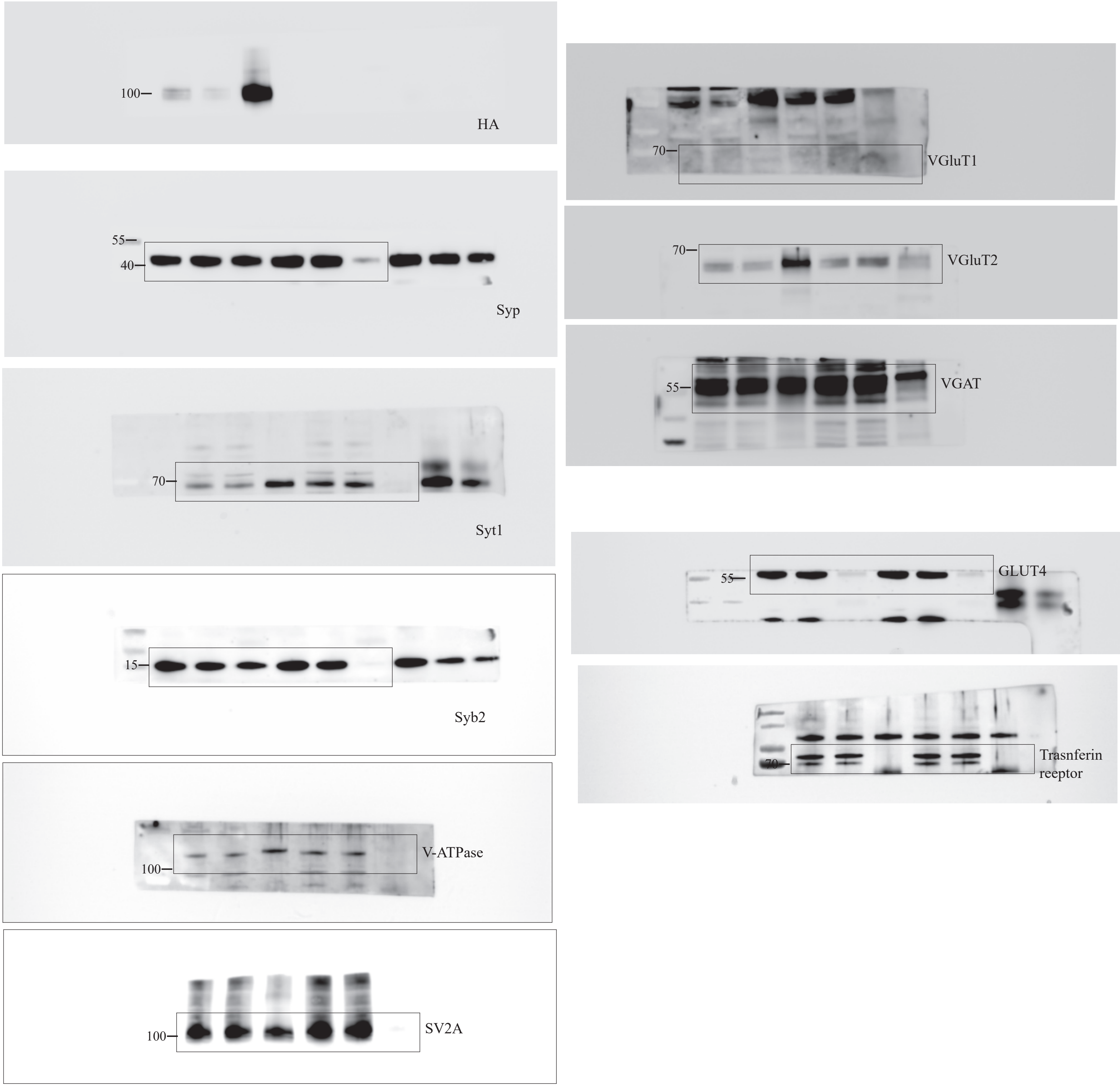
100
HA
55
40
Syp
70
Syt1
15
Syb2
100
V-ATPase
100
SV2A
70
VGluT1
70
VGluT2
55
VGAT
55
GLUT4
70
Trasnferin
reeptor
