# Supplementary material for: Importance of glutamine in synaptic vesicles revealed by functional studies of SLC6A17 and its mutations pathogenic for intellectual disability
Source: eLife. 2023 Jul 13;12:RP86972. doi: 10.7554/eLife.86972 (PMC10393021; doi:10.7554/eLife.86972)

pAAV-hSyn-DIO-Syp-HA-U6-6A17-sgRNA sequence

cctgcaggcagctgcgcgctcgctcgctcactgaggccgcccgggcgtcgggcgacctttggtcgcccggcctcagtgagcgagcgagcgcgcagagagggagtggccaactccatcactaggggttcctgcggcctctagaATGAGGCGGGGTGGGGGTGCCTACCTGACGACCGACCCCGACCCACTGGACAAGCACCCAACCCCCATTCCCCAAATTGCGCATCCCCTATCAGAGAGGGGGAGGGGAAACAGGATGCGGCGAGGCGCGTGCGCACTGCCAGCTTCAGCACCGCGGACAGTGCCTTCGCCCCCGCCTGGCGGCGCGCGCCACCGCCGCCTCAGCACTGAAGGCGCGCTGACGTCACTCGCCGGTCCCCCGCAAACTCCCCTTCCCGGCCACCTTGGTCGCGTCCGCGCCGCCGCCGGCCCAGCCGGACCGCACCACGCGAGGCGCGAGATAGGGGGGCACGGGCGCGACCATCTGCGCTGCGGCGCCGGCGACTCAGCGCTGCCTCAGTCTGCGGTGGGCAGCGGAGGAGTCGTGTCGTGCCTGAGAGCGCAGTCGAGAAACCGGTGCCACCgagctctctggctaactaccggtataacttcgtataggatactttatacgaagttatgcagaatggtagctggattgtagctgctattagcaatatgaaacctcttaataacttcgtatagcatacattatacgaagttatggcgcgccttaAGCGTAATCTGGAACGTCATATGGATAGGATCCTGCATAGTCCGGGACGTCATACGGATAGCCCGCATAGTCAGGAACATCGTATGGGTAgctaccaccgccgccagaaccgccaccgccgctgccaccgccaccagagccgccaccacccatctgattggagaaggaggtgggcgcaccctgttggccgtagccttgctgcccatagtcgccctgaggcccgtagccaccgccaccgccgctggctggctgcccgtaatcgggttgataaccaccctgaggcccgtaggagtcctggggcccatagcctccggggccctgcccatagcccgcatcgccgtaggcatcgccaggagctggttgcttttctggggcgcctggaggtgcgcgcatgaatggggcggcccagcctgtctccttgaacacgaaccataggttgccaacccagagcaccaggttcaggaagccaaacaccaccgaggtgttgagtcctgaagtcacagggtccctcagttccttgcatgtgtttcctgtctggcggcacataggcatctccttgataatgttctctgggtcagtggccatcttcacatcggacaggcctttggcccaggcggatgagctaactagccacatgaaagcgaacactgctgtggccaggaagtccatcattggccctttgttgttctctcggtacttgttctgcaggaagatgtaggtggccagggcccccatggagtagaggaaggcaaacacagccacggtgacaaagaattcagccgaggaggagtagtcaccaactaggaagatcttggtagtgccccctttaacgcaggagggtgcatcaaagtacacttggtgcagcctgaatgggtactcaaattcgacttcgatgttgagggcactctccgtcttgttggcacactccacgctcagccgaagctctccggtgtagctgccgcacgtagcaaaggcgaagatggcaaagacccactgcagcaccttcacgaagccaaggggctccttgaccacccggaactgacccccagccaccagctgattcaccacgtccatgtctgccagcagcagcattggctagcataacttcgtataaagtatcctatacgaagttatttgccttaacccagaaattatcactgttattctttagaatggtgcaaagaataacttcgtataatgtatgctatacgaagttatgaattcgatatcaagcttatcgataatcaacctctggattacaaaatttgtgaaagattgactggtattcttaactatgttgctccttttacgctatgtggatacgctgctttaatgcctttgtatcatgctattgcttcccgtatggctttcattttctcctccttgtataaatcctggttgctgtctctttatgaggagttgtggcccgttgtcaggcaacgtggcgtggtgtgcactgtgtttgctgacgcaacccccactggttggggcattgccaccacctgtcagctcctttccgggactttcgctttccccctccctattgccacggcggaactcatcgccgcctgccttgcccgctgctggacaggggctcggctgttgggcactgacaattccgtggtgttgtcggggaaatcatcgtcctttccttggctgctcgcctatgttgccacctggattctgcgcgggacgtccttctgctacgtcccttcggccctcaatccagcggaccttccttcccgcggcctgctgccggctctgcggcctcttccgcgtcttcgccttcgccctcagacgagtcggatctccctttgggccgcctccccgcatcgataccgagcgctgctcgagagatctacgggtggcatccctgtgacccctccccagtgcctctcctggccctggaagttgccactccagtgcccaccagccttgtcctaataaaattaagttgcatcattttgtctgactaggtgtccttctataatattatggggtggaggggggtggtatggagcaaggggcaagttgggaagacaacctgtagggcctgcggggtctattgggaaccaagctggagtgcagtggcacaatcttggctcactgcaatctccgcctcctgggttcaagcgattctcctgcctcagcctcccgagttgttgggattccaggcatgcatgaccaggctcagctaatttttgtttttttggtagagacggggtttcaccatattggccaggctggtctccaactcctaatctcaggtgatctacccaccttggcctcccaaattgctgggattacaggcgtgaaccactgctcccttccctgtccttggtaccgagggcctatttcccatgattccttcatatttgcatatacgatacaaggctgttagagagataattggaattaatttgactgtaaacacaaagatattagtacaaaatacgtgacgtagaaagtaataatttcttgggtagtttgcagttttaaaattatgttttaaaatggactatcatatgcttaccgtaacttgaaagtatttcgatttcttggctttatatatcttgtggaaaggacgaaacaccgCGATGCTCCAGGCCACAAGGgtttaagagctatgctggaaacagcatagcaagtttaaataaggctagtccgttatcaacttgaaaaagtggcaccgagtcggtgcaacaaagcaccagtggtctagtggtagaatagtaccctgccacggtacagacccgggttcgattcccggctggtgcaGCCGTGGCAGCATTGGTGTGgtttaagagctatgctggaaacagcatagcaagtttaaataaggctagtccgttatcaacttgaaaaagtggcaccgagtcggtgcaacaaagcaccagtggtctagtggtagaatagtaccctgccacggtacagacccgggttcgattcccggctggtgcaTGGGCCTGGGCAACATCTGGgtttaagagctatgctggaaacagcatagcaagtttaaataaggctagtccgttatcaacttgaaaaagtggcaccgagtcggtgcaacaaagcaccagtggtctagtggtagaatagtaccctgccacggtacagacccgggttcgattcccggctggtgcatttttgggcccgcggccgcaggaacccctagtgatggagttggccactccctctctgcgcgctcgctcgctcactgaggccgggcgaccaaaggtcgcccgacgcccgggctttgcccgggcggcctcagtgagcgagcgagcgcgcagctgcctgcaggggcgcctgatgcggtattttctccttacgcatctgtgcggtatttcacaccgcatacgtcaaagcaaccatagtacgcgccctgtagcggcgcattaagcgcggcgggtgtggtggttacgcgcagcgtgaccgctacacttgccagcgccttagcgcccgctcctttcgctttcttcccttcctttctcgccacgttcgccggctttccccgtcaagctctaaatcgggggctccctttagggttccgatttagtgctttacggcacctcgaccccaaaaaacttgatttgggtgatggttcacgtagtgggccatcgccctgatagacggtttttcgccctttgacgttggagtccacgttctttaatagtggactcttgttccaaactggaacaacactcaactctatctcgggctattcttttgatttataagggattttgccgatttcggtctattggttaaaaaatgagctgatttaacaaaaatttaacgcgaattttaacaaaatattaacgtttacaattttatggtgcactctcagtacaatctgctctgatgccgcatagttaagccagccccgacacccgccaacacccgctgacgcgccctgacgggcttgtctgctcccggcatccgcttacagacaagctgtgaccgtctccgggagctgcatgtgtcagaggttttcaccgtcatcaccgaaacgcgcgagacgaaagggcctcgtgatacgcctatttttataggttaatgtcatgataataatggtttcttagacgtcaggtggcacttttcggggaaatgtgcgcggaacccctatttgtttatttttctaaatacattcaaatatgtatccgctcatgagacaataaccctgataaatgcttcaataatattgaaaaaggaagagtatgagtattcaacatttccgtgtcgcccttattcccttttttgcggcattttgccttcctgtttttgctcacccagaaacgctggtgaaagtaaaagatgctgaagatcagttgggtgcacgagtgggttacatcgaactggatctcaacagcggtaagatccttgagagttttcgccccgaagaacgttttccaatgatgagcacttttaaagttctgctatgtggcgcggtattatcccgtattgacgccgggcaagagcaactcggtcgccgcatacactattctcagaatgacttggttgagtactcaccagtcacagaaaagcatcttacggatggcatgacagtaagagaattatgcagtgctgccataaccatgagtgataacactgcggccaacttacttctgacaacgatcggaggaccgaaggagctaaccgcttttttgcacaacatgggggatcatgtaactcgccttgatcgttgggaaccggagctgaatgaagccataccaaacgacgagcgtgacaccacgatgcctgtagcaatggcaacaacgttgcgcaaactattaactggcgaactacttactctagcttcccggcaacaattaatagactggatggaggcggataaagttgcaggaccacttctgcgctcggcccttccggctggctggtttattgctgataaatctggagccggtgagcgtggaagccgcggtatcattgcagcactggggccagatggtaagccctcccgtatcgtagttatctacacgacggggagtcaggcaactatggatgaacgaaatagacagatcgctgagataggtgcctcactgattaagcattggtaactgtcagaccaagtttactcatatatactttagattgatttaaaacttcatttttaatttaaaaggatctaggtgaagatcctttttgataatctcatgaccaaaatcccttaacgtgagttttcgttccactgagcgtcagaccccgtagaaaagatcaaaggatcttcttgagatcctttttttctgcgcgtaatctgctgcttgcaaacaaaaaaaccaccgctaccagcggtggtttgtttgccggatcaagagctaccaactctttttccgaaggtaactggcttcagcagagcgcagataccaaatactgttcttctagtgtagccgtagttaggccaccacttcaagaactctgtagcaccgcctacatacctcgctctgctaatcctgttaccagtggctgctgccagtggcgataagtcgtgtcttaccgggttggactcaagacgatagttaccggataaggcgcagcggtcgggctgaacggggggttcgtgcacacagcccagcttggagcgaacgacctacaccgaactgagatacctacagcgtgagctatgagaaagcgccacgcttcccgaagggagaaaggcggacaggtatccggtaagcggcagggtcggaacaggagagcgcacgagggagcttccagggggaaacgcctggtatctttatagtcctgtcgggtttcgccacctctgacttgagcgtcgatttttgtgatgctcgtcaggggggcggagcctatggaaaaacgccagcaacgcggcctttttacggttcctggccttttgctggccttttgctcacatgt


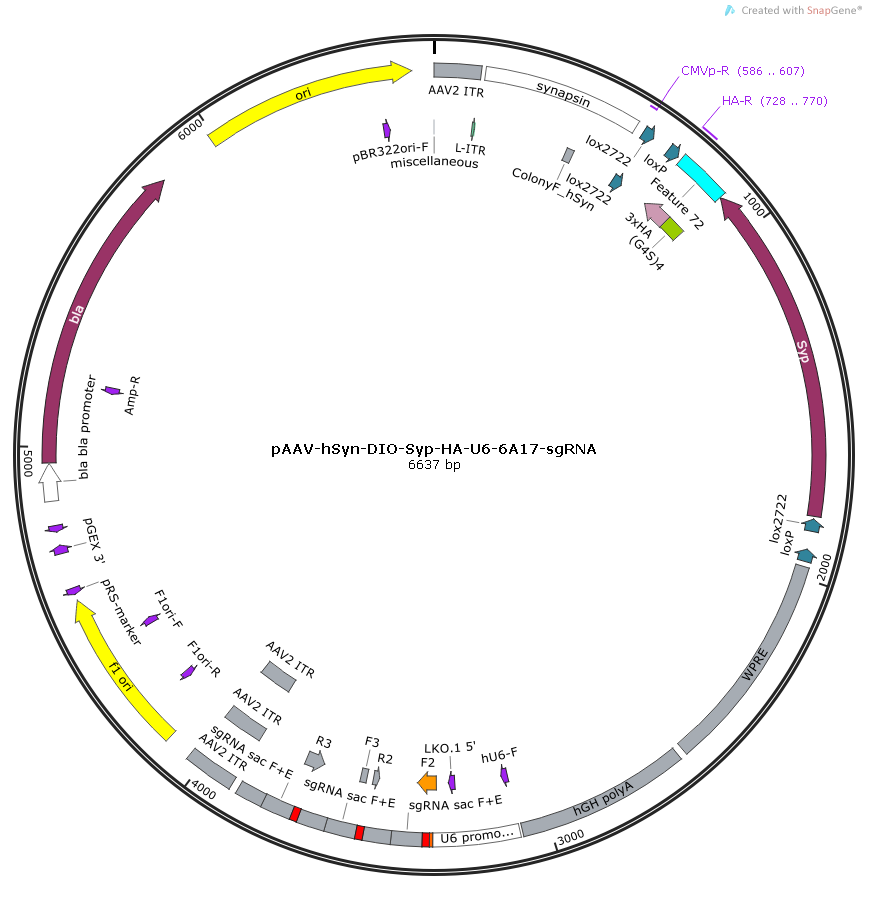


pAAV-hSyn-SLC6A17-APEX2-WPRE

catgtcctgcaggcagctgcgcgctcgctcgctcactgaggccgcccgggcgtcgggcgacctttggtcgcccggcctcagtgagcgagcgagcgcgcagagagggagtggccaactccatcactaggggttcctgcggccgcacgcgtgtgtctagactgcagagggccctgcgtatgagtgcaagtgggttttaggaccaggatgaggcggggtgggggtgcctacctgacgaccgaccccgacccactggacaagcacccaacccccattccccaaattgcgcatcccctatcagagagggggaggggaaacaggatgcggcgaggcgcgtgcgcactgccagcttcagcaccgcggacagtgccttcgcccccgcctggcggcgcgcgccaccgccgcctcagcactgaaggcgcgctgacgtcactcgccggtcccccgcaaactccccttcccggccaccttggtcgcgtccgcgccgccgccggcccagccggaccgcaccacgcgaggcgcgagataggggggcacgggcgcgaccatctgcgctgcggcgccggcgactcagcgctgcctcagtctgcggtgggcagcggaggagtcgtgtcgtgcctgagagcgcagtcgagaaggtaccaatgccgaagaacagcaaagtgacccagcgtgagcacagcagtgagcatgtcactgagtccgtggccgacctgctggccctcgaggagcctgtggactataagcagagtgtactgaatgtggctggtgaggcaggcggcaagcagaaggcggtggaggaggagctggatgcagaggaccggccggcctggaacagtaagctgcagtacatcctggcccagattggcttctctgtgggcctcggcaacatctggaggttcccctacctgtgccagaaaaatggaggaggtgcttacctggtgccctacctggtgctgctgatcatcatcgggatccccctcttcttcctggagctggctgtgggtcagaggatccgccgcggcagcatcggtgtgtggcactatatatgtccccgcctggggggcatcggcttctccagctgcatagtctgtctctttgtggggctgtattataatgtgatcatcgggtggagcatcttctatttcttcaagtccttccagtacccgctgccctggagtgaatgtcctgtcgtcaggaatgggagcgtggcagtggtggaggcagagtgtgaaaagagctcagccactacctacttctggtaccgagaggccttggacatctctgactccatctcggagagtgggggcctcaactggaagatgaccctgtgcctcctcgtggcctggagcatcgtggggatggctgtcgttaagggcatccagtcctcggggaaggtgatgtatttcagctccctcttcccctacgtggtgctggcctgcttcctggtccgggggctgttgctgcgaggggcagttgatggcatcctacacatgttcactcccaagctggacaagatgctggacccccaggtgtggcgggaggcagctacccaggtcttctttgccttgggcctgggctttggtggtgtcattgccttctccagctacaataagcaggacaacaactgccacttcgatgccgccctggtgtccttcatcaacttcttcacgtcagtgttggccaccctcgtggtgtttgctgtgctgggcttcaaggccaacatcatgaatgagaagtgtgtggtcgagaatgctgagaaaatcctagggtaccttaacaccaacgtcctgagccgggacctcatcccaccccacgtcaacttctcccacctgaccacaaaggactacatggagatgtacaatgtcatcatgaccgtgaaggaggaccagttctcagccctgggccttgacccctgccttctggaggacgagctggacaagtccgtgcagggcacaggcctggccttcatcgccttcactgaggccatgacgcacttccccgcctccccgttctggtccgtcatgttcttcttgatgcttatcaacctgggcctgggcagcatgatcgggaccatggcaggcatcaccacgcccatcatcgacaccttcaaggtgcccaaggagatgttcacagtgggctgctgtgtctttgcattcctcgtggggctgttgttcgtccagcgctccggaaactactttgtcaccatgttcgatgactactcggccaccctgccactcactctcatcgtcatccttgagaacatcgctgtggcctggatttatggaaccaagaagttcatgcaggagctgacggagatgctgggcttccgcccctaccgcttctatttctacatgtggaagttcgtgtctccactatgcatggctgtgctcaccacagccagcatcatccagctgggggtcacgcccccgggctacagcgcctggatcaaggaggaggctgccgagcgctacctgtatttccccaactgggccatggcactcctgatcaccctcatcgtcgtggcgacgctgcccatccctgtggtgttcgtcctgcggcacttccacctgctctctgatggctccaacaccctctccgtgtcctacaagaagggccgcatgatgaaggacatctccaacctggaggagaacgatgagacccgcttcatcctcagcaaggtgcccagtgaggcaccttcccccatgcccactcaccgttcctatctggggcccggcagcacatcacccctggagaccagcggtaaccccaatggacgctatgggagcggctacctgctggccagcacccctgagtcggagctgggtggtggtggtggtggtggttacccatacgatgttccagattacgcttatccctacgacgtgcctgattatgcatacccatatgatgtccccgactatgccggtaccGGCAAGCCCATCCCCAACCCCCTGCTGGGCCTGGACAGCACCGGAAAGTCTTACCCAACTGTGAGTGCTGATTACCAGGACGCCGTTGAGAAGGCGAAGAAGAAGCTCAGAGGCTTCATCGCTGAGAAGAGATGCGCTCCTCTAATGCTCCGTTTGGCATTCCACTCTGCTGGAACCTTTGACAAGGGCACGAAGACCGGTGGACCCTTCGGAACCATCAAGCACCCTGCCGAACTGGCTCACAGCGCTAACAACGGTCTTGACATCGCTGTTAGGCTTTTGGAGCCACTCAAGGCGGAGTTCCCTATTTTGAGCTACGCCGATTTCTACCAGTTGGCTGGCGTTGTTGCCGTTGAGGTCACGGGTGGACCTAAGGTTCCATTCCACCCTGGAAGAGAGGACAAGCCTGAGCCACCACCAGAGGGTCGCTTGCCCGATCCCACTAAGGGTTCTGACCATTTGAGAGATGTGTTTGGCAAAGCTATGGGGCTTACTGACCAAGATATCGTTGCTCTATCTGGGGGTCACACTATTGGAGCTGCACACAAGGAGCGTTCTGGATTTGAGGGTCCCTGGACCTCTAATCCTCTTATTTTCGACAACTCATACTTCACGGAGTTGTTGAGTGGTGAGAAGGAAGGTCTCCTTCAGCTACCTTCTGACAAGGCTCTTTTGTCTGACCCTGTATTCCGCCCTCTCGTTGATAAATATGCAGCGGACGAAGATGCCTTCTTTGCTGATTACGCTGAGGCTCACCAAAAGCTTTCCGAGCTTGGGTTTGCTGATGCCTAAgaattcgatatcaagcttatcgataatcaacctctggattacaaaatttgtgaaagattgactggtattcttaactatgttgctccttttacgctatgtggatacgctgctttaatgcctttgtatcatgctattgcttcccgtatggctttcattttctcctccttgtataaatcctggttgctgtctctttatgaggagttgtggcccgttgtcaggcaacgtggcgtggtgtgcactgtgtttgctgacgcaacccccactggttggggcattgccaccacctgtcagctcctttccgggactttcgctttccccctccctattgccacggcggaactcatcgccgcctgccttgcccgctgctggacaggggctcggctgttgggcactgacaattccgtggtgttgtcggggaaatcatcgtcctttccttggctgctcgcctatgttgccacctggattctgcgcgggacgtccttctgctacgtcccttcggccctcaatccagcggaccttccttcccgcggcctgctgccggctctgcggcctcttccgcgtcttcgccttcgccctcagacgagtcggatctccctttgggccgcctccccgcatcgataccgagcgctgctcgagagatctacgggtggcatccctgtgacccctccccagtgcctctcctggccctggaagttgccactccagtgcccaccagccttgtcctaataaaattaagttgcatcattttgtctgactaggtgtccttctataatattatggggtggaggggggtggtatggagcaaggggcaagttgggaagacaacctgtagggcctgcggggtctattgggaaccaagctggagtgcagtggcacaatcttggctcactgcaatctccgcctcctgggttcaagcgattctcctgcctcagcctcccgagttgttgggattccaggcatgcatgaccaggctcagctaatttttgtttttttggtagagacggggtttcaccatattggccaggctggtctccaactcctaatctcaggtgatctacccaccttggcctcccaaattgctgggattacaggcgtgaaccactgctcccttccctgtccttctgattttgtaggtaaccacgtgcggaccgagcggccgcaggaacccctagtgatggagttggccactccctctctgcgcgctcgctcgctcactgaggccgggcgaccaaaggtcgcccgacgcccgggctttgcccgggcggcctcagtgagcgagcgagcgcgcagctgcctgcaggggcgcctgatgcggtattttctccttacgcatctgtgcggtatttcacaccgcatacgtcaaagcaaccatagtacgcgccctgtagcggcgcattaagcgcggcgggtgtggtggttacgcgcagcgtgaccgctacacttgccagcgccttagcgcccgctcctttcgctttcttcccttcctttctcgccacgttcgccggctttccccgtcaagctctaaatcgggggctccctttagggttccgatttagtgctttacggcacctcgaccccaaaaaacttgatttgggtgatggttcacgtagtgggccatcgccctgatagacggtttttcgccctttgacgttggagtccacgttctttaatagtggactcttgttccaaactggaacaacactcaactctatctcgggctattcttttgatttataagggattttgccgatttcggtctattggttaaaaaatgagctgatttaacaaaaatttaacgcgaattttaacaaaatattaacgtttacaattttatggtgcactctcagtacaatctgctctgatgccgcatagttaagccagccccgacacccgccaacacccgctgacgcgccctgacgggcttgtctgctcccggcatccgcttacagacaagctgtgaccgtctccgggagctgcatgtgtcagaggttttcaccgtcatcaccgaaacgcgcgagacgaaagggcctcgtgatacgcctatttttataggttaatgtcatgataataatggtttcttagacgtcaggtggcacttttcggggaaatgtgcgcggaacccctatttgtttatttttctaaatacattcaaatatgtatccgctcatgagacaataaccctgataaatgcttcaataatattgaaaaaggaagagtatgagtattcaacatttccgtgtcgcccttattcccttttttgcggcattttgccttcctgtttttgctcacccagaaacgctggtgaaagtaaaagatgctgaagatcagttgggtgcacgagtgggttacatcgaactggatctcaacagcggtaagatccttgagagttttcgccccgaagaacgttttccaatgatgagcacttttaaagttctgctatgtggcgcggtattatcccgtattgacgccgggcaagagcaactcggtcgccgcatacactattctcagaatgacttggttgagtactcaccagtcacagaaaagcatcttacggatggcatgacagtaagagaattatgcagtgctgccataaccatgagtgataacactgcggccaacttacttctgacaacgatcggaggaccgaaggagctaaccgcttttttgcacaacatgggggatcatgtaactcgccttgatcgttgggaaccggagctgaatgaagccataccaaacgacgagcgtgacaccacgatgcctgtagcaatggcaacaacgttgcgcaaactattaactggcgaactacttactctagcttcccggcaacaattaatagactggatggaggcggataaagttgcaggaccacttctgcgctcggcccttccggctggctggtttattgctgataaatctggagccggtgagcgtgggtctcgcggtatcattgcagcactggggccagatggtaagccctcccgtatcgtagttatctacacgacggggagtcaggcaactatggatgaacgaaatagacagatcgctgagataggtgcctcactgattaagcattggtaactgtcagaccaagtttactcatatatactttagattgatttaaaacttcatttttaatttaaaaggatctaggtgaagatcctttttgataatctcatgaccaaaatcccttaacgtgagttttcgttccactgagcgtcagaccccgtagaaaagatcaaaggatcttcttgagatcctttttttctgcgcgtaatctgctgcttgcaaacaaaaaaaccaccgctaccagcggtggtttgtttgccggatcaagagctaccaactctttttccgaaggtaactggcttcagcagagcgcagataccaaatactgttcttctagtgtagccgtagttaggccaccacttcaagaactctgtagcaccgcctacatacctcgctctgctaatcctgttaccagtggctgctgccagtggcgataagtcgtgtcttaccgggttggactcaagacgatagttaccggataaggcgcagcggtcgggctgaacggggggttcgtgcacacagcccagcttggagcgaacgacctacaccgaactgagatacctacagcgtgagctatgagaaagcgccacgcttcccgaagggagaaaggcggacaggtatccggtaagcggcagggtcggaacaggagagcgcacgagggagcttccagggggaaacgcctggtatctttatagtcctgtcgggtttcgccacctctgacttgagcgtcgatttttgtgatgctcgtcaggggggcggagcctatggaaaaacgccagcaacgcggcctttttacggttcctggccttttgctggccttttgctca


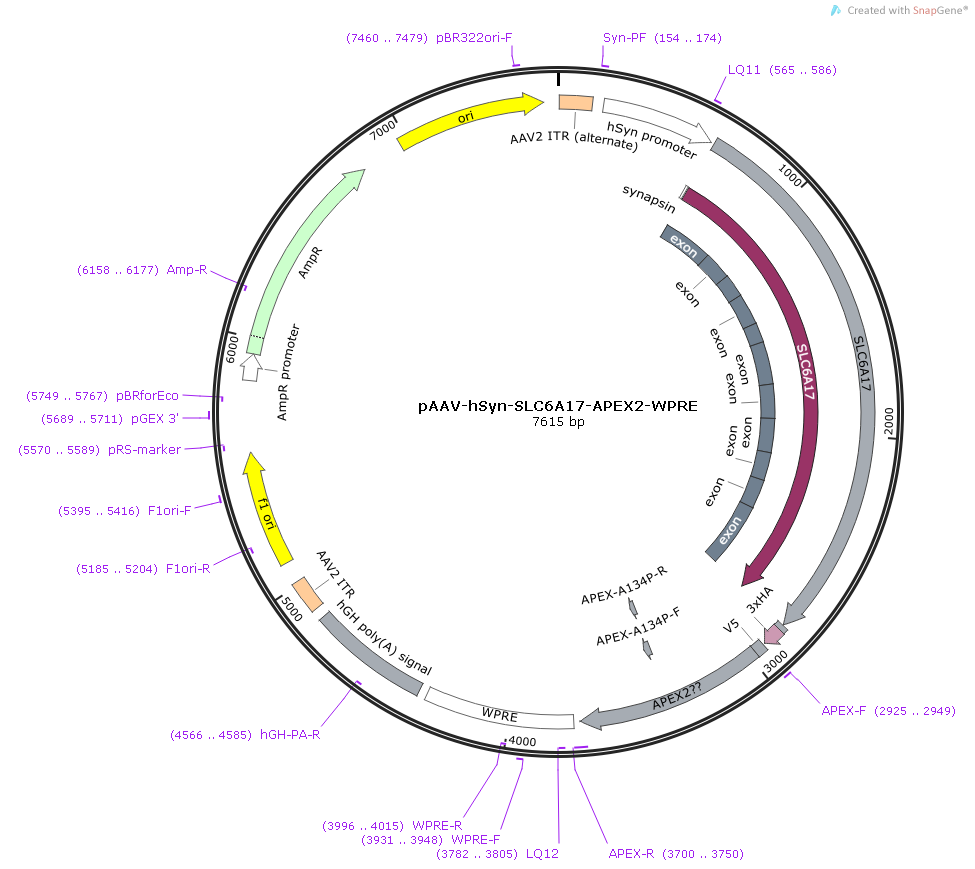


pAAV-hSyn-SLC6A17-G162A-HA-WPRE

catgtcctgcaggcagctgcgcgctcgctcgctcactgaggccgcccgggcgtcgggcgacctttggtcgcccggcctcagtgagcgagcgagcgcgcagagagggagtggccaactccatcactaggggttcctgcggccgcacgcgtgtgtctagactgcagagggccctgcgtatgagtgcaagtgggttttaggaccaggatgaggcggggtgggggtgcctacctgacgaccgaccccgacccactggacaagcacccaacccccattccccaaattgcgcatcccctatcagagagggggaggggaaacaggatgcggcgaggcgcgtgcgcactgccagcttcagcaccgcggacagtgccttcgcccccgcctggcggcgcgcgccaccgccgcctcagcactgaaggcgcgctgacgtcactcgccggtcccccgcaaactccccttcccggccaccttggtcgcgtccgcgccgccgccggcccagccggaccgcaccacgcgaggcgcgagataggggggcacgggcgcgaccatctgcgctgcggcgccggcgactcagcgctgcctcagtctgcggtgggcagcggaggagtcgtgtcgtgcctgagagcgcagtcgagaaggtaccatgccgaagaacagcaaagtgacccagcgtgagcacagcagtgagcatgtcactgagtccgtggccgacctgctggccctcgaggagcctgtggactataagcagagtgtactgaatgtggctggtgaggcaggcggcaagcagaaggcggtggaggaggagctggatgcagaggaccggccggcctggaacagtaagctgcagtacatcctggcccagattggcttctctgtgggcctcggcaacatctggaggttcccctacctgtgccagaaaaatggaggaggtgcttacctggtgccctacctggtgctgctgatcatcatcgggatccccctcttcttcctggagctggctgtgggtcagaggatccgccgcggcagcatcggtgtgtggcactatatatgtccccgcctggggggcatcggcttctccagctgcatagtctgtctctttgtggggctgtattataatgtgatcatcAggtggagcatcttctatttcttcaagtccttccagtacccgctgccctggagtgaatgtcctgtcgtcaggaatgggagcgtggcagtggtggaggcagagtgtgaaaagagctcagccactacctacttctggtaccgagaggccttggacatctctgactccatctcggagagtgggggcctcaactggaagatgaccctgtgcctcctcgtggcctggagcatcgtggggatggctgtcgttaagggcatccagtcctcggggaaggtgatgtatttcagctccctcttcccctacgtggtgctggcctgcttcctggtccgggggctgttgctgcgaggggcagttgatggcatcctacacatgttcactcccaagctggacaagatgctggacccccaggtgtggcgggaggcagctacccaggtcttctttgccttgggcctgggctttggtggtgtcattgccttctccagctacaataagcaggacaacaactgccacttcgatgccgccctggtgtccttcatcaacttcttcacgtcagtgttggccaccctcgtggtgtttgctgtgctgggcttcaaggccaacatcatgaatgagaagtgtgtggtcgagaatgctgagaaaatcctagggtaccttaacaccaacgtcctgagccgggacctcatcccaccccacgtcaacttctcccacctgaccacaaaggactacatggagatgtacaatgtcatcatgaccgtgaaggaggaccagttctcagccctgggccttgacccctgccttctggaggacgagctggacaagtccgtgcagggcacaggcctggccttcatcgccttcactgaggccatgacgcacttccccgcctccccgttctggtccgtcatgttcttcttgatgcttatcaacctgggcctgggcagcatgatcgggaccatggcaggcatcaccacgcccatcatcgacaccttcaaggtgcccaaggagatgttcacagtgggctgctgtgtctttgcattcctcgtggggctgttgttcgtccagcgctccggaaactactttgtcaccatgttcgatgactactcggccaccctgccactcactctcatcgtcatccttgagaacatcgctgtggcctggatttatggaaccaagaagttcatgcaggagctgacggagatgctgggcttccgcccctaccgcttctatttctacatgtggaagttcgtgtctccactatgcatggctgtgctcaccacagccagcatcatccagctgggggtcacgcccccgggctacagcgcctggatcaaggaggaggctgccgagcgctacctgtatttccccaactgggccatggcactcctgatcaccctcatcgtcgtggcgacgctgcccatccctgtggtgttcgtcctgcggcacttccacctgctctctgatggctccaacaccctctccgtgtcctacaagaagggccgcatgatgaaggacatctccaacctggaggagaacgatgagacccgcttcatcctcagcaaggtgcccagtgaggcaccttcccccatgcccactcaccgttcctatctggggcccggcagcacatcacccctggagaccagcggtaaccccaatggacgctatgggagcggctacctgctggccagcacccctgagtcggagctgggtggtggtggtggtggtggttacccatacgatgttccagattacgcttatccctacgacgtgcctgattatgcatacccatatgatgtccccgactatgcctaagaattcgatatcaagcttatcgataatcaacctctggattacaaaatttgtgaaagattgactggtattcttaactatgttgctccttttacgctatgtggatacgctgctttaatgcctttgtatcatgctattgcttcccgtatggctttcattttctcctccttgtataaatcctggttgctgtctctttatgaggagttgtggcccgttgtcaggcaacgtggcgtggtgtgcactgtgtttgctgacgcaacccccactggttggggcattgccaccacctgtcagctcctttccgggactttcgctttccccctccctattgccacggcggaactcatcgccgcctgccttgcccgctgctggacaggggctcggctgttgggcactgacaattccgtggtgttgtcggggaaatcatcgtcctttccttggctgctcgcctatgttgccacctggattctgcgcgggacgtccttctgctacgtcccttcggccctcaatccagcggaccttccttcccgcggcctgctgccggctctgcggcctcttccgcgtcttcgccttcgccctcagacgagtcggatctccctttgggccgcctccccgcatcgataccgagcgctgctcgagagatctacgggtggcatccctgtgacccctccccagtgcctctcctggccctggaagttgccactccagtgcccaccagccttgtcctaataaaattaagttgcatcattttgtctgactaggtgtccttctataatattatggggtggaggggggtggtatggagcaaggggcaagttgggaagacaacctgtagggcctgcggggtctattgggaaccaagctggagtgcagtggcacaatcttggctcactgcaatctccgcctcctgggttcaagcgattctcctgcctcagcctcccgagttgttgggattccaggcatgcatgaccaggctcagctaatttttgtttttttggtagagacggggtttcaccatattggccaggctggtctccaactcctaatctcaggtgatctacccaccttggcctcccaaattgctgggattacaggcgtgaaccactgctcccttccctgtccttctgattttgtaggtaaccacgtgcggaccgagcggccgcaggaacccctagtgatggagttggccactccctctctgcgcgctcgctcgctcactgaggccgggcgaccaaaggtcgcccgacgcccgggctttgcccgggcggcctcagtgagcgagcgagcgcgcagctgcctgcaggggcgcctgatgcggtattttctccttacgcatctgtgcggtatttcacaccgcatacgtcaaagcaaccatagtacgcgccctgtagcggcgcattaagcgcggcgggtgtggtggttacgcgcagcgtgaccgctacacttgccagcgccttagcgcccgctcctttcgctttcttcccttcctttctcgccacgttcgccggctttccccgtcaagctctaaatcgggggctccctttagggttccgatttagtgctttacggcacctcgaccccaaaaaacttgatttgggtgatggttcacgtagtgggccatcgccctgatagacggtttttcgccctttgacgttggagtccacgttctttaatagtggactcttgttccaaactggaacaacactcaactctatctcgggctattcttttgatttataagggattttgccgatttcggtctattggttaaaaaatgagctgatttaacaaaaatttaacgcgaattttaacaaaatattaacgtttacaattttatggtgcactctcagtacaatctgctctgatgccgcatagttaagccagccccgacacccgccaacacccgctgacgcgccctgacgggcttgtctgctcccggcatccgcttacagacaagctgtgaccgtctccgggagctgcatgtgtcagaggttttcaccgtcatcaccgaaacgcgcgagacgaaagggcctcgtgatacgcctatttttataggttaatgtcatgataataatggtttcttagacgtcaggtggcacttttcggggaaatgtgcgcggaacccctatttgtttatttttctaaatacattcaaatatgtatccgctcatgagacaataaccctgataaatgcttcaataatattgaaaaaggaagagtatgagtattcaacatttccgtgtcgcccttattcccttttttgcggcattttgccttcctgtttttgctcacccagaaacgctggtgaaagtaaaagatgctgaagatcagttgggtgcacgagtgggttacatcgaactggatctcaacagcggtaagatccttgagagttttcgccccgaagaacgttttccaatgatgagcacttttaaagttctgctatgtggcgcggtattatcccgtattgacgccgggcaagagcaactcggtcgccgcatacactattctcagaatgacttggttgagtactcaccagtcacagaaaagcatcttacggatggcatgacagtaagagaattatgcagtgctgccataaccatgagtgataacactgcggccaacttacttctgacaacgatcggaggaccgaaggagctaaccgcttttttgcacaacatgggggatcatgtaactcgccttgatcgttgggaaccggagctgaatgaagccataccaaacgacgagcgtgacaccacgatgcctgtagcaatggcaacaacgttgcgcaaactattaactggcgaactacttactctagcttcccggcaacaattaatagactggatggaggcggataaagttgcaggaccacttctgcgctcggcccttccggctggctggtttattgctgataaatctggagccggtgagcgtgggtctcgcggtatcattgcagcactggggccagatggtaagccctcccgtatcgtagttatctacacgacggggagtcaggcaactatggatgaacgaaatagacagatcgctgagataggtgcctcactgattaagcattggtaactgtcagaccaagtttactcatatatactttagattgatttaaaacttcatttttaatttaaaaggatctaggtgaagatcctttttgataatctcatgaccaaaatcccttaacgtgagttttcgttccactgagcgtcagaccccgtagaaaagatcaaaggatcttcttgagatcctttttttctgcgcgtaatctgctgcttgcaaacaaaaaaaccaccgctaccagcggtggtttgtttgccggatcaagagctaccaactctttttccgaaggtaactggcttcagcagagcgcagataccaaatactgttcttctagtgtagccgtagttaggccaccacttcaagaactctgtagcaccgcctacatacctcgctctgctaatcctgttaccagtggctgctgccagtggcgataagtcgtgtcttaccgggttggactcaagacgatagttaccggataaggcgcagcggtcgggctgaacggggggttcgtgcacacagcccagcttggagcgaacgacctacaccgaactgagatacctacagcgtgagctatgagaaagcgccacgcttcccgaagggagaaaggcggacaggtatccggtaagcggcagggtcggaacaggagagcgcacgagggagcttccagggggaaacgcctggtatctttatagtcctgtcgggtttcgccacctctgacttgagcgtcgatttttgtgatgctcgtcaggggggcggagcctatggaaaaacgccagcaacgcggcctttttacggttcctggccttttgctggccttttgctca


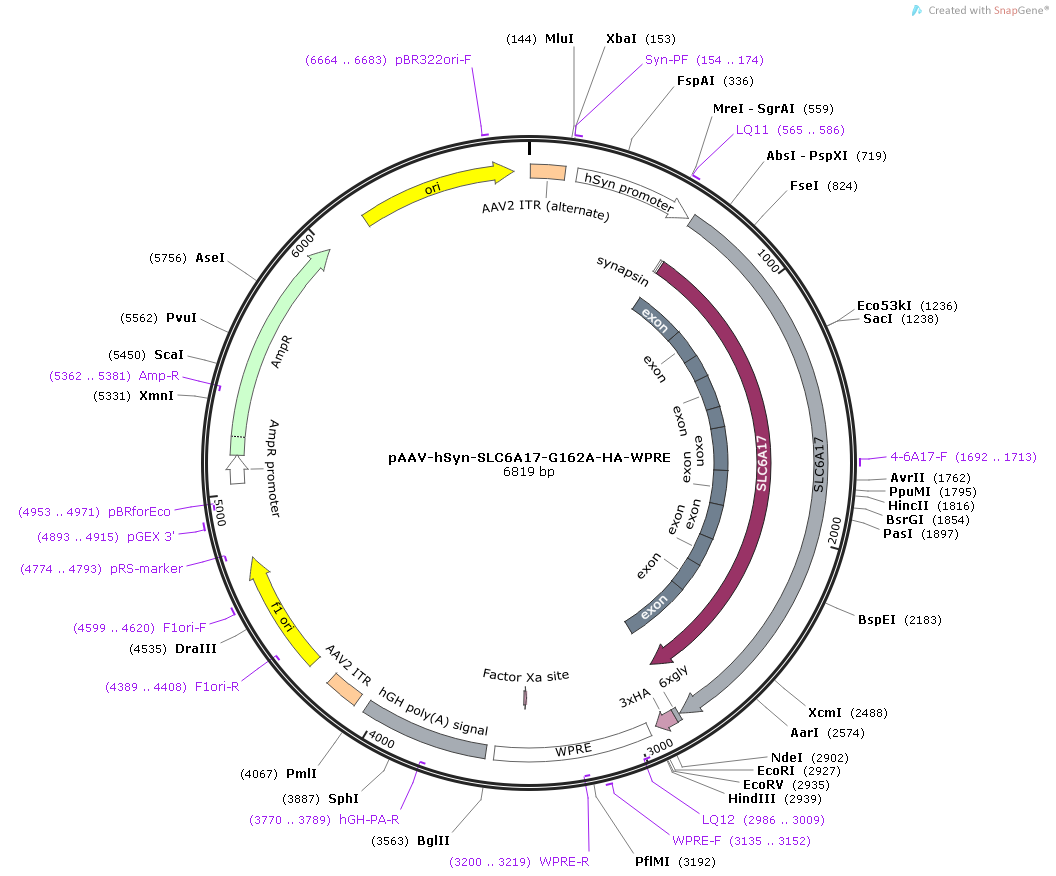


pAAV-hSyn-SLC6A17-HA

cctgcaggcagctgcgcgctcgctcgctcactgaggccgcccgggcgtcgggcgacctttggtcgcccggcctcagtgagcgagcgagcgcgcagagagggagtggccaactccatcactaggggttcctgcggcctctagaagtgcaagtgggttttaggaccaggatgaggcggggtgggggtgcctacctgacgaccgaccccgacccactggacaagcacccaacccccattccccaaattgcgcatcccctatcagagagggggaggggaaacaggatgcggcgaggcgcgtgcgcactgccagcttcagcaccgcggacagtgccttcgcccccgcctggcggcgcgcgccaccgccgcctcagcactgaaggcgcgctgacgtcactcgccggtcccccgcaaactccccttcccggccaccttggtcgcgtccgcgccgccgccggcccagccggaccgcaccacgcgaggcgcgagataggggggcacgggcgcgaccatctgcgctgcggcgccggcgactcagcgctgcctcagtctgcggtgggcagcggaggagtcgtgtcgtgcctgagagcgcaggagctctctggctaactaccggtaatgccgaagaacagcaaagtgacccagcgtgagcacagcagtgagcatgtcactgagtccgtggccgacctgctggccctcgaggagcctgtggactataagcagagtgtactgaatgtggctggtgaggcaggcggcaagcagaaggcggtggaggaggagctggatgcagaggaccggccggcctggaacagtaagctgcagtacatcctggcccagattggcttctctgtgggcctcggcaacatctggaggttcccctacctgtgccagaaaaatggaggaggtgcttacctggtgccctacctggtgctgctgatcatcatcgggatccccctcttcttcctggagctggctgtgggtcagaggatccgccgcggcagcatcggtgtgtggcactatatatgtccccgcctggggggcatcggcttctccagctgcatagtctgtctctttgtggggctgtattataatgtgatcatcgggtggagcatcttctatttcttcaagtccttccagtacccgctgccctggagtgaatgtcctgtcgtcaggaatgggagcgtggcagtggtggaggcagagtgtgaaaagagctcagccactacctacttctggtaccgagaggccttggacatctctgactccatctcggagagtgggggcctcaactggaagatgaccctgtgcctcctcgtggcctggagcatcgtggggatggctgtcgttaagggcatccagtcctcggggaaggtgatgtatttcagctccctcttcccctacgtggtgctggcctgcttcctggtccgggggctgttgctgcgaggggcagttgatggcatcctacacatgttcactcccaagctggacaagatgctggacccccaggtgtggcgggaggcagctacccaggtcttctttgccttgggcctgggctttggtggtgtcattgccttctccagctacaataagcaggacaacaactgccacttcgatgccgccctggtgtccttcatcaacttcttcacgtcagtgttggccaccctcgtggtgtttgctgtgctgggcttcaaggccaacatcatgaatgagaagtgtgtggtcgagaatgctgagaaaatcctagggtaccttaacaccaacgtcctgagccgggacctcatcccaccccacgtcaacttctcccacctgaccacaaaggactacatggagatgtacaatgtcatcatgaccgtgaaggaggaccagttctcagccctgggccttgacccctgccttctggaggacgagctggacaagtccgtgcagggcacaggcctggccttcatcgccttcactgaggccatgacgcacttccccgcctccccgttctggtccgtcatgttcttcttgatgcttatcaacctgggcctgggcagcatgatcgggaccatggcaggcatcaccacgcccatcatcgacaccttcaaggtgcccaaggagatgttcacagtgggctgctgtgtctttgcattcctcgtggggctgttgttcgtccagcgctccggaaactactttgtcaccatgttcgatgactactcggccaccctgccactcactctcatcgtcatccttgagaacatcgctgtggcctggatttatggaaccaagaagttcatgcaggagctgacggagatgctgggcttccgcccctaccgcttctatttctacatgtggaagttcgtgtctccactatgcatggctgtgctcaccacagccagcatcatccagctgggggtcacgcccccgggctacagcgcctggatcaaggaggaggctgccgagcgctacctgtatttccccaactgggccatggcactcctgatcaccctcatcgtcgtggcgacgctgcccatccctgtggtgttcgtcctgcggcacttccacctgctctctgatggctccaacaccctctccgtgtcctacaagaagggccgcatgatgaaggacatctccaacctggaggagaacgatgagacccgcttcatcctcagcaaggtgcccagtgaggcaccttcccccatgcccactcaccgttcctatctggggcccggcagcacatcacccctggagaccagcggtaaccccaatggacgctatgggagcggctacctgctggccagcacccctgagtcggagctgggtggtggtggtggtggtggttacccatacgatgttccagattacgcttatccctacgacgtgcctgattatgcatacccatatgatgtccccgactatgccgatatcaagcttatcgataatcaacctctggattacaaaatttgtgaaagattgactggtattcttaactatgttgctccttttacgctatgtggatacgctgctttaatgcctttgtatcatgctattgcttcccgtatggctttcattttctcctccttgtataaatcctggttgctgtctctttatgaggagttgtggcccgttgtcaggcaacgtggcgtggtgtgcactgtgtttgctgacgcaacccccactggttggggcattgccaccacctgtcagctcctttccgggactttcgctttccccctccctattgccacggcggaactcatcgccgcctgccttgcccgctgctggacaggggctcggctgttgggcactgacaattccgtggtgttgtcggggaaatcatcgtcctttccttggctgctcgcctatgttgccacctggattctgcgcgggacgtccttctgctacgtcccttcggccctcaatccagcggaccttccttcccgcggcctgctgccggctctgcggcctcttccgcgtcttcgccttcgccctcagacgagtcggatctccctttgggccgcctccccgcatcgataccgagcgctgctcgagagatctacgggtggcatccctgtgacccctccccagtgcctctcctggccctggaagttgccactccagtgcccaccagccttgtcctaataaaattaagttgcatcattttgtctgactaggtgtccttctataatattatggggtggaggggggtggtatggagcaaggggcaagttgggaagacaacctgtagggcctgcggggtctattgggaaccaagctggagtgcagtggcacaatcttggctcactgcaatctccgcctcctgggttcaagcgattctcctgcctcagcctcccgagttgttgggattccaggcatgcatgaccaggctcagctaatttttgtttttttggtagagacggggtttcaccatattggccaggctggtctccaactcctaatctcaggtgatctacccaccttggcctcccaaattgctgggattacaggcgtgaaccactgctcccttccctgtccttgcggccgcgagggcctatttcccatgattccttcatatttgcatatacgatacaaggctgttagagagataattggaattaatttgactgtaaacacaaagatattagtacaaaatacgtgacgtagaaagtaataatttcttgggtagtttgcagttttaaaattatgttttaaaatggactatcatatgcttaccgtaacttgaaagtatttcgatttcttggctttatatatcttgtggaaaggacgaaacaccgggtacctttttgggcccgcggccgcaggaacccctagtgatggagttggccactccctctctgcgcgctcgctcgctcactgaggccgggcgaccaaaggtcgcccgacgcccgggctttgcccgggcggcctcagtgagcgagcgagcgcgcagctgcctgcaggggcgcctgatgcggtattttctccttacgcatctgtgcggtatttcacaccgcatacgtcaaagcaaccatagtacgcgccctgtagcggcgcattaagcgcggcgggtgtggtggttacgcgcagcgtgaccgctacacttgccagcgccttagcgcccgctcctttcgctttcttcccttcctttctcgccacgttcgccggctttccccgtcaagctctaaatcgggggctccctttagggttccgatttagtgctttacggcacctcgaccccaaaaaacttgatttgggtgatggttcacgtagtgggccatcgccctgatagacggtttttcgccctttgacgttggagtccacgttctttaatagtggactcttgttccaaactggaacaacactcaactctatctcgggctattcttttgatttataagggattttgccgatttcggtctattggttaaaaaatgagctgatttaacaaaaatttaacgcgaattttaacaaaatattaacgtttacaattttatggtgcactctcagtacaatctgctctgatgccgcatagttaagccagccccgacacccgccaacacccgctgacgcgccctgacgggcttgtctgctcccggcatccgcttacagacaagctgtgaccgtctccgggagctgcatgtgtcagaggttttcaccgtcatcaccgaaacgcgcgagacgaaagggcctcgtgatacgcctatttttataggttaatgtcatgataataatggtttcttagacgtcaggtggcacttttcggggaaatgtgcgcggaacccctatttgtttatttttctaaatacattcaaatatgtatccgctcatgagacaataaccctgataaatgcttcaataatattgaaaaaggaagagtatgagtattcaacatttccgtgtcgcccttattcccttttttgcggcattttgccttcctgtttttgctcacccagaaacgctggtgaaagtaaaagatgctgaagatcagttgggtgcacgagtgggttacatcgaactggatctcaacagcggtaagatccttgagagttttcgccccgaagaacgttttccaatgatgagcacttttaaagttctgctatgtggcgcggtattatcccgtattgacgccgggcaagagcaactcggtcgccgcatacactattctcagaatgacttggttgagtactcaccagtcacagaaaagcatcttacggatggcatgacagtaagagaattatgcagtgctgccataaccatgagtgataacactgcggccaacttacttctgacaacgatcggaggaccgaaggagctaaccgcttttttgcacaacatgggggatcatgtaactcgccttgatcgttgggaaccggagctgaatgaagccataccaaacgacgagcgtgacaccacgatgcctgtagcaatggcaacaacgttgcgcaaactattaactggcgaactacttactctagcttcccggcaacaattaatagactggatggaggcggataaagttgcaggaccacttctgcgctcggcccttccggctggctggtttattgctgataaatctggagccggtgagcgtggaagccgcggtatcattgcagcactggggccagatggtaagccctcccgtatcgtagttatctacacgacggggagtcaggcaactatggatgaacgaaatagacagatcgctgagataggtgcctcactgattaagcattggtaactgtcagaccaagtttactcatatatactttagattgatttaaaacttcatttttaatttaaaaggatctaggtgaagatcctttttgataatctcatgaccaaaatcccttaacgtgagttttcgttccactgagcgtcagaccccgtagaaaagatcaaaggatcttcttgagatcctttttttctgcgcgtaatctgctgcttgcaaacaaaaaaaccaccgctaccagcggtggtttgtttgccggatcaagagctaccaactctttttccgaaggtaactggcttcagcagagcgcagataccaaatactgttcttctagtgtagccgtagttaggccaccacttcaagaactctgtagcaccgcctacatacctcgctctgctaatcctgttaccagtggctgctgccagtggcgataagtcgtgtcttaccgggttggactcaagacgatagttaccggataaggcgcagcggtcgggctgaacggggggttcgtgcacacagcccagcttggagcgaacgacctacaccgaactgagatacctacagcgtgagctatgagaaagcgccacgcttcccgaagggagaaaggcggacaggtatccggtaagcggcagggtcggaacaggagagcgcacgagggagcttccagggggaaacgcctggtatctttatagtcctgtcgggtttcgccacctctgacttgagcgtcgatttttgtgatgctcgtcaggggggcggagcctatggaaaaacgccagcaacgcggcctttttacggttcctggccttttgctggccttttgctcacatgt


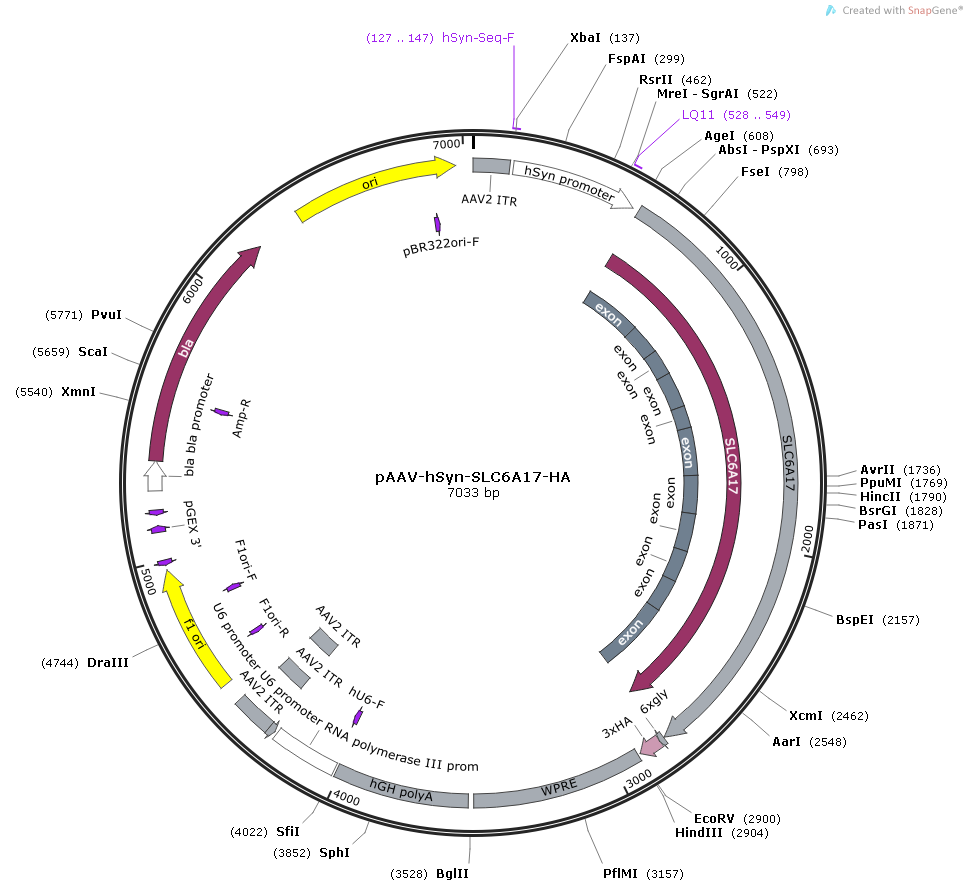

Supplement: Supplementary file 1. — pAAV-hSyn-SLC6A17-APEX2-WPRE-pA, pAAV-hSyn-SLC6A17-HA-WPRE-pA, pAAV-hSyn-SLC6A17G162R-HA-WPRE-pA, and pAAV-hSyn-DIO-Syp-HA-WPRE-pA-U6-3-t::gRNA WPRE-pA. Sequence information file for pAAV-hSyn-SLC6A17-APEX2-WPRE-pA. [file elife-86972-supp1.docx]
